# Effects of Consumption of Black Soybean Seed Coat Extract on Sleep Quality in Healthy Japanese: A Randomized, Placebo‐Controlled, Double‐Blind, Parallel‐Group Comparison Study

**DOI:** 10.1002/fsn3.70156

**Published:** 2025-06-11

**Authors:** Ryota Akagi, Toshinari Maruo, Tsuyoshi Takara, Kentaro Maruyama

**Affiliations:** ^1^ Innovation Center, Fujicco Co., Ltd. Kobe Hyogo Japan; ^2^ Medical Corporation Seishinkai, Takara Clinic Shinagawa‐ku Tokyo Japan

**Keywords:** antioxidant activity, black soybean, blood flow improvement, polyphenols, sleep

## Abstract

This study examined the effect of the intake of a test food containing black soybean seed coat extract (BE) on the sleep quality of healthy Japanese adults with poor sleep quality. This double‐blind, placebo‐controlled, randomized, parallel‐group trial included 94 participants. We randomly assigned 64 eligible participants to either (*n* = 32 per group) (i) the BE group receiving a BE‐containing capsule daily or (ii) the Placebo group receiving a placebo capsule daily. The primary outcome was sleepiness on rising score in the Oguri–Shirakawa–Azumi sleep inventory MA (OSA‐MA) at 12 weeks after intake, and the secondary outcomes were OSA‐MA, PSQI‐J, original questionnaire (visual analog scale), POMS2 scores, sleep test, blood flow, palmar surface temperature, and blood test data. The final analysis included data from 64 participants. The sleepiness on rising score was significantly higher in the BE group than in the placebo group. In the BE group, sleep‐onset latency and subjective dry mouth significantly decreased, and palmar surface temperature significantly increased. Furthermore, BE effectively reduced sleepiness on rising in men and increased the total sleep duration in women. For participants aged ≥ 40 years, BE improved age‐related decline in sleep quality and dry mouth. Increased deep sleep and reduced sleepiness on rising were observed in those with poor sleep quality. No adverse events were reported. BE intake promoted vasodilation and increased skin temperature, decreasing the sleep‐onset latency and significantly improving rising sleepiness. The mechanism is attributed to BE's antioxidant and autonomic neuromodulatory effects.

**Trial Registration:** UMIN‐CTR: UMIN000051261

## Graphical abstract

This study examined the effect of black soybean seed coat extract consumption on sleep quality in healthy Japanese adults who were dissatisfied with their sleep quality.

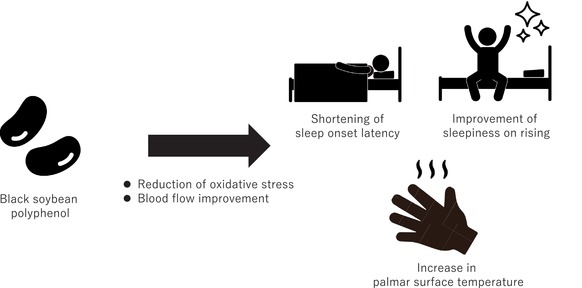

The black soybean seed coat extract intake significantly shortens sleep‐onset latency, relieves dry mouth sensations, and increases palmar surface temperature.

## 1 Introduction

Chronic sleep deprivation impairs stress tolerance and decision‐making and increases the risk of obesity, hypertension, diabetes, and cardiovascular diseases (Mader et al. 2022). Sleep deprivation results in increased work‐related injuries and traffic accidents, decreased productivity, and increased healthcare utilization (Mader et al. 2022). Insufficient sleep is estimated to cause a total economic loss of USD 680 billion per year in the United States, the United Kingdom, Germany, Canada, and Japan (Hafner et al. 2017). It is believed to be caused by (1) a decrease in the workforce due to death and illness, (2) reduced work efficiency due to poor performance, and (3) impediments to skill development, those resulting from sleep deprivation. Sleep deficiency is an important social issue. According to a survey by the Organization for Economic Cooperation and Development (OECD) in 2021, the average sleep duration of Japanese people was 7 h and 24 min, the lowest among 33 OECD member countries (OECD 2021), indicating that Japanese people have poor sleep quality on a global scale. According to the National Health and Nutrition Survey of Japan in 2019 (Ministry of Health Labor and Welfare 2019), approximately 30% of Japanese have no problems with sleep quality, whereas the rest complained of “feeling sleepy during the day,” “having trouble with awakening during sleep at night,” “not satisfied with overall sleep quality,” and “not enough sleep time.” Regarding physical and mental health, the importance of getting adequate sleep is mentioned in “Health Japan 21 (the third term),” a basic policy established by the Japanese government to comprehensively promote the health of the Japanese people (Ministry of Health Labour and Welfare 2023), and improving sleep quality is associated with improving quality of life (Matsui et al. 2021). Thus, the effects of food on sleep quality in healthy people must be investigated.

Black soybean ( *Glycine max* (L.) Merill) is known for its black seed coat and high nutritional content and is widely consumed in Asia. The seed coat of black soybean has been used as an herbal medicine since ancient times, and Chinese herbal medicine books have described its beneficial effects on blood flow (Namba 2018). The main constituents of black soybean seed coat extract (BE) are polyphenols, namely proanthocyanidins and epicatechins. These polyphenols have antioxidant properties (Akagi et al. 2024; Florentino et al. 2022; Ito et al. 2013; Kafer et al. 2023; Molinari et al. 2023; Wang et al. 2018). In a previous study, healthy Japanese men and women who experienced fatigue were administered 100 mg/day of BE for 4 weeks. They experienced reduced fatigue and temporary daytime sleepiness, which was attributed to the regulation of autonomic nerves by the antioxidant effect (Akagi et al. 2021). Indeed, antioxidative food ingredients other than BE could also improve sleep quality and reduce fatigue (Hao et al. 2024; Ochiai et al. 2018).

In humans, body temperature and sleep are related, and dorsal hand and foot skin temperatures increase relative to trunk skin temperature before sleep. The amplitude of this increase was positively correlated with sleepiness (Kräuchi et al. 1999). This increase in skin temperature for heat dissipation is caused by an increase in skin blood flow resulting from dilation of peripheral blood vessels (Van Someren 2006). Decreased parasympathetic activity, one of the autonomic nervous activities, is associated with decreased sleep quality (Werner et al. 2015), and the parasympathetic nervous system exhibits vasodilatory effects. Thus, when parasympathetic activity is decreased, blood vessels constrict, and skin temperature is unlikely to increase. Because BE is reported to improve endothelial function and blood flow (Namba 2018), blood vessel dilation and increased skin temperature are expected to promote smooth sleep onset and improve sleep quality. Therefore, in this study, we examined the effects of a 12‐week consumption of 100 mg/day of BE on sleep quality in healthy Japanese adults dissatisfied with sleep quality, and its effects on blood flow and skin temperature as secondary outcomes were evaluated.

## 2 Materials and Methods

### 2.1 Study Design and Ethical Statements

This randomized, double‐blind, placebo‐controlled, parallel‐group comparison study employed an allocation ratio of 1:1. The ethics committee of the Takara Clinic, Medical Corporation Seishinkai approved the study protocols on May 17, 2023 (Approval ID: 2305–00060‐0055‐16‐TC), and the protocol was registered at the University Hospital Medical Information Network Clinical Trials Registry (UMIN000051261). This study was conducted in accordance with the latest guidelines of the Declaration of Helsinki and the Ethical Guidelines for Medical and Biological Research Involving Human Subjects in Japan.

### 2.2 Participants

The inclusion criteria were as follows: participants (a) who were Japanese, (b) who were men or women, (c) aged ≥ 30 and < 65 years, (d) who were healthy individuals, (e) dissatisfied with their subjective fatigue upon waking and sleep quality (such as short sleep duration, difficulty falling asleep, difficulty sleeping soundly, dreaming, and persistent fatigue) in daily life, (f) who were judged eligible to participate in the study by the physician according to the results of the Beck depression inventory (BDI‐II) (Beck et al. 1996; Beck et al. 2003; Hiroe et al. 2005) at screening (Scr), (g) with low “sleepiness on rising” score in the Oguri–Shirakawa–Azumi sleep inventory MA (OSA‐MA) (Yamamoto et al. 1999) among those meeting (a)–(f) and not meeting the exclusion criteria at Scr.

The exclusion criteria were as follows: participants (a) undergoing medical treatment or with a history of malignant tumor, heart failure, or myocardial infarction; (b) having a pacemaker or an implantable cardioverter defibrillator; (c) currently undergoing treatment for cardiac arrhythmia, liver disorders, kidney disorders, cerebrovascular disorders, rheumatism, diabetes mellitus, dyslipidemia, hypertension, or other chronic diseases; (d) consuming “foods for specified health uses,” or “foods with functional claims” daily; (e) currently taking medications (including herbal medicines) and supplements; (f) with allergic to medicines and/or test food‐related products (particularly, soybean, pollen, or milk); (g) who are pregnant, lactating, or planning for pregnancy during the trial; (h) who were enrolled in other clinical trials during the last 28 days before agreeing to participate in this trial or planning to participate in another trial during this trial; (i) ineligible to participate in this study as judged by the physician; (j) living with their infants aged < 1 year; (k) sleeping with their children (1–6 years old); (l) living with and providing care for those in requiring long‐term care; (m) sleeping with more than one person; (n) with irregular sleeping times or habit because of work, such as a late‐night shift; (o) with nocturia ≥ 2; (p) having irregular lifestyles (such as inconsistent eating time and insufficient sleeping duration); (q) who excessively drink alcohol (Ministry of Health Labour and Welfare 2023) (average of > 20 g/day as absolute alcohol intake; 500 mL, a medium bottle of beer or approximately 1.5 cans of canned chu‐hi; 180 mL, 1 go of sake or approximately 1.5 glasses of wine; 90 mL, half‐go with shochu; 60 mL, a glass of whiskey/brandy [double]); (r) currently undergoing treatment for insomnia or sleep disorder; (s) consuming food/beverage containing functional ingredients that may influence sleep quality (such as gamma‐aminobutyric acid, crocetin, L‐theanine, and lactic acid bacteria).

All participants were recruited through an online website (https://www.go106.jp/) operated by ORTHOMEDICO Inc. (Tokyo, Japan). Individuals who provided written informed consent through the network after receiving a detailed explanation of the study were enrolled. No sponsors or members of the funding companies participated in the study. The Takara Clinic (Tokyo, Japan) was responsible for conducting the study. Data were acquired from the Medical Corporation Seishinkai, Takara Clinic, and the health of the study participants was monitored in this clinic.

### 2.3 Intervention

BE (ChronoCare SP60; Fujicco Co. Ltd., Hyogo, Japan) was prepared as previously reported (Akagi et al. 2024). Table 1 presents the raw material and BE compositions per capsule for each test food. The test foods were either hard capsules containing 100 mg (58 mg as total polyphenols) of ChronoCare SP60 (BE contained food) or hard capsules without ChronoCare SP60 (placebo). The test foods were filled into hard capsules size 2 and packaged in aluminum zip bags. Both test foods contained the same calorific value, protein, fat, carbohydrate, and salt equivalent content.

**TABLE 1:** Composition of test foods in each capsule.

Name	Form	Ingredient	Quantity
BE	Capsule	**Black soybean seed coat extract (ChronoCare SP60; details shown below)**	100 mg
		(Total polyphenols	58.08%)
		(Total flavanol	29.52%)
		(Epicatechin	4.07%)
		(Procyanidin B2	4.10%)
		(Procyanidin C1	1.35%)
		(Cinnamtannin A2	0.19%)
		**Pinedex#2 (dextrin)**	110 mg
		**NT.A.DK.CARAMEL OP (details shown below)**	63 mg
		(Gelatin	52.0 mg)
		(Caramel	1.1 mg)
		(Titanium dioxide	0.8 mg)
		(Water	9.1 mg)
Placebo	Capsule	**Pinedex#2 (dextrin)**	250 mg
		**NT.A.DK.CARAMEL OP (details shown below)**	63 mg
		(Gelatin	52.0 mg)
		(Caramel	1.1 mg)
		(Titanium dioxide	0.8 mg)
		(Water	9.1 mg)

The ethics committee confirmed the indistinguishability of the two types of capsules by their appearance. The participants consumed one capsule per day with water after dinner without chewing for 12 weeks.

### 2.4 Outcomes

Table 2 shows the study schedule. Efficacy outcomes were assessed at Scr, 4 weeks (4w), 8 weeks (8w), and 12 weeks (12w) after the start of the test food intake. Safety evaluation items were measured at Scr and 12w.

**TABLE 2:** Schedule of the study.

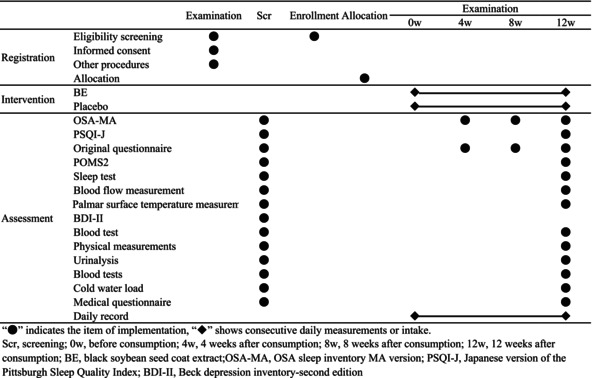

#### 2.4.1 Primary Outcome

The primary outcome was the measured value of sleepiness on rising (factor I) in OSA‐MA at 12w. To investigate the state of sleepiness upon waking, the OSA‐MA was administered immediately upon waking (before brushing teeth and washing face) for 3 days (2 and 1 days before 12w and the day of 12w). The mean value was calculated from the Zc scores for the 3 days of measurements, and higher Zc scores reflected better sleep.

#### 2.4.2 Secondary Outcomes

##### 2.4.2.1 OSA‐MA

The OSA‐MA was employed to investigate the state of sleep upon waking at Scr, 4w, 8w, and 12w. The measured values of sleepiness on rising at 4w and 8w and the amount and rate of change in sleepiness on rising from Scr to that at 4w, 8w, and 12w were evaluated. In addition, we evaluated the measured values and amount and rate of change from Scr of initiation and maintenance of sleep, frequent dreaming, refreshing, and sleep length, and each question on the OSA‐MA was evaluated at 4w, 8w, and 12w.

##### 2.4.2.2 Visual Analog Scale (VAS) of Subjective Symptoms

Subjective symptoms upon waking were investigated using the original questionnaire with VAS (Japanese Society for Fatigue Science 2011a, 2011b; McCormack et al. 1988) at Scr, 4w, 8w, and 12w. Survey items included fatigue sensation, sleepiness, stiff shoulders, motivation, eye fatigue sensation, sluggishness and heaviness of the body, coldness, ocular dryness, dry mouth, and stress. A 100‐mm straight line was printed on the questionnaire; the participants marked their current degree of sensation on the line, and the best imaginable state was assigned 0 and the worst state 100. The measured values of each question item in the original questionnaires at 4w, 8w, and 12w and the amount and rate of change from Scr for each measuring point were assessed. The participants answered the questionnaire immediately upon waking (before brushing teeth and washing face) for 3 days (2 and 1 days before each examination and the day of each examination). The mean value was calculated from the scores for the 3 days of measurements.

##### 2.4.2.3 Japanese Version of the Pittsburgh Sleep Quality Index (PSQI‐J) (Doi et al. 1998, 2000)

The PSQI‐J was used to assess daytime sleep conditions at Scr and 12w. The measured values and the amount and rate of change from Scr of the global PSQI score, subjective sleep quality (C1), sleep latency (C2), sleep duration (C3), habitual sleep efficiency (C4), sleep disturbances (C5), use of sleep medications (C6), daytime dysfunction (C7), and each question on the PSQI‐J at 12w were evaluated. Scores for C1–C7 were calculated according to the scoring method, and lower scores indicated better sleep status.

##### 2.4.2.4 Profile of Mood States 2nd Edition (POMS2) (Heuchert and McNair 2015; Japanese Society for Fatigue Science 2011a, 2011b)

At Scr and 12w, the POMS2 Japanese version was used to assess participants' current mood status, which included the measured values and the amount and rate of change from Scr of the total mood disturbance score, tension–anxiety, depression–dejection, anger–hostility, vigor–activity, fatigue–inertia, confusion–bewilderment, friendliness, and each questionnaire item at 12w.

##### 2.4.2.5 Blood Flow Test

At Scr and 12w, the blood flow rate at the fingertips before and after a cold‐water load was calculated using a laser speckle blood flow meter (OMEGAZONE OZ‐2, OMEGAWAVE Inc., Tokyo, Japan). The measured values and the amount and rate of change from Scr of the blood flow, area under the curve (AUC) from immediately after load, and the amount of changes from before and immediately after load to each time point at 12w were evaluated. In each examination, the cold‐water load was performed as described in Appendix S1, and measurements were taken six times as follows: before load, immediately after load, and 5, 10, 20, and 30 min after load. The AUC was calculated immediately after load, and the change was computed based on before and immediately after load findings. The blood flow test was performed on the left‐hand fingers simultaneously as the skin surface temperature test.

##### 2.4.2.6 Skin Surface Temperature Test

The palm surface temperature before and after the cold‐water load was evaluated by thermography (InfReC R450, Nippon Avionics Co. Ltd., Kanagawa, Japan) at Scr and 12w. The measured values and the amount and rate of change from Scr of the palmar surface temperature were evaluated at 12w. In each examination, the cold‐water load was performed as described in Appendix S1, and measurements were taken six times: before load, immediately after load, and 5, 10, 20, and 30 min after load. The AUC was calculated immediately after load, and the change was computed before and immediately after load. The skin surface temperature test was performed on the right‐hand palm simultaneously as the blood flow test.

##### 2.4.2.7 Sleep Test

Sleep quality at bedtime was assessed using InSomnograf (S'UIMIN Inc., Tokyo, Japan) at Scr and 12w. The measured values and the amount and rate of change from Scr of the sleep score, sleep onset, sleep offset, midpoint of sleep, time in bed after sleep offset, sleep‐onset latency, StageR latency, NotScored total time, N1 total time, N2 total time, N3 total time, rapid eye movement (REM) total time, total sleep time (TST), wake time after sleep onset, sleep efficiency (%total recording time [TRT]), percentage of N1 (%TST), percentage of N2 (%TST), percentage of N3 (%TST), and percentage of REM (%TST) were evaluated at 12w. For each examination, measurements were taken for 3 days (1–3 days before the examination), with the participants wearing the device at bedtime. The mean value was calculated from the scores for 2 days before the examination.

##### 2.4.2.8 Blood Test

Blood tests were performed at Scr and 12w. The measured values and the amount and rate of change from Scr of oxidative stress markers 8‐hydroxy‐2′‐deoxyguanosine (8‐OHdG), 2‐thiobarbituric acid reactive substances (TBARS), nitric oxide (NO) metabolic products, and pentosidine were evaluated at 12w. For each examination, approximately 15 mL of venous blood was collected from the participants, and the blood samples were separated into approximately 5 mL of serum and frozen. Assays were performed at Fujicco Co. Ltd.

#### 2.4.3 Safety Evaluation

The safety assessment included physical measurements, urinalysis, and blood tests. Physical measurements included body weight, body mass index (BMI), and systolic and diastolic blood pressure. BMI was calculated using the height measured at Scr.

In the urinalysis, protein, glucose, pH, and occult blood were measured. Each item was measured according to the standard method by LSI Medience Corporation (Tokyo, Japan).

Blood tests included white blood cell count, red blood cell count, hemoglobin levels, hematocrit levels, platelet count, aspartate aminotransferase levels, alanine aminotransferase levels, γ‐glutamyl transpeptidase levels, total bilirubin levels, total protein levels, urea nitrogen levels, creatinine levels, uric acid levels, sodium levels, potassium levels, chloride levels, serum amylase levels, total cholesterol levels, high‐ and low‐density lipoprotein‐cholesterol levels, triglyceride levels, glucose levels, and hemoglobin A1c levels (NGSP). Each item was measured according to the standard method by LSI Medience Corporation.

The number of adverse events was recorded. In such events, the physician immediately provided necessary and appropriate measures and decided whether the participant could continue the study and if the emergency key should be opened. The physician also evaluated and reported the relationship between the adverse event and intervention food.

Interviews were conducted at each assessment point to confirm participants' health status. The participants were asked to record their living conditions daily, including the intake of test foods, changes in physical condition, and use of medications in their diaries.

### 2.5 Sample Size

Because no study has evaluated sleep quality by sleepiness on rising in OSA‐MA after 12w of consumption of BE contained food in humans, we assumed a large difference between the groups (BE‐containing test food and placebo), and *d* = 0.80 based on Cohen's suggestion was used (Cohen 1992). The statistical significance level (α) was set at 0.05, and the statistical power (1 − β) was set at 0.80. A minimum of 52 participants (*n* = 26 in each group) was required. To maximize the statistical power (1 − β) within the budget, the target number of participants was set at 60 (*n* = 30 in each group). Statistical power (1 − β) was recalculated, which was 0.86. In anticipation of dropouts and noncompliance during the study period, the number of participants was set at 64 (32 in each group).

### 2.6 Selection, Randomization, and Blinding

Among the 94 participants who consented to participation, 64 were determined eligible by the physician. The test foods were provided by Fujicco Co. Ltd. to the contract research organization (CRO). The CRO's test food dispatcher confirmed the indistinguishability of the test food, entered and confirmed the screening data, and provided the identification number to the allocation controller, who was not directly involved in the study. Allocation was by stratified block random allocation with allocation adjustment factors of sleepiness on rising in OSA‐MA (above and below the median in Scr data), sex (male and female), and age (≥ 40 and < 40 years), and the allocation controller randomly allocated 32 participants to the BE contained food group (BE group) and the Placebo group according to a computer‐generated randomization digit table. Only the test food dispatcher of the CRO, who received the allocation table with the coded test foods, sent the test foods to each participant according to the allocation table. The allocation controller locked the allocation table until the day of opening. The sponsors, principal investigator, entire CRO staff (i.e., the director of the trial, director of trial conduction, person in charge of monitoring, director and staff of statistical analysis, and person in charge of shipping), medical institution staff, institutional review board members, contract laboratory, and others involved in this study were unaware of the group assignments and were not involved in the allocation.

### 2.7 Statistical Analysis

All statistical analyses were performed using two‐tailed tests, and the significance level was set at 5%. IBM SPSS Statistics for Windows version 23.0 (IBM Japan Ltd., Tokyo, Japan) was used, and other validated statistical software was used as needed. Because the analysis focused on the primary outcome, the multiplicity of occurrence of the secondary outcomes was not considered.

Regarding participants' characteristics, sex was expressed as the number of males and females in the group. Other items were expressed as their means and standard deviations (SDs). Inter‐group comparisons were performed using the chi‐squared test for sex and Welch's *t*‐test for other items.

Data collected from the OSA‐MA and the original questionnaire were presented as means and SDs. The difference between the BE and placebo groups (BE group minus Placebo group; at Scr, mean difference; after the intervention, estimated marginal means [EMM] difference) and its 95% confidence interval (CI) were determined. Scr data were compared between groups using Welch's *t*‐test for baseline data, and postintervention data were compared using a linear mixed model with baseline as a covariate and time, group, time and group interaction, and study participant as factors.

PSQI‐J, POMS2, blood flow test, and skin surface temperature test values were presented as means and SDs. The difference between the BE and placebo groups (BE group minus Placebo group; at Scr, mean difference; after the intervention, EMM difference) and its 95% CI were displayed. Scr data were compared between groups using Welch's *t*‐test for baseline data, and postintervention data were compared by an analysis of covariance with baseline values as the covariate and group as the factor. Each question item of the PSQI‐J and POMS2 was expressed as minimum, median, maximum, quartile range (first quartile, Q1; third quartile, Q3), and rank sum for each group and was compared between groups using the Mann–Whitney *U* test. Subgroup analyses used the same analytical methods as for the overall analysis.

In the safety evaluation, adverse events were presented as the number of participants in the group, incidence rate within the group, difference in the incidence rate, and 95% CI of the incidence rate. The proportion of cases of abnormal urinalysis and blood findings after the intervention was aggregated for each study participant, and the applicability ratio was aggregated by group. The 95% CI of the applicable percentage for each group and the difference in the applicable ratio between groups (BE group minus Placebo group) were calculated. A chi‐squared test was performed to compare the applicability percentage between groups. Furthermore, the study investigator or physician individually confirmed the safety of the evaluation items, that is, no medically problematic changes were caused by test food intake.

The analysis datasets were constructed. The intention‐to‐treat dataset included all study participants. The full analysis set (FAS) included all participants, except those who did not receive the allocated intervention, did not meet the eligibility criteria, never received an intervention after allocation, and had no postallocation data. The per‐protocol analysis set included data from the FAS, also excluding participants who (1) had a test food intake rate of < 80%, (2) exhibited significant behavior that affected the reliability of the test results (e.g., missing diary records), (3) met the exclusion criteria after enrollment, (4) violated the compliance rules during the study period, (5) took foods or medications that could be expected to affect the test results significantly, (6) engaged in activities that were significantly different from their lifestyle upon study enrollment, and (7) had clear reasons for exclusion. The safety analysis population (SAF) included all study participants, except those who met at least one of the following conditions: those who (1) did not receive the allocated intervention, (2) never received the allocated intervention after allocation, or (3) never underwent the allocated safety evaluation.

## 3 Results

### 3.1 Analysis Set

**FIGURE 1:**
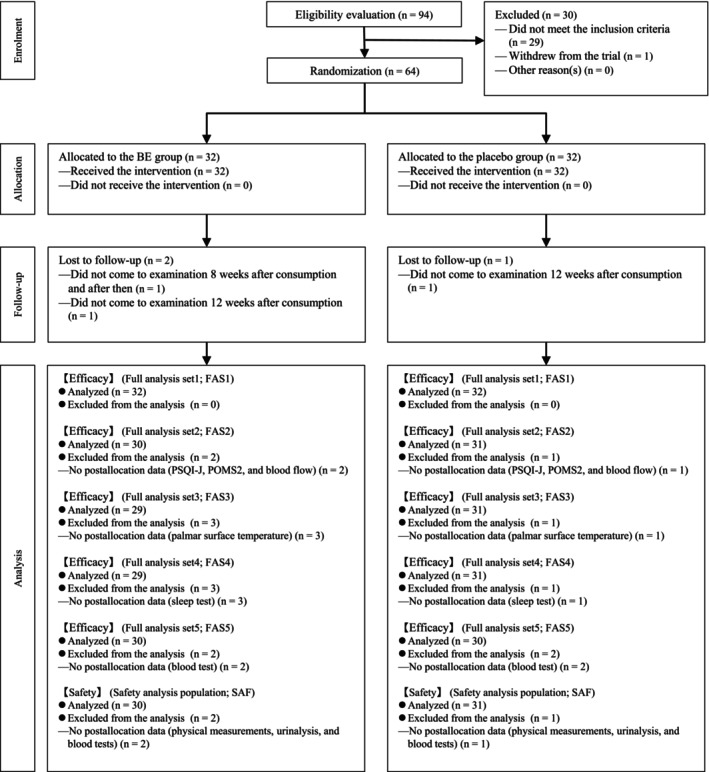
Participant flowchart.

Figure 1 presents the follow‐up flowchart for the study participants. Recruitment was conducted from May 31 to August 3, 2023, and the study period was from August 3 to December 23, 2023. Although all participants received the allocated intervention, one participant in the BE group did not participate in the examinations after 8w, and one participant in each group did not participate in the 12w examination. FAS included the dataset for the analysis of efficacy outcomes, and the actual analysis employed FAS1, which included all participants, and FAS2–FAS5, except for participants who had no data included as analysis variables after the assignment. Moreover, SAF included the dataset for the safety endpoints, and three participants (two in the BE group and one in the Placebo group) who had no postassignment data (physical measurements, urinalysis, and peripheral blood tests) were excluded from the analysis. In each analysis dataset, 64 participants were included in FAS1 (*n* = 32 in each group), 61 in FAS2 and SAF (BE group, *n* = 30; Placebo group, *n* = 31), 60 in FAS3 and FAS4 (BE group, *n* = 29; Placebo group, *n* = 31), and 60 in FAS5 (*n* = 30 in each group). The list of participants who had missing data and the list of participants excluded for each analysis dataset are shown in Appendices S2 and S3, respectively. Participant background is shown in Table 3.

**TABLE 3:** Characteristics of the participants.

Items	ITT, FAS1		FAS2, SAF		FAS3		FAS4		FAS5	
	BE group (*n* = 32)	Placebo group (*n* = 32)	BE group (*n* = 30)	Placebo group (*n* = 31)	BE group (*n* = 29)	Placebo group (*n* = 31)	BE group (*n* = 29)	Placebo group (*n* = 31)	BE group (*n* = 30)	Placebo group (*n* = 30)
Sex, male/female	16/16	16/16	14/16	16/15	13/16	16/15	13/16	16/15	14/16	16/14
Age, years	46.3 (10.9)	45.5 (8.7)	46.0 (11.0)	45.8 (8.7)	45.7 (11.1)	45.8 (8.7)	46.5 (10.9)	45.8 (8.7)	46.0 (11.0)	45.9 (8.8)
Height, cm	167.5 (8.0)	166.8 (8.2)	167.3 (8.2)	166.7 (8.3)	167.1 (8.3)	166.7 (8.3)	167.2 (8.3)	166.7 (8.3)	167.3 (8.2)	166.8 (8.4)
Body weight, kg	62.7 (8.8)	63.4 (13.5)	62.6 (9.1)	63.0 (13.5)	62.4 (9.2)	63.0 (13.5)	62.7 (9.2)	63.0 (13.5)	62.6 (9.1)	63.3 (13.7)
Body mass index, kg/m^2^	22.3 (2.3)	22.6 (3.2)	22.3 (2.4)	22.5 (3.2)	22.3 (2.4)	22.5 (3.2)	22.4 (2.4)	22.5 (3.2)	22.3 (2.4)	22.5 (3.3)
Systolic blood pressure, mmHg	112.8 (12.6)	108.2 (12.6)	113.5 (12.6)	108.4 (12.7)	113.1 (12.6)	108.4 (12.7)	114.2 (12.2)	108.4 (12.7)	113.5 (12.6)	109.0 (12.5)
Diastolic blood pressure, mmHg	72.2 (9.9)	70.2 (9.5)	72.6 (10.0)	70.1 (9.7)	72.2 (10.0)	70.1 (9.7)	73.3 (9.4)	70.1 (9.7)	72.6 (10.0)	70.6 (9.5)
Sleepiness on rising, point	14.2 (4.6)	14.5 (4.9)	13.9 (4.3)	14.8 (4.7)	13.8 (4.3)	14.8 (4.7)	13.9 (4.3)	14.8 (4.7)	13.9 (4.3)	14.9 (4.7)
Initiation and maintenance of sleep, point	13.7 (5.1)	14.9 (4.8)	13.6 (5.2)	14.8 (4.9)	13.6 (5.3)	14.8 (4.9)	13.5 (5.3)	14.8 (4.9)	13.6 (5.2)	14.8 (5.0)
Frequent dreaming, point	21.3 (7.0)	20.6 (7.6)	21.3 (7.2)	20.3 (7.6)	21.2 (7.3)	20.3 (7.6)	21.2 (7.3)	20.3 (7.6)	21.3 (7.2)	20.0 (7.6)
Refreshing, point	13.4 (5.2)	13.8 (4.4)	13.1 (4.9)	14.1 (4.1)	13.1 (5.0)	14.1 (4.1)	13.1 (5.0)	14.1 (4.1)	13.1 (4.9)	14.1 (4.2)
Sleep length, point	15.4 (5.9)	15.0 (3.6)	14.9 (5.4)	14.9 (3.6)	14.8 (5.5)	14.9 (3.6)	15.1 (5.4)	14.9 (3.6)	14.9 (5.4)	15.1 (3.5)

*Note:* Sex is shown by the number of participants in the group and other items are shown by mean, standard deviation.

Abbreviations: BE, black soybean seed coat extract; FAS, full analysis set; ITT, intention to treat; SAF, safety analysis population.

^a^ See Appendix S3 for differences between each FAS.

Multiple subgroup analyses were performed to explore the effect of the intake of BE on sleep quality. Because sleep quality is affected by sex and age (Mander et al. 2017), we constructed subgroups for males, females, and participants aged ≥ 40 years.

Confirming the scores of sleepiness on rising in OSA‐MA based on participants' background, a large variation was noted in FAS1, with minimum and maximum values of 1.8 and 25.5 points, respectively (Table 3) even when this study included individuals who complained of fatigue upon waking and sleep quality (short sleep time, difficulty falling asleep, difficulty sleeping soundly, dreaming, inability to get over fatigue, etc.) in daily life. Considering that the average score of Japanese adults' sleepiness on rising in the OSA‐MA was 17.4 points (Yamamoto et al. 1999), whether the participants were really poor‐quality sleepers remains questionable. Therefore, a subgroup of participants whose sleepiness on rising score in OSA‐MA at Scr was below the median (14.15 points) was constructed, and the effect of the test food on those with poor sleep quality was examined.

### 3.2 OSA‐MA

**FIGURE 2:**
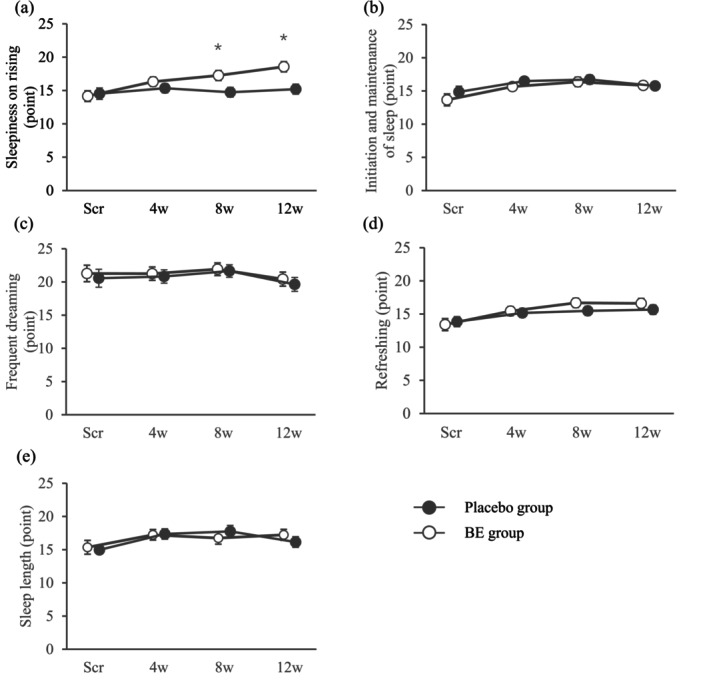
Changes in each factor in the OSA‐MA. Changes in each factor (a, sleepiness on rising; b, initiation and maintenance of sleep; c, frequent dreaming; d, refreshing; e, sleep length) in the OSA‐MA among the full analysis set 1 are shown as means and standard error (SE) at Scr and as estimated marginal mean and SE at 4w and after. BE group (Scr and 4w: *n* = 32, 8w: *n* = 31, 12w: *n* = 30) and placebo group (Scr, 4w, and 8w: *n* = 32, 12w: *n* = 31). OSA‐MA, OSA sleep inventory MA version; Scr, screening (baseline); 4w, 4 weeks after consumption; 8w, 8 weeks after consumption; 12w, 12 weeks after consumption. *p < 0.05.

For the OSA‐MA factor scores in FAS1, the BE group recorded significantly higher measured values and change in sleepiness on rising at 8w and at 12w than the Placebo group (Figure 2a). In the subitem of sleepiness upon waking (questions 2, 4, 8, and 14), although no significant inter‐group difference was confirmed for “Question 8_I feel clearheaded–I feel foggy headed,” the BE group recorded significantly higher measured values and amounts of change at 12w for “Question 2_I am concentrated–I am not concentrated,” at 8w and 12w for “Question 4_I am relaxed–I am stressed,” and at 12w for “Question 14_I can answer a survey quickly and easily right now–It's troublesome to answer” than the Placebo group (Table 4). No other items were affected by the intervention of BE (data not shown).

**TABLE 4:** Comparison of OSA‐MA (each item; FAS1).

Analysis set	Items	Subitems	Unit	Classification	Time point	BE group					Placebo group					Group comparison		
						*n*	Mean	SD	EMM	95% CI	*n*	Mean	SD	EMM	95% CI	Δ	95% CI	*p*
FAS1	Sleepiness on rising	Question 2_I am concentrated–I am not concentrated	—	Measured value	Scr	32	15.7	5.8	—	—	32	15.8	5.7	—	—	−0.1	−3.0, 2.8	0.931
					4w	32	17.8	5.7	17.7	16.3, 19.2	32	17.4	5.2	17.3	15.9, 18.8	0.4	−1.7, 2.4	0.704
					8w	31	18.8	5.0	19.1	17.6, 20.7	32	17.2	5.5	17.0	15.5, 18.5	2.1	0.0, 4.2	0.054
					12w	30	20.3	6.5	20.8	18.8, 22.8	31	17.6	6.1	17.3	15.3, 19.3	3.5	0.6, 6.3	0.017*
				Amount of change	4w	32	2.0	4.8	2.1	0.6, 3.5	32	1.6	4.3	1.7	0.2, 3.1	0.4	−1.7, 2.4	0.704
					8w	31	3.5	5.1	3.5	2.0, 5.0	32	1.3	4.5	1.4	−0.1, 2.9	2.1	0.0, 4.2	0.054
					12w	30	5.2	6.6	5.1	3.1, 7.1	31	1.4	5.1	1.6	−0.3, 3.6	3.5	0.6, 6.3	0.017*
			%	Rate of change	4w	32	22.6	49.0	22.9	9.6, 36.2	32	19.3	42.7	20.1	6.8, 33.4	2.7	−16.1, 21.5	0.773
					8w	31	37.5	72.9	36.7	18.3, 55.1	32	17.4	47.0	18.4	0.2, 36.7	18.3	−7.7, 44.2	0.164
					12w	30	47.9	71.3	46.2	26.9, 65.5	31	13.2	38.1	20.7	1.5, 39.8	25.5	−1.7, 52.8	0.065
		Question 4_I am relaxed–I am stressed	—	Measured value	Scr	32	13.6	5.3	—	—	32	14.3	5.5	—	—	−0.7	−3.4, 2.0	0.598
					4w	32	16.1	6.1	16.2	14.3, 18.1	32	15.2	5.5	15.1	13.1, 17.0	1.2	−1.6, 3.9	0.395
					8w	31	17.5	5.1	17.9	16.0, 19.8	32	15.3	6.0	15.1	13.3, 17.0	2.7	0.0, 5.4	0.047*
					12w	30	18.5	6.4	18.9	17.0, 20.9	31	15.5	5.7	14.8	12.9, 16.8	4.1	1.3, 6.9	0.004*
				Amount of change	4w	32	2.4	4.6	2.2	0.3, 4.1	32	0.8	7.6	1.0	−0.9, 3.0	1.2	−1.6, 3.9	0.395
					8w	31	4.2	5.2	3.8	1.9, 5.7	32	0.9	7.3	1.1	−0.8, 3.0	2.7	0.0, 5.4	0.047*
					12w	30	5.0	5.2	4.9	2.9, 6.9	31	0.7	6.4	0.8	−1.2, 2.7	4.1	1.3, 6.9	0.004*
			%	Rate of change	4w	31	22.0	48.1	20.2	4.8, 35.6	31	16.4	51.8	18.7	3.3, 34.1	1.6	−20.2, 23.4	0.887
					8w	30	35.9	52.0	32.9	17.4, 48.4	31	17.0	52.5	19.6	4.2, 34.9	13.3	−8.6, 35.2	0.228
					12w	29	39.9	50.8	40.1	24.3, 55.8	31	12.4	45.2	14.3	−1.2, 29.8	25.8	3.7, 47.9	0.023*
		Question 8_I feel clearheaded–I feel foggy headed	—	Measured value	Scr	32	13.0	5.3	—	—	32	12.6	6.2	—	—	0.4	−2.5, 3.3	0.796
					4w	32	15.3	5.9	15.2	13.4, 17.0	32	14.9	5.9	15.0	13.2, 16.7	0.2	−2.3, 2.7	0.872
					8w	31	15.1	4.3	15.4	13.6, 17.1	32	13.1	6.9	13.2	11.5, 14.9	2.2	−0.3, 4.6	0.079
					12w	30	16.2	5.8	16.2	14.6, 17.9	31	14.6	5.7	14.4	12.8, 16.0	1.9	−0.4, 4.2	0.104
				Amount of change	4w	32	2.3	5.3	2.4	0.6, 4.2	32	2.3	6.0	2.2	0.4, 4.0	0.2	−2.3, 2.7	0.872
					8w	31	2.5	4.7	2.6	0.9, 4.3	32	0.5	5.9	0.4	−1.3, 2.1	2.2	−0.3, 4.6	0.079
					12w	30	3.4	4.7	3.5	1.9, 5.1	31	1.6	4.9	1.6	0.0, 3.2	1.9	−0.4, 4.2	0.104
			%	Rate of change	4w	31	27.3	56.4	29.3	12.8, 45.8	31	36.1	62.0	35.3	18.8, 51.9	−6.0	−29.4, 17.3	0.607
					8w	30	23.9	38.4	25.3	7.4, 43.1	31	13.9	65.5	13.5	−4.2, 31.1	11.8	−13.3, 36.9	0.350
					12w	29	27.9	44.7	29.7	12.5, 46.9	31	25.3	57.9	24.9	8.0, 41.8	4.9	−19.3, 29.0	0.689
		Question 14_I can answer a survey quickly and easily right now—It's troublesome to answer	—	Measured value	Scr	32	14.2	4.9	—	—	32	15.3	6.9	—	—	−1.1	−4.1, 1.9	0.481
					4w	32	15.7	5.7	16.0	14.4, 17.6	32	14.4	5.7	14.0	12.4, 15.6	2.0	−0.2, 4.2	0.079
					8w	31	15.5	5.5	16.2	14.2, 18.2	32	14.0	7.2	13.7	11.7, 15.6	2.5	−0.3, 5.3	0.080
					12w	30	17.1	6.6	17.8	15.9, 19.7	31	15.1	5.5	14.6	12.7, 16.5	3.2	0.5, 5.9	0.021*
				Amount of change	4w	32	1.5	4.9	1.4	−0.2, 2.9	32	−0.9	5.1	−0.6	−2.2, 0.9	2.0	−0.2, 4.2	0.079
					8w	31	1.6	5.7	1.5	−0.5, 3.5	32	−1.2	6.3	−1.0	−2.9, 1.0	2.5	−0.3, 5.3	0.080
					12w	30	3.2	6.4	3.2	1.3, 5.1	31	−0.1	5.6	0.0	−1.9, 1.9	3.2	0.5, 5.9	0.021*
			%	Rate of change	4w	32	18.5	53.6	16.7	2.0, 31.4	32	6.0	41.9	8.6	−6.1, 23.3	8.1	−12.7, 29.0	0.439
					8w	31	25.3	79.5	24.4	2.1, 46.7	32	−5.3	46.7	−3.2	−25.3, 18.8	27.6	−3.8, 59.1	0.084
					12w	30	35.0	77.6	33.7	13.4, 54.1	31	13.2	50.0	14.5	−5.7, 34.8	19.2	−9.6, 48.0	0.187
	Initiation and maintenance of sleep	Question 3_I slept well—I didn't sleep well	—	Measured value	Scr	32	13.3	6.3	—	—	32	13.6	5.8	—	—	−0.3	−3.4, 2.7	0.821
					4w	32	14.2	4.5	14.2	12.8, 15.6	32	15.4	4.4	15.3	13.9, 16.7	−1.1	−3.1, 0.9	0.263
					8w	31	15.6	4.9	15.8	14.2, 17.5	32	16.3	4.7	16.2	14.6, 17.8	−0.4	−2.7, 2.0	0.758
					12w	30	14.6	5.1	14.8	13.3, 16.2	31	15.0	4.5	14.8	13.4, 16.2	−0.1	−2.1, 2.0	0.960
				Amount of change	4w	32	0.9	4.8	0.8	−0.6, 2.2	32	1.8	6.4	1.9	0.5, 3.4	−1.1	−3.1, 0.9	0.263
					8w	31	2.6	6.4	2.4	0.8, 4.1	32	2.6	6.6	2.8	1.2, 4.4	−0.4	−2.7, 2.0	0.758
					12w	30	1.4	5.1	1.4	−0.1, 2.8	31	1.3	5.2	1.4	0.0, 2.8	−0.1	−2.1, 2.0	0.960
			%	Rate of change	4w	30	7.3	30.4	10.0	−11.9, 32.0	32	43.4	101.3	41.6	20.4, 62.8	−31.6	−62.1, −1.0	0.043*
					8w	29	23.6	49.3	25.6	4.3, 46.9	32	50.0	98.7	48.0	27.5, 68.4	−22.3	−51.9, 7.2	0.136
					12w	28	14.7	38.9	16.7	3.1, 30.4	31	29.0	59.7	27.2	14.2, 40.3	−10.5	−29.4, 8.4	0.270
		Question 7_I often dozed off until I fell asleep–I was less likely to doze off until I fell asleep	—	Measured value	Scr	32	14.4	6.9	—	—	32	15.9	6.7	—	—	−1.5	−4.9, 1.9	0.392
					4w	32	18.0	4.8	18.2	16.5, 19.9	32	18.3	5.3	18.1	16.4, 19.8	0.1	−2.3, 2.5	0.936
					8w	31	18.2	5.2	18.4	16.6, 20.3	32	18.3	5.3	18.1	16.3, 20.0	0.3	−2.3, 2.9	0.822
					12w	30	17.6	6.4	17.9	15.8, 20.0	31	16.6	6.0	16.4	14.3, 18.4	1.5	−1.4, 4.5	0.310
				Amount of change	4w	32	3.6	7.5	3.0	1.3, 4.7	32	2.3	6.6	2.9	1.2, 4.6	0.1	−2.3, 2.5	0.936
					8w	31	3.8	8.2	3.3	1.4, 5.1	32	2.4	6.7	3.0	1.2, 4.8	0.3	−2.3, 2.9	0.822
					12w	30	3.2	7.6	2.7	0.6, 4.8	31	0.7	6.8	1.2	−0.8, 3.3	1.5	−1.4, 4.5	0.310
			%	Rate of change	4w	30	33.2	58.1	29.7	15.2, 44.2	31	26.2	55.5	29.7	15.4, 43.9	0.1	−20.3, 20.4	0.994
					8w	29	36.0	69.0	31.9	11.9, 52.0	31	28.8	74.9	32.7	13.2, 52.2	−0.8	−28.8, 27.3	0.957
					12w	28	40.7	76.4	35.5	9.4, 61.5	30	21.4	95.5	24.7	−0.7, 50.0	10.8	−25.6, 47.2	0.555
		Question 10_I fell asleep right away–I didn't sleep right away	—	Measured value	Scr	32	12.3	6.5	—	—	32	12.8	4.1	—	—	−0.4	−3.2, 2.3	0.750
					4w	32	13.8	5.3	13.9	12.3, 15.5	32	14.6	4.7	14.5	12.9, 16.1	−0.6	−2.9, 1.7	0.594
					8w	31	14.0	4.8	14.1	12.5, 15.7	32	15.0	4.5	14.9	13.3, 16.5	−0.8	−3.0, 1.4	0.472
					12w	30	13.3	4.4	13.3	11.9, 14.8	31	13.3	4.2	13.1	11.7, 14.6	0.2	−1.8, 2.2	0.842
				Amount of change	4w	32	1.5	6.4	1.4	−0.2, 3.0	32	1.8	4.8	2.0	0.4, 3.6	−0.6	−2.9, 1.7	0.594
					8w	31	1.9	7.1	1.6	0.0, 3.2	32	2.2	4.5	2.4	0.8, 4.0	−0.8	−3.0, 1.4	0.472
					12w	30	1.1	6.5	0.8	−0.6, 2.3	31	0.6	4.2	0.6	−0.8, 2.1	0.2	−1.8, 2.2	0.842
			%	Rate of change	4w	30	26.1	93.3	28.4	3.5, 53.4	32	28.0	67.9	27.0	2.8, 51.1	1.5	−33.2, 36.2	0.932
					8w	29	33.1	127.7	33.9	2.0, 65.8	32	30.5	67.4	29.3	−1.4, 60.0	4.6	−39.7, 48.9	0.837
					12w	28	22.1	72.0	22.5	3.5, 41.6	31	14.0	53.6	11.9	−6.4, 30.1	10.7	−15.7, 37.0	0.421
		Question 13_I woke up frequently during sleep–I didn't wake up during sleep	—	Measured value	Scr	32	15.2	6.8	—	—	32	16.9	6.8	—	—	−1.8	−5.2, 1.7	0.309
					4w	32	16.3	6.7	16.8	15.0, 18.6	32	18.8	5.5	18.4	16.6, 20.2	−1.6	−4.2, 0.9	0.213
					8w	31	17.6	6.7	18.0	16.1, 20.0	32	18.1	6.0	17.7	15.7, 19.6	0.4	−2.4, 3.2	0.785
					12w	30	16.6	6.1	17.0	15.4, 18.7	31	17.9	5.4	17.5	15.9, 19.1	−0.5	−2.8, 1.8	0.682
				Amount of change	4w	32	1.1	4.8	0.7	−1.1, 2.5	32	1.9	7.1	2.3	0.5, 4.1	−1.6	−4.2, 0.9	0.213
					8w	31	2.4	6.2	2.0	0.0, 3.9	32	1.1	6.8	1.6	−0.4, 3.5	0.4	−2.4, 3.2	0.785
					12w	30	1.3	5.5	0.9	−0.7, 2.6	31	1.1	5.7	1.4	−0.2, 3.0	−0.5	−2.8, 1.8	0.682
			%	Rate of change	4w	31	15.6	52.0	11.5	−3.8, 26.8	31	19.0	48.1	22.8	7.6, 38.1	−11.3	−33.0, 10.4	0.300
					8w	30	24.2	55.5	21.1	6.2, 35.9	31	10.7	38.1	14.1	−0.6, 28.8	6.9	−14.1, 28.0	0.512
					12w	29	19.9	58.2	16.6	1.8, 31.4	30	12.1	35.1	14.7	0.1, 29.4	1.8	−19.1, 22.7	0.862
		Question 16_My sleep was light—My sleep was deep	—	Measured value	Scr	32	13.0	7.8	—	—	32	15.0	6.8	—	—	−2.0	−5.6, 1.6	0.277
					4w	32	14.8	5.5	15.1	13.6, 16.5	32	16.8	4.0	16.4	15.0, 17.9	−1.3	−3.4, 0.7	0.207
					8w	31	15.1	5.6	15.4	13.6, 17.3	32	17.0	5.1	16.7	14.9, 18.6	−1.3	−3.9, 1.3	0.314
					12w	30	15.5	4.7	15.9	14.3, 17.4	31	17.7	5.3	17.3	15.8, 18.8	−1.4	−3.6, 0.8	0.204
				Amount of change	4w	32	1.7	5.2	1.1	−0.3, 2.6	32	1.8	7.1	2.4	1.0, 3.9	−1.3	−3.4, 0.7	0.207
					8w	31	2.3	7.2	1.4	−0.4, 3.3	32	1.9	8.1	2.7	0.9, 4.6	−1.3	−3.9, 1.3	0.314
					12w	30	2.7	6.1	1.9	0.3, 3.4	31	2.6	6.5	3.3	1.8, 4.8	−1.4	−3.6, 0.8	0.204
			%	Rate of change	4w	28	21.1	71.5	19.2	1.0, 37.3	31	25.2	50.7	27.1	9.9, 44.4	−8.0	−33.0, 17.1	0.527
					8w	27	19.1	51.3	16.3	−1.3, 33.9	31	29.0	69.1	31.0	14.5, 47.5	−14.7	−38.9, 9.4	0.228
					12w	26	33.9	88.0	29.3	7.7, 50.9	30	32.4	55.2	33.8	13.4, 54.2	−4.5	−34.2, 25.2	0.761
	Frequent dreaming	Question 9_I had many nightmares—I didn't have nightmares	—	Measured value	Scr	32	23.7	5.9	—	—	32	21.6	8.0	—	—	2.1	−1.4, 5.6	0.232
					4w	32	23.1	7.0	22.6	20.4, 24.7	32	21.6	6.4	22.1	20.0, 24.2	0.5	−2.5, 3.5	0.751
					8w	31	23.7	6.2	23.0	21.0, 25.1	32	22.6	6.2	23.0	21.0, 25.0	0.1	−2.8, 2.9	0.971
					12w	30	22.7	7.3	22.0	19.6, 24.5	31	20.7	6.9	20.9	18.5, 23.3	1.1	−2.3, 4.6	0.516
				Amount of change	4w	32	−0.7	5.6	−0.1	−2.2, 2.1	32	0.0	8.4	−0.5	−2.7, 1.6	0.5	−2.5, 3.5	0.751
					8w	31	−0.2	6.0	0.4	−1.6, 2.4	32	1.0	8.0	0.4	−1.7, 2.4	0.1	−2.8, 2.9	0.971
					12w	30	−1.1	6.7	−0.6	−3.0, 1.8	31	−0.7	9.2	−1.7	−4.1, 0.7	1.1	−2.3, 4.6	0.516
			%	Rate of change	4w	32	−0.9	28.0	4.8	−15.9, 25.5	32	21.3	92.7	15.8	−4.9, 36.5	−11.0	−40.4, 18.4	0.456
					8w	31	3.4	31.3	9.5	−9.2, 28.2	32	27.4	89.6	21.0	2.5, 39.5	−11.5	−38.0, 15.0	0.389
					12w	30	−1.2	36.7	3.8	−18.1, 25.7	31	20.1	98.2	11.7	−10.1, 33.5	−7.9	−38.9, 23.2	0.615
		Question 12_I dreamed often—I didn't dream	—	Measured value	Scr	32	18.8	8.7	—	—	32	19.5	8.2	—	—	−0.7	−4.9, 3.5	0.746
					4w	32	19.8	8.7	20.0	17.7, 22.2	32	19.7	6.6	19.5	17.2, 21.8	0.4	−2.8, 3.7	0.783
					8w	31	20.8	7.3	20.8	18.6, 22.9	32	20.5	6.2	20.3	18.2, 22.5	0.5	−2.6, 3.5	0.766
					12w	30	19.0	7.3	18.9	16.5, 21.2	31	18.6	7.5	18.3	16.0, 20.6	0.5	−2.8, 3.8	0.752
				Amount of change	4w	32	1.0	6.3	0.8	−1.5, 3.1	32	0.2	8.7	0.4	−1.9, 2.7	0.4	−2.8, 3.7	0.783
					8w	31	1.9	8.4	1.6	−0.5, 3.8	32	0.9	7.8	1.2	−1.0, 3.3	0.5	−2.6, 3.5	0.766
					12w	30	0.3	8.2	−0.3	−2.6, 2.1	31	−0.7	7.9	−0.8	−3.1, 1.5	0.5	−2.8, 3.8	0.752
			%	Rate of change	4w	29	6.3	32.4	7.5	−6.3, 21.2	31	11.0	52.2	10.0	−3.3, 23.3	−2.6	−21.7, 16.6	0.789
					8w	28	12.2	44.8	13.3	−0.2, 26.7	31	14.1	48.9	12.9	0.0, 25.7	0.4	−18.2, 19.0	0.965
					12w	27	−0.4	30.7	−1.3	−15.2, 12.7	30	1.8	48.6	−0.3	−13.7, 13.0	−0.9	−20.3, 18.4	0.923
	Refreshing	Question 1_I'm still tired—I'm not tired	—	Measured value	Scr	32	12.8	6.4	—	—	32	13.8	4.9	—	—	−1.0	−3.9, 1.8	0.470
					4w	32	15.8	4.5	15.9	14.2, 17.6	32	15.3	5.6	15.1	13.4, 16.8	0.8	−1.6, 3.2	0.507
					8w	31	17.0	4.7	17.2	15.5, 18.9	32	15.7	5.0	15.5	13.9, 17.2	1.6	−0.7, 4.0	0.170
					12w	30	17.3	6.7	17.8	16.0, 19.6	31	16.5	5.8	15.9	14.1, 17.7	1.9	−0.7, 4.5	0.152
				Amount of change	4w	32	3.0	6.0	2.6	0.9, 4.4	32	1.4	6.6	1.8	0.1, 3.6	0.8	−1.6, 3.2	0.507
					8w	31	4.6	6.1	3.9	2.2, 5.6	32	1.8	6.1	2.3	0.6, 3.9	1.6	−0.7, 4.0	0.170
					12w	30	4.9	5.2	4.5	2.7, 6.4	31	2.4	5.4	2.7	0.8, 4.5	1.9	−0.7, 4.5	0.152
			%	Rate of change	4w	29	26.9	52.8	28.9	10.2, 47.5	32	28.3	77.4	27.6	9.8, 45.3	1.3	−24.5, 27.1	0.920
					8w	28	47.9	80.6	46.8	25.3, 68.3	32	32.4	80.8	31.5	11.2, 51.8	15.3	−14.3, 44.9	0.305
					12w	27	40.9	52.4	40.5	19.7, 61.2	31	25.4	64.1	29.1	9.6, 48.6	11.3	−17.2, 39.9	0.429
		Question 5_I feel sluggishness in the body—I feel nimbleness in the body	—	Measured value	Scr	32	12.1	4.8	—	—	32	11.8	4.1	—	—	0.3	−2.0, 2.5	0.824
					4w	32	13.6	4.4	13.4	11.9, 15.0	32	13.8	5.4	13.9	12.4, 15.4	−0.4	−2.6, 1.7	0.692
					8w	31	14.8	4.7	14.9	13.3, 16.5	32	14.1	5.5	14.1	12.5, 15.7	0.8	−1.5, 3.1	0.474
					12w	30	14.3	4.6	14.4	12.9, 16.0	31	14.9	5.3	14.7	13.2, 16.2	−0.3	−2.5, 1.9	0.808
				Amount of change	4w	32	1.5	4.6	1.6	0.1, 3.1	32	2.0	4.7	2.0	0.5, 3.5	−0.4	−2.6, 1.7	0.692
					8w	31	3.2	4.2	3.0	1.4, 4.6	32	2.2	5.4	2.2	0.6, 3.8	0.8	−1.5, 3.1	0.474
					12w	30	2.7	3.6	2.5	1.0, 4.1	31	2.6	5.3	2.8	1.3, 4.3	−0.3	−2.5, 1.9	0.808
			%	Rate of change	4w	31	19.3	53.0	20.5	4.7, 36.3	31	17.0	40.7	17.0	1.2, 32.8	3.5	−18.8, 25.9	0.754
					8w	30	36.1	48.1	35.1	17.6, 52.7	31	22.2	60.5	22.3	5.0, 39.6	12.8	−11.8, 37.5	0.302
					12w	29	24.5	37.7	23.9	9.3, 38.5	31	27.6	47.5	27.6	13.3, 41.9	−3.7	−24.1, 16.7	0.719
		Question 11_I feel uncomfortable—I feel comfortable	—	Measured value	Scr	32	15.3	5.7	—	—	32	15.8	5.9	—	—	−0.6	−3.5, 2.3	0.699
					4w	32	16.8	6.1	16.8	15.1, 18.5	32	16.8	4.0	16.6	15.0, 18.3	0.2	−2.2, 2.6	0.874
					8w	31	17.6	5.1	17.8	16.2, 19.5	32	17.0	4.9	16.9	15.3, 18.6	0.9	−1.5, 3.3	0.448
					12w	30	17.4	5.8	17.6	15.7, 19.4	31	16.8	5.4	16.5	14.7, 18.3	1.1	−1.5, 3.6	0.395
				Amount of change	4w	32	1.5	5.8	1.3	−0.4, 3.0	32	0.9	6.4	1.1	−0.6, 2.8	0.2	−2.2, 2.6	0.874
					8w	31	2.6	5.6	2.3	0.6, 4.0	32	1.2	6.5	1.4	−0.3, 3.0	0.9	−1.5, 3.3	0.448
					12w	30	2.2	4.8	2.0	0.2, 3.8	31	0.8	6.8	0.9	−0.9, 2.7	1.1	−1.5, 3.6	0.395
			%	Rate of change	4w	31	10.8	37.5	10.8	−1.0, 22.5	32	16.8	40.7	17.0	5.4, 28.6	−6.2	−22.7, 10.3	0.455
					8w	30	19.6	40.5	18.7	5.2, 32.2	32	18.9	49.3	19.1	6.1, 32.2	−0.4	−19.1, 18.3	0.966
					12w	29	13.8	38.9	13.9	−1.4, 29.2	31	15.9	51.6	15.6	0.7, 30.4	−1.6	−23.0, 19.7	0.880
	Sleep length	Question 6_I have an appetite—I have no appetite	—	Measured value	Scr	32	17.2	8.5	—	—	32	15.7	7.3	—	—	1.5	−2.4, 5.5	0.440
					4w	32	19.5	8.2	18.9	16.7, 21.1	32	17.9	6.8	18.1	15.9, 20.4	0.8	−2.4, 3.9	0.629
					8w	31	16.8	8.5	16.7	14.4, 19.1	32	17.8	6.4	18.1	15.8, 20.4	−1.4	−4.7, 1.9	0.408
					12w	30	18.1	8.3	17.9	15.7, 20.1	31	16.8	6.3	17.3	15.1, 19.4	0.7	−2.4, 3.8	0.668
				Amount of change	4w	32	2.3	6.4	2.7	0.5, 5.0	32	2.2	8.0	2.0	−0.3, 4.2	0.8	−2.4, 3.9	0.629
					8w	31	0.1	6.7	0.5	−1.8, 2.9	32	2.2	8.5	1.9	−0.4, 4.2	−1.4	−4.7, 1.9	0.408
					12w	30	1.5	6.5	1.7	−0.4, 3.9	31	1.5	7.4	1.1	−1.1, 3.2	0.7	−2.4, 3.8	0.668
			%	Rate of change	4w	32	27.9	57.7	33.7	6.0, 61.4	32	41.2	112.4	38.3	10.7, 65.9	−4.6	−43.8, 34.6	0.815
					8w	31	9.3	53.7	13.8	−15.8, 43.4	32	44.1	118.7	41.5	12.1, 70.9	−27.7	−69.5, 14.1	0.190
					12w	30	21.0	61.5	25.0	−9.8, 59.8	31	38.7	137.2	34.0	−0.6, 68.6	−8.9	−58.1, 40.2	0.717
		Question 15_Sleeping time was long—Sleeping time was short	—	Measured value	Scr	32	13.5	6.9	—	—	32	14.3	4.5	—	—	−0.8	−3.7, 2.2	0.611
					4w	32	15.4	5.7	15.4	13.6, 17.2	32	16.8	4.6	16.7	14.9, 18.5	−1.3	−3.8, 1.3	0.315
					8w	31	16.3	5.9	16.6	14.4, 18.9	32	17.6	6.6	17.5	15.3, 19.8	−0.9	−4.1, 2.3	0.566
					12w	30	16.1	5.5	16.4	14.5, 18.3	31	15.6	5.1	15.2	13.3, 17.1	1.2	−1.5, 4.0	0.372
				Amount of change	4w	32	1.9	7.6	1.6	−0.2, 3.4	32	2.6	6.2	2.9	1.1, 4.7	−1.3	−3.8, 1.3	0.315
					8w	31	3.2	8.9	2.8	0.5, 5.1	32	3.4	6.8	3.7	1.5, 6.0	−0.9	−4.1, 2.3	0.566
					12w	30	2.9	7.7	2.6	0.7, 4.5	31	1.1	5.8	1.4	−0.6, 3.3	1.2	−1.5, 4.0	0.372
			%	Rate of change	4w	28	7.1	48.8	11.4	−4.0, 26.8	32	29.9	51.8	26.5	12.2, 40.9	−15.1	−36.2, 6.0	0.157
					8w	27	10.7	46.4	12.9	−6.8, 32.6	32	29.2	59.6	27.2	9.0, 45.4	−14.4	−41.2, 12.5	0.289
					12w	26	14.4	47.7	18.3	2.3, 34.3	31	13.5	44.2	10.3	−4.4, 25.0	8.0	−13.8, 29.7	0.466

Abbreviations: 12w, 12 weeks after consumption; 4w, 4 weeks after consumption; 8w, 8 weeks after consumption; 95% CI, 95% confidence interval; Amount of change, amount of change from Scr; EMM, estimated marginal mean; FAS1, full analysis set1; OSA‐MA, OSA sleep inventory MA version; Rate of change, rate of change from Scr; Scr, screening (before consumption); SD, standard deviation; Δ, Difference between groups (BE group–placebo group).

* *p* < 0.05.

The results for each subgroup for sleepiness on rising and its subitems were analyzed, and they showed significant inter‐group differences after the intervention in FAS1.

**FIGURE 3:**
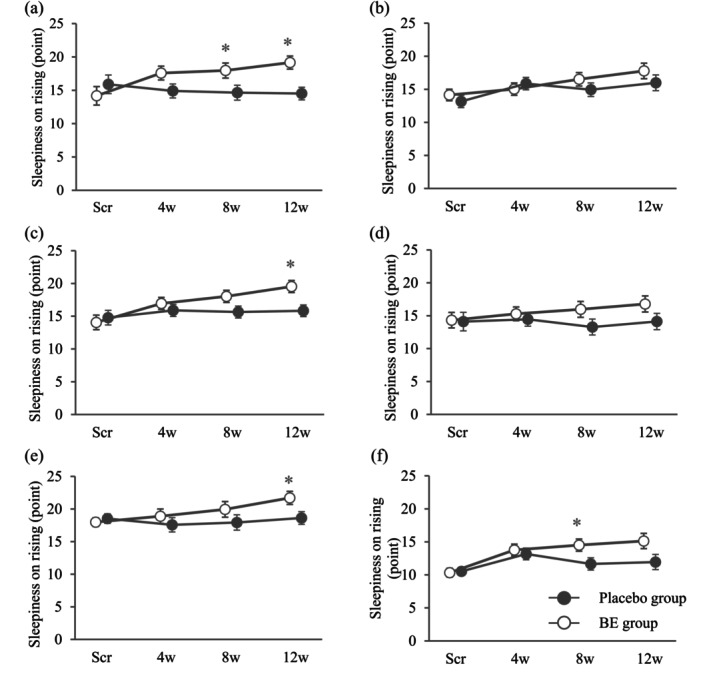
Changes in sleepiness on rising in the OSA‐MA in each subgroup. Changes in sleepiness on rising in the OSA‐MA in each subgroup (a, males of FAS1; b, females of FAS1; c, participants aged ≥ 40 years in FAS1; d, participants aged < 40 years in FAS1; e, participants whose score in sleepiness on rising in OSA‐MA was above or equal to the median (14.15 point) at Scr in FAS4; f, participants whose score on sleepiness on rising in OSA‐MA was below the median (14.15 point) at Scr in FAS4) are shown as mean and standard error (SE) at Scr and as estimated marginal mean and SE at 4w and after. (a) BE group (Scr and 4w: *n* = 16, 8w: *n* = 15, 12w: *n* = 14) and placebo group (Scr, 4w, 8w, and 12w: *n* = 16), (b) BE group (Scr, 4w, 8w, and 12w: *n* = 16) and placebo group (Scr, 4w, and 8w: *n* = 16, 12w: *n* = 15), (c) BE group (Scr and 4w: *n* = 20, 8w: *n* = 19, and 12w: *n* = 18) and placebo group (Scr, 4w, 8w, and 12w: *n* = 20), (d) BE group (Scr, 4w, 8w, and 12w: *n* = 12) and placebo group (Scr, 4w, and 8w: *n* = 12, 12w: *n* = 11), (e) BE group (Scr and 4w: *n* = 16, 8w and 12w: *n* = 15) and placebo group (Scr, 4w, 8w, and 12w: *n* = 16), (f) BE group (Scr, 4w, and 8w: *n* = 16, 12w: *n* = 15) and placebo group (Scr, 4w, and 8w: *n* = 16, 12w: *n* = 15). OSA‐MA, OSA sleep inventory MA version; FAS1, full analysis set1; FAS4, full analysis set4; Scr, screening (baseline); 4w, four weeks after consumption; 8w, eight weeks after consumption; 12w, 12 weeks after consumption. *p < 0.05.

In males of FAS1, the BE group had significantly higher measured value and amount of change in sleepiness on rising at 8w and at 12w than the placebo group (Figure 3a). In the sleepiness on rising subitem, the BE group recorded significantly higher measured value and amount and rate of change at 12w for “Question 2_I am concentrated–I am not concentrated,” at 8w and 12w for “Question 8_I feel clearheaded–I feel foggy headed,” and at 4w, 8w, and 12w for “Question 14_I can answer a survey quickly and easily right now–It's troublesome to answer” than the Placebo group (Table 5). In female participants of FAS1, although there was no significant difference in sleepiness on rising between groups (Figure 3b), among the subitems, the measured value and amount of change at 8w and 12w of “Question 4_I am relaxed–I am stressed” were significantly higher in the BE group than in the Placebo group (Table 5).

**TABLE 5:** Each item of sleepiness on rising in the OSA‐MA (each subgroup analysis).

Analysis set	Items	Unit	Classification	Time point	BE group					Placebo group					Group comparison		
					*n*	Mean	SD	EMM	95% CI	*n*	Mean	SD	EMM	95% CI	Δ	95% CI	*p*
Males of FAS1	Question 2_I am concentrated—I am not concentrated	—	Measured value	Scr	16	16.0	6.8	—	—	16	17.4	6.1	—	—	−1.4	−6.1, 3.2	0.533
				4w	16	18.1	6.3	18.4	16.1, 20.6	16	17.3	5.6	16.6	14.3, 18.9	1.8	−1.4, 5.0	0.267
				8w	15	18.1	5.5	19.3	16.9, 21.8	16	17.8	5.8	17.1	14.7, 19.5	2.3	−1.2, 5.7	0.189
				12w	14	20.1	6.8	21.5	18.6, 24.3	16	16.4	6.2	15.6	12.8, 18.4	5.9	1.8, 9.9	0.006*
			Amount of change	4w	16	2.1	5.1	2.0	−0.3, 4.3	16	−0.2	4.8	0.2	−2.1, 2.5	1.8	−1.4, 5.0	0.267
				8w	15	3.1	5.4	2.9	0.5, 5.4	16	0.3	4.8	0.7	−1.7, 3.1	2.3	−1.2, 5.7	0.189
				12w	14	5.4	6.7	5.1	2.2, 8.0	16	−1.1	4.4	−0.8	−3.6, 2.0	5.9	1.8, 9.9	0.006*
		%	Rate of change	4w	16	28.4	60.2	26.7	7.1, 46.4	16	4.5	28.5	9.1	−10.7, 28.9	17.6	−10.3, 45.6	0.206
				8w	15	42.6	98.6	39.7	5.6, 73.9	16	6.9	36.6	12.4	−21.3, 46.2	27.3	−20.9, 75.6	0.256
				12w	14	57.0	84.2	52.6	23.6, 81.6	16	−4.4	28.8	−0.2	−28.8, 28.4	52.8	11.9, 93.8	0.013*
	Question 4_I am relaxed—I am stressed	—	Measured value	Scr	16	14.3	6.3	—	—	16	15.4	6.0	—	—	−1.1	−5.6, 3.3	0.609
				4w	16	17.1	6.2	17.3	14.5, 20.1	16	15.3	5.1	15.1	12.3, 17.9	2.1	−1.8, 6.1	0.278
				8w	15	17.5	5.8	17.9	15.2, 20.6	15	16.6	4.6	16.5	13.8, 19.2	1.4	−2.5, 5.2	0.465
				12w	14	18.1	7.5	18.7	15.8, 21.6	14	15.7	5.7	15.3	12.4, 18.1	3.4	−0.7, 7.5	0.098
			Amount of change	4w	16	2.8	3.5	2.5	−0.3, 5.3	16	−0.1	9.1	0.3	−2.5, 3.1	2.1	−1.8, 6.1	0.278
				8w	15	3.7	5.7	3.1	0.4, 5.8	15	1.2	8.5	1.7	−1.0, 4.4	1.4	−2.5, 5.2	0.465
				12w	14	3.9	5.4	3.9	1.0, 6.8	14	0.3	6.3	0.5	−2.4, 3.3	3.4	−0.7, 7.5	0.098
		%	Rate of change	4w	15	20.5	34.1	20.3	1.2, 39.5	15	16.2	57.0	16.9	−1.6, 35.5	3.4	−23.2, 30.0	0.797
				8w	14	21.7	34.4	20.4	0.8, 39.9	14	24.4	56.6	25.2	6.6, 43.7	−4.8	−31.8, 22.1	0.716
				12w	13	21.1	36.2	25.5	5.5, 45.6	13	10.6	41.5	11.0	−8.0, 30.0	14.5	−13.1, 42.2	0.292
	Question 8_I feel clearheaded—I feel foggy headed	—	Measured value	Scr	16	12.2	6.6	—	—	16	14.6	6.8	—	—	−2.4	−7.2, 2.5	0.324
				4w	16	15.7	6.5	16.3	13.7, 18.9	16	15.4	6.3	14.6	11.9, 17.2	1.7	−2.1, 5.5	0.357
				8w	15	14.3	4.9	15.9	13.4, 18.4	16	13.1	7.1	12.1	9.7, 14.6	3.8	0.2, 7.3	0.038*
				12w	14	15.5	5.8	16.7	14.9, 18.6	16	14.3	5.2	13.3	11.5, 15.1	3.4	0.8, 6.0	0.012*
			Amount of change	4w	16	3.5	4.3	3.1	0.4, 5.7	16	0.8	6.9	1.4	−1.3, 4.0	1.7	−2.1, 5.5	0.357
				8w	15	2.9	4.8	2.7	0.2, 5.2	16	−1.5	5.4	−1.1	−3.5, 1.3	3.8	0.2, 7.3	0.038*
				12w	14	3.8	4.4	3.5	1.6, 5.3	16	−0.3	3.5	0.1	−1.7, 1.8	3.4	0.8, 6.0	0.012*
		%	Rate of change	4w	15	41.4	64.2	37.3	13.6, 61.0	16	23.9	54.6	29.6	6.6, 52.6	7.7	−25.5, 40.9	0.638
				8w	14	26.2	39.3	25.4	2.4, 48.4	16	−8.5	45.7	−6.8	−28.4, 14.8	32.2	0.4, 64.1	0.047*
				12w	13	34.2	44.1	32.6	14.8, 50.3	16	5.5	31.3	8.2	−8.3, 24.8	24.4	−0.1, 48.8	0.050
Females of FAS1	Question 14_I can answer a survey quickly and easily right now—It's troublesome to answer	—	Measured value	Scr	16	14.1	5.1	—	—	16	16.1	7.5	—	—	−2.0	−6.6, 2.6	0.383
				4w	16	17.5	6.1	18.1	15.7, 20.4	16	14.2	6.2	13.4	11.0, 15.8	4.6	1.2, 8.0	0.009*
				8w	15	16.7	5.9	17.9	14.8, 21.1	16	13.8	7.7	13.1	10.0, 16.2	4.8	0.4, 9.3	0.033*
				12w	14	17.8	6.3	19.1	16.6, 21.6	16	15.0	5.5	14.3	11.9, 16.7	4.8	1.3, 8.3	0.008*
			Amount of change	4w	16	3.4	4.6	3.1	0.8, 5.5	16	−1.9	5.5	−1.5	−3.9, 0.9	4.6	1.2, 8.0	0.009*
				8w	15	3.1	5.9	3.0	−0.1, 6.1	16	−2.3	6.8	−1.8	−4.9, 1.3	4.8	0.4, 9.3	0.033*
				12w	14	4.4	5.0	4.2	1.7, 6.7	16	−1.1	5.6	−0.6	−3.0, 1.8	4.8	1.3, 8.3	0.008*
		%	Rate of change	4w	16	36.2	61.4	32.5	9.6, 55.3	16	−1.1	40.5	4.0	−19.0, 26.9	28.5	−4.1, 61.1	0.085
				8w	15	45.1	104.7	42.1	2.9, 81.3	16	−12.5	41.2	−8.0	−46.6, 30.7	50.1	−5.4, 105.6	0.075
				12w	14	50.7	88.0	44.9	15.4, 74.4	16	1.8	33.4	7.2	−22.0, 36.4	37.7	−4.1, 79.5	0.076
	Question 2_I am concentrated—I am not concentrated	—	Measured value	Scr	16	15.4	4.7	—	—	16	14.3	5.1	—	—	1.2	−2.4, 4.7	0.499
				4w	16	17.4	5.1	17.0	15.1, 18.9	16	17.6	5.0	18.1	16.2, 20.0	−1.1	−3.8, 1.6	0.410
				8w	16	19.4	4.5	19.1	17.0, 21.2	16	16.6	5.3	17.0	14.9, 19.1	2.1	−0.8, 5.0	0.152
				12w	16	20.6	6.5	20.3	17.4, 23.2	15	18.9	6.0	19.0	16.1, 22.0	1.3	−2.9, 5.4	0.537
			Amount of change	4w	16	1.9	4.7	2.1	0.2, 4.0	16	3.4	2.9	3.2	1.3, 5.1	−1.1	−3.8, 1.6	0.410
				8w	16	3.9	4.9	4.1	2.1, 6.2	16	2.3	3.9	2.0	0.0, 4.1	2.1	−0.8, 5.0	0.152
				12w	16	5.1	6.7	5.3	2.4, 8.2	15	3.9	4.7	4.1	1.1, 7.0	1.3	−2.9, 5.4	0.537
		%	Rate of change	4w	16	16.8	35.5	19.1	0.0, 38.1	16	34.1	50.0	30.7	11.6, 49.9	−11.7	−38.8, 15.5	0.386
				8w	16	32.7	38.8	35.7	16.6, 54.7	16	27.9	54.7	23.5	4.4, 42.7	12.1	−15.0, 39.2	0.369
				12w	16	39.9	59.4	43.6	12.7, 74.5	15	31.8	38.7	43.4	12.2, 74.6	0.2	−43.8, 44.3	0.992
	Question 4_I am relaxed—I am stressed	—	Measured value	Scr	16	12.9	4.3	—	—	16	13.3	4.9	—	—	−0.3	−3.6, 3.0	0.849
				4w	16	15.0	6.0	15.2	12.4, 18.0	16	15.1	6.1	15.1	12.2, 17.9	0.1	−3.8, 4.1	0.950
				8w	16	17.6	4.6	17.7	15.0, 20.5	16	13.9	6.9	13.9	11.2, 16.6	3.9	0.0, 7.7	0.048*
				12w	16	18.9	5.4	19.0	16.2, 21.8	15	15.3	5.9	14.6	11.7, 17.4	4.5	0.4, 8.5	0.031*
			Amount of change	4w	16	2.1	5.6	1.9	−0.9, 4.8	16	1.8	5.8	1.8	−1.0, 4.6	0.1	−3.8, 4.1	0.950
				8w	16	4.6	4.9	4.5	1.8, 7.2	16	0.6	6.2	0.6	−2.1, 3.3	3.9	0.0, 7.7	0.048*
				12w	16	5.9	4.9	5.8	3.0, 8.6	15	1.1	6.7	1.3	−1.6, 4.2	4.5	0.4, 8.5	0.031*
		%	Rate of change	4w	16	23.4	59.5	20.1	−5.7, 45.9	15	16.7	47.8	20.2	−6.4, 46.9	−0.2	−37.4, 37.1	0.993
				8w	16	48.4	62.0	43.8	19.5, 68.2	15	9.0	48.5	13.9	−11.2, 39.1	29.9	−5.3, 65.1	0.093
				12w	16	55.2	56.7	51.0	27.2, 74.9	15	14.2	50.3	18.7	−6.0, 43.3	32.3	−2.2, 66.8	0.065
	Question 8_I feel clearheaded—I feel foggy headed	—	Measured value	Scr	16	13.8	3.7	—	—	16	10.6	5.0	—	—	3.1	0.0, 6.3	0.052
				4w	16	14.9	5.3	14.2	11.5, 16.8	16	14.4	5.6	15.2	12.5, 17.9	−1.1	−5.0, 2.8	0.581
				8w	16	15.8	3.7	15.0	12.4, 17.6	16	13.1	6.9	14.1	11.4, 16.8	0.9	−3.0, 4.8	0.637
				12w	16	16.8	6.0	15.4	12.6, 18.2	15	15.1	6.3	15.7	12.9, 18.5	−0.3	−4.4, 3.7	0.869
			Amount of change	4w	16	1.1	6.1	1.8	−0.8, 4.5	16	3.8	4.6	2.9	0.2, 5.6	−1.1	−5.0, 2.8	0.581
				8w	16	2.1	4.7	2.7	0.0, 5.3	16	2.5	5.8	1.8	−0.9, 4.4	0.9	−3.0, 4.8	0.637
				12w	16	3.0	5.0	3.1	0.3, 5.9	15	3.7	5.3	3.4	0.6, 6.2	−0.3	−4.4, 3.7	0.869
		%	Rate of change	4w	16	14.2	46.2	24.5	−0.3, 49.3	15	49.1	68.6	38.1	12.4, 63.8	−13.6	−50.1, 23.0	0.453
				8w	16	21.9	38.7	32.2	6.2, 58.1	15	37.8	75.9	26.9	0.0, 53.7	5.3	−32.9, 43.5	0.778
				12w	16	22.8	45.8	29.4	−0.4, 59.2	15	46.5	72.1	39.4	8.7, 70.2	−10.0	−53.9, 33.8	0.643
	Question 14_I can answer a survey quickly and easily right now—It's troublesome to answer	—	Measured value	Scr	16	14.3	4.9	—	—	16	14.4	6.5	—	—	−0.1	−4.3, 4.0	0.951
				4w	16	13.9	4.9	14.0	11.9, 16.0	16	14.6	5.4	14.5	12.5, 16.5	−0.6	−3.4, 2.3	0.697
				8w	16	14.4	5.1	14.5	11.8, 17.1	16	14.3	7.0	14.2	11.5, 16.8	0.3	−3.5, 4.0	0.887
				12w	16	16.4	7.0	16.4	13.3, 19.6	15	15.2	5.6	14.6	11.4, 17.8	1.8	−2.6, 6.3	0.405
			Amount of change	4w	16	−0.4	4.6	−0.4	−2.4, 1.6	16	0.1	4.5	0.2	−1.9, 2.2	−0.6	−3.4, 2.3	0.697
				8w	16	0.1	5.3	0.1	−2.5, 2.8	16	−0.2	5.9	−0.1	−2.8, 2.5	0.3	−3.5, 4.0	0.887
				12w	16	2.1	7.4	2.1	−1.0, 5.2	15	1.0	5.5	0.3	−2.9, 3.4	1.8	−2.6, 6.3	0.405
		%	Rate of change	4w	16	0.9	38.9	0.8	−17.7, 19.2	16	13.1	43.4	13.5	−5.0, 31.9	−12.7	−38.7, 13.4	0.328
				8w	16	6.7	40.7	6.6	−16.8, 30.1	16	1.9	52.0	2.1	−21.3, 25.6	4.5	−28.7, 37.7	0.784
				12w	16	21.2	67.1	21.0	−8.5, 50.6	15	25.3	62.1	20.8	−9.0, 50.7	0.2	−41.8, 42.2	0.992
Participants aged ≥ 40 years in FAS1	Question 2_I am concentrated—I am not concentrated	—	Measured value	Scr	20	16.4	6.2	—	—	20	17.0	6.0	—	—	−0.6	−4.5, 3.2	0.738
				4w	20	18.8	5.7	18.8	17.0, 20.6	20	18.2	5.9	17.8	16.0, 19.6	1.0	−1.6, 3.6	0.439
				8w	19	19.1	4.8	19.8	17.9, 21.6	20	18.1	5.5	17.7	15.9, 19.6	2.0	−0.6, 4.7	0.124
				12w	18	21.1	5.3	21.7	19.4, 24.0	20	18.9	6.0	18.5	16.3, 20.8	3.2	0.0, 6.4	0.052
			Amount of change	4w	20	2.4	4.7	2.4	0.6, 4.2	20	1.2	4.1	1.4	−0.5, 3.2	1.0	−1.6, 3.6	0.439
				8w	19	3.5	5.0	3.4	1.5, 5.2	20	1.1	4.1	1.3	−0.5, 3.1	2.0	−0.6, 4.7	0.124
				12w	18	5.7	5.7	5.3	3.0, 7.6	20	1.9	5.1	2.1	−0.1, 4.3	3.2	0.0, 6.4	0.052
		%	Rate of change	4w	20	27.6	54.3	27.4	11.4, 43.4	20	11.5	26.6	13.9	−2.2, 29.9	13.5	−9.1, 36.2	0.235
				8w	19	41.3	87.7	39.0	13.4, 64.5	20	13.1	33.9	16.6	−8.6, 41.9	22.3	−13.6, 58.3	0.216
				12w	18	55.3	72.6	50.1	29.5, 70.7	20	18.6	38.4	22.1	1.9, 42.4	27.9	−1.0, 56.9	0.058
	Question 4_I am relaxed—I am stressed	—	Measured value	Scr	20	13.5	5.7	—	—	20	14.1	4.8	—	—	−0.6	−4.0, 2.8	0.721
				4w	20	15.7	6.3	15.7	13.2, 18.2	20	16.4	5.2	16.3	13.8, 18.8	−0.5	−4.1, 3.0	0.758
				8w	19	18.2	5.3	18.6	16.0, 21.1	20	16.2	5.7	16.0	13.6, 18.5	2.5	−1.0, 6.0	0.156
				12w	18	18.4	6.2	18.7	16.2, 21.2	20	15.6	5.3	15.4	12.9, 17.8	3.4	−0.1, 6.8	0.059
			Amount of change	4w	20	2.2	5.4	2.1	−0.5, 4.6	20	2.4	7.4	2.6	0.1, 5.1	−0.5	−4.1, 3.0	0.758
				8w	19	5.2	5.5	4.9	2.4, 7.4	20	2.1	7.5	2.4	−0.1, 4.8	2.5	−1.0, 6.0	0.156
				12w	18	5.1	6.0	5.1	2.6, 7.6	20	1.5	6.1	1.7	−0.7, 4.1	3.4	−0.1, 6.8	0.059
		%	Rate of change	4w	19	22.0	58.7	22.8	0.8, 44.9	20	27.9	52.3	28.0	6.6, 49.5	−5.2	−36.0, 25.5	0.733
				8w	18	40.8	52.5	40.5	19.5, 61.4	20	25.1	52.6	25.2	5.1, 45.3	15.2	−13.8, 44.3	0.294
				12w	17	42.2	60.5	45.7	24.0, 67.4	20	18.3	43.9	18.4	−2.2, 39.0	27.3	−2.6, 57.2	0.072
	Question 8_I feel clearheaded—I feel foggy headed	—	Measured value	Scr	20	12.6	5.7	—	—	20	13.8	6.3	—	—	−1.3	−5.1, 2.6	0.516
				4w	20	15.7	6.1	16.0	13.7, 18.2	20	16.0	5.8	15.5	13.3, 17.8	0.5	−2.7, 3.6	0.771
				8w	19	15.3	4.4	16.2	13.9, 18.5	20	14.8	6.7	14.3	12.1, 16.5	1.9	−1.3, 5.1	0.233
				12w	18	17.1	5.4	17.7	15.7, 19.7	20	15.1	5.7	14.5	12.6, 16.5	3.2	0.4, 6.0	0.027*
			Amount of change	4w	20	3.2	5.5	2.9	0.7, 5.2	20	2.2	5.5	2.5	0.2, 4.7	0.5	−2.7, 3.6	0.771
				8w	19	3.4	5.0	3.2	0.9, 5.5	20	1.0	5.9	1.3	−1.0, 3.5	1.9	−1.3, 5.1	0.233
				12w	18	4.8	4.8	4.7	2.7, 6.7	20	1.3	4.6	1.5	−0.5, 3.4	3.2	0.4, 6.0	0.027*
		%	Rate of change	4w	19	37.3	64.4	36.1	13.7, 58.4	20	33.2	60.6	36.1	14.3, 57.9	−0.1	−31.3, 31.2	0.997
				8w	18	30.1	41.4	29.0	6.4, 51.6	20	22.7	67.9	24.9	3.1, 46.7	4.2	−27.3, 35.6	0.790
				12w	17	42.5	47.6	41.7	19.4, 63.9	20	24.7	62.1	26.9	5.8, 48.0	14.8	−16.0, 45.5	0.336
	Question 14_I can answer a survey quickly and easily right now—It's troublesome to answer	—	Measured value	Scr	20	13.8	4.7	—	—	20	14.2	6.8	—	—	−0.4	−4.2, 3.3	0.809
				4w	20	17.0	5.8	17.1	15.0, 19.1	20	14.2	5.7	14.0	11.9, 16.0	3.1	0.2, 5.9	0.034*
				8w	19	16.4	5.2	17.0	14.7, 19.3	20	14.9	6.7	14.6	12.4, 16.8	2.4	−0.8, 5.6	0.141
				12w	18	18.9	5.5	19.5	17.5, 21.6	20	15.1	4.5	14.9	12.9, 17.0	4.6	1.7, 7.5	0.003*
			Amount of change	4w	20	3.3	4.9	3.2	1.2, 5.2	20	0.0	4.8	0.1	−1.9, 2.1	3.1	0.2, 5.9	0.034*
				8w	19	3.1	5.6	3.2	0.9, 5.4	20	0.7	5.0	0.8	−1.5, 3.0	2.4	−0.8, 5.6	0.141
				12w	18	5.7	5.8	5.7	3.6, 7.8	20	0.9	5.2	1.1	−0.9, 3.1	4.6	1.7, 7.5	0.003*
		%	Rate of change	4w	20	34.8	59.7	34.4	15.1, 53.8	20	10.6	38.8	12.4	−7.0, 31.8	22.1	−5.3, 49.5	0.111
				8w	19	42.3	94.7	42.2	10.5, 73.9	20	7.5	44.5	9.2	−22.1, 40.5	33.0	−11.6, 77.7	0.142
					18	62.5	84.0	61.1	36.1, 86.0	20	23.3	54.9	26.0	1.4, 50.6	35.0	0.0, 70.1	0.050
Participants aged < 40 years in FAS1	Question 2_I am concentrated—I am not concentrated	—	Measured value	Scr	12	14.7	5.1	—	—	12	13.9	5.0	—	—	0.8	−3.5, 5.0	0.717
				4w	12	16.1	5.5	16.0	13.4, 18.5	12	16.2	3.9	16.4	13.8, 19.0	−0.4	−4.1, 3.2	0.810
				8w	12	18.3	5.5	18.1	15.2, 21.0	12	15.6	5.2	15.8	13.0, 18.7	2.3	−1.8, 6.4	0.258
				12w	12	19.2	8.2	19.0	15.0, 23.0	11	15.2	5.8	15.1	11.0, 19.1	4.0	−1.7, 9.7	0.163
			Amount of change	4w	12	1.4	5.1	1.5	−1.0, 4.1	12	2.3	4.8	2.0	−0.6, 4.5	−0.4	−4.1, 3.2	0.810
				8w	12	3.6	5.4	3.7	0.8, 6.6	12	1.7	5.2	1.4	−1.5, 4.3	2.3	−1.8, 6.4	0.258
				12w	12	4.5	8.0	4.6	0.6, 8.6	11	0.4	5.3	0.6	−3.4, 4.7	4.0	−1.7, 9.7	0.163
		%	Rate of change	4w	12	14.3	39.3	15.7	−8.6, 40.1	12	32.4	60.2	29.0	4.7, 53.4	−13.3	−47.8, 21.2	0.431
				8w	12	31.5	42.9	32.9	4.9, 60.9	12	24.5	64.4	21.4	−6.7, 49.4	11.5	−28.2, 51.2	0.552
				12w	12	36.7	70.9	38.4	−7.6, 84.5	11	3.2	37.2	23.7	−22.7, 70.2	14.7	−50.8, 80.2	0.640
	Question 4_I am relaxed—I am stressed	—	Measured value	Scr	12	13.9	4.8	—	—	12	14.8	6.8	—	—	−0.9	−5.9, 4.1	0.707
				4w	12	16.8	5.9	17.1	14.0, 20.2	12	13.2	5.7	13.0	10.0, 16.1	4.1	−0.3, 8.4	0.065
				8w	12	16.5	4.9	16.8	13.8, 19.8	12	13.8	6.3	13.6	10.6, 16.6	3.2	−1.1, 7.5	0.137
				12w	12	18.8	6.9	19.3	15.9, 22.8	11	15.4	6.6	13.6	10.1, 17.2	5.7	0.8, 10.6	0.026*
			Amount of change	4w	12	2.8	3.2	2.5	−0.6, 5.6	12	−1.7	7.4	−1.5	−4.6, 1.5	4.1	−0.3, 8.4	0.065
				8w	12	2.6	4.5	2.2	−0.8, 5.3	12	−1.1	6.9	−0.9	−4.0, 2.1	3.2	−1.1, 7.5	0.137
				12w	12	4.8	4.0	4.8	1.3, 8.2	11	−0.8	7.0	−0.9	−4.5, 2.6	5.7	0.8, 10.6	0.026*
		%	Rate of change	4w	12	22.0	26.0	18.1	−2.1, 38.3	11	−4.4	46.1	−0.2	−21.3, 20.9	18.3	−11.3, 47.9	0.212
				8w	12	28.6	52.5	21.6	−3.9, 47.2	11	2.2	51.5	9.9	−16.8, 36.6	11.7	−25.7, 49.2	0.521
				12w	12	36.7	34.9	33.5	9.2, 57.9	11	1.7	47.8	5.1	−20.3, 30.6	28.4	−7.3, 64.1	0.112
	Question 8_I feel clearheaded—I feel foggy headed	—	Measured value	Scr	12	13.7	4.8	—	—	12	10.6	5.6	—	—	3.1	−1.3, 7.5	0.160
				4w	12	14.6	5.6	14.0	10.7, 17.3	12	13.1	5.9	13.9	10.5, 17.2	0.1	−4.7, 4.9	0.964
				8w	12	14.8	4.4	13.9	11.0, 16.8	12	10.3	6.6	11.4	8.5, 14.3	2.5	−1.6, 6.7	0.220
				12w	12	14.8	6.4	13.6	10.7, 16.5	11	13.9	5.7	14.3	11.4, 17.3	−0.7	−4.9, 3.4	0.714
			Amount of change	4w	12	0.9	4.9	1.7	−1.6, 5.0	12	2.5	6.9	1.6	−1.8, 4.9	0.1	−4.7, 4.9	0.964
				8w	12	1.1	3.9	1.6	−1.3, 4.5	12	−0.3	5.9	−0.9	−3.8, 2.0	2.5	−1.6, 6.7	0.220
				12w	12	1.2	3.6	1.3	−1.6, 4.2	11	2.4	5.5	2.0	−0.9, 5.0	−0.7	−4.9, 3.4	0.714
		%	Rate of change	4w	12	11.6	38.0	18.8	−7.5, 45.1	11	41.5	67.3	33.5	6.0, 61.0	−14.7	−53.3, 23.9	0.436
				8w	12	14.5	32.7	17.0	−12.2, 46.2	11	−1.9	60.7	−4.6	−35.1, 25.9	21.6	−21.3, 64.4	0.306
				12w	12	7.3	31.5	8.5	−17.8, 34.9	11	26.5	52.1	25.2	−2.4, 52.7	−16.6	−55.3, 22.0	0.381
	Question 14_I can answer a survey quickly and easily right now—It's troublesome to answer	—	Measured value	Scr	12	14.9	5.3	—	—	12	17.0	7.1	—	—	−2.1	−7.4, 3.3	0.426
				4w	12	13.6	5.1	14.2	11.8, 16.6	12	14.7	6.1	14.0	11.6, 16.4	0.2	−3.2, 3.7	0.883
				8w	12	14.2	6.0	14.8	11.1, 18.5	12	12.7	8.1	12.0	8.3, 15.7	2.8	−2.4, 8.0	0.279
				12w	12	14.3	7.3	15.0	11.5, 18.4	11	15.1	7.1	13.8	10.3, 17.3	1.2	−3.8, 6.1	0.627
			Amount of change	4w	12	−1.3	3.5	−1.7	−4.1, 0.7	12	−2.3	5.3	−1.9	−4.4, 0.5	0.2	−3.2, 3.7	0.883
				8w	12	−0.8	5.3	−1.1	−4.8, 2.5	12	−4.3	7.3	−3.9	−7.6, −0.3	2.8	−2.4, 8.0	0.279
				12w	12	−0.7	5.4	−0.9	−4.4, 2.5	11	−1.8	6.0	−2.1	−5.6, 1.4	1.2	−3.8, 6.1	0.627
		%	Rate of change	4w	12	−8.6	26.0	−11.5	−32.3, 9.3	12	−1.7	47.4	1.4	−19.4, 22.2	−12.9	−42.6, 16.7	0.374
				8w	12	−1.8	35.5	−2.2	−26.8, 22.5	12	−26.6	43.9	−26.2	−50.9, −1.5	24.0	−11.1, 59.1	0.170
					12	−6.3	43.1	−7.2	−31.8, 17.4	11	−5.2	34.7	−8.2	−33.1, 16.7	1.0	−34.3, 36.3	0.954
Participants whose score of sleepiness on rising in the OSA‐MA above the median (14.15 point) at Scr in FAS1	Question 2_I am concentrated—I am not concentrated	—	Measured value	Scr	16	19.4	5.1	—	—	16	19.9	4.1	—	—	−0.5	−3.9, 2.9	0.763
				4w	16	20.1	4.9	20.1	17.9, 22.3	16	20.3	4.9	20.0	17.8, 22.2	0.1	−3.0, 3.2	0.958
				8w	15	21.8	2.7	22.5	20.3, 24.6	16	20.1	5.0	19.9	17.8, 22.0	2.6	−0.4, 5.6	0.090
				12w	15	23.8	4.3	24.3	21.5, 27.1	16	20.6	5.7	20.6	17.8, 23.3	3.8	−0.2, 7.7	0.061
			Amount of change	4w	16	0.7	4.4	0.7	−1.5, 2.9	16	0.4	5.1	0.6	−1.6, 2.8	0.1	−3.0, 3.2	0.958
				8w	15	3.2	4.9	3.1	1.0, 5.2	16	0.3	4.7	0.5	−1.6, 2.6	2.6	−0.4, 5.6	0.090
				12w	15	5.2	7.4	4.9	2.1, 7.7	16	0.8	5.8	1.2	−1.6, 3.9	3.8	−0.2, 7.7	0.061
		%	Rate of change	4w	16	8.3	30.0	8.3	−3.6, 20.1	16	4.0	24.8	5.6	−6.3, 17.5	2.7	−14.2, 19.5	0.749
				8w	15	24.7	37.8	22.8	10.4, 35.2	16	3.2	24.9	5.5	−6.6, 17.7	17.2	−0.2, 34.6	0.052
				12w	15	39.9	61.4	36.9	18.4, 55.5	16	5.9	28.6	9.3	−8.8, 27.5	27.6	1.5, 53.7	0.039*
	Question 4_I am relaxed—I am stressed	—	Measured value	Scr	16	17.8	3.4	—	—	16	17.2	5.4	—	—	0.6	−2.7, 3.8	0.726
				4w	16	18.9	5.8	18.8	16.0, 21.6	16	16.3	5.0	16.4	13.6, 19.1	2.4	−1.5, 6.4	0.216
				8w	15	20.9	4.0	21.0	18.6, 23.5	16	18.0	5.0	18.0	15.6, 20.3	3.1	−0.3, 6.4	0.074
				12w	15	22.1	4.8	22.2	19.7, 24.7	16	18.5	4.8	18.6	16.2, 21.0	3.6	0.2, 7.1	0.039*
			Amount of change	4w	16	1.1	4.0	1.4	−1.4, 4.2	16	−0.9	8.0	−1.0	−3.8, 1.8	2.4	−1.5, 6.4	0.216
				8w	15	3.4	4.4	3.7	1.3, 6.1	16	0.8	8.3	0.6	−1.7, 3.0	3.1	−0.3, 6.4	0.074
				12w	15	4.6	4.6	4.8	2.4, 7.3	16	1.3	6.3	1.2	−1.2, 3.6	3.6	0.2, 7.1	0.039*
		%	Rate of change	4w	16	5.4	25.5	6.9	−9.6, 23.4	16	4.4	44.3	3.6	−12.8, 20.1	3.2	−20.1, 26.6	0.779
				8w	15	22.5	28.8	23.9	7.8, 39.9	16	16.5	53.3	15.3	−0.4, 30.9	8.6	−13.8, 31.0	0.439
				12w	15	29.0	30.2	30.3	14.5, 46.2	16	16.2	44.0	15.3	−0.2, 30.8	15.1	−7.1, 37.2	0.175
	Question 8_I feel clearheaded—I feel foggy headed	—	Measured value	Scr	16	16.8	3.7	—	—	16	17.1	4.9	—	—	−0.3	−3.4, 2.9	0.872
				4w	16	17.8	5.6	17.8	14.7, 20.9	16	17.1	6.4	17.1	14.0, 20.1	0.7	−3.6, 5.1	0.728
				8w	15	16.7	3.8	17.3	14.6, 20.0	16	16.8	6.0	16.7	14.1, 19.3	0.6	−3.2, 4.4	0.740
				12w	15	19.4	4.0	19.8	17.6, 22.0	16	18.3	4.5	18.2	16.1, 20.4	1.6	−1.5, 4.7	0.310
			Amount of change	4w	16	1.0	6.0	1.0	−2.1, 4.1	16	0.1	7.5	0.3	−2.8, 3.4	0.7	−3.6, 5.1	0.728
				8w	15	0.4	4.1	0.5	−2.2, 3.2	16	−0.3	6.9	−0.1	−2.7, 2.6	0.6	−3.2, 4.4	0.740
				12w	15	3.1	3.9	3.0	0.8, 5.2	16	1.3	5.9	1.4	−0.7, 3.6	1.6	−1.5, 4.7	0.310
		%	Rate of change	4w	16	10.6	41.9	10.8	−10.2, 31.8	16	8.6	54.3	10.3	−10.8, 31.3	0.5	−29.2, 30.2	0.973
				8w	15	5.5	28.1	5.8	−12.1, 23.7	16	6.5	49.8	7.9	−9.8, 25.5	−2.0	−27.1, 23.1	0.870
				12w	15	21.8	30.2	21.1	4.3, 37.9	16	16.1	47.7	17.6	1.2, 34.0	3.5	−20.0, 27.0	0.765
	Question 14_I can answer a survey quickly and easily right now—It's troublesome to answer	—	Measured value	Scr	16	17.9	3.3	—	—	16	19.9	5.8	—	—	−2.1	−5.5, 1.4	0.230
				4w	16	18.4	3.9	18.9	16.6, 21.1	16	17.3	5.4	16.8	14.6, 19.1	2.0	−1.2, 5.2	0.205
				8w	15	17.5	5.8	18.6	15.0, 22.2	16	17.5	7.4	17.1	13.5, 20.6	1.5	−3.6, 6.6	0.546
				12w	15	19.5	4.6	20.2	17.7, 22.6	16	17.6	4.6	17.3	14.9, 19.7	2.9	−0.6, 6.4	0.105
			Amount of change	4w	16	0.6	4.9	0.0	−2.2, 2.3	16	−2.6	5.1	−2.0	−4.2, 0.2	2.0	−1.2, 5.2	0.205
				8w	15	−0.1	6.5	−0.2	−3.8, 3.3	16	−2.4	7.8	−1.8	−5.3, 1.8	1.5	−3.6, 6.6	0.546
				12w	15	1.9	5.7	1.3	−1.2, 3.8	16	−2.4	5.9	−1.6	−4.0, 0.9	2.9	−0.6, 6.4	0.105
		%	Rate of change	4w	16	6.4	29.5	3.6	−9.3, 16.5	16	−9.9	26.7	−6.7	−19.7, 6.2	10.3	−8.2, 28.8	0.263
				8w	15	2.2	41.4	0.9	−20.0, 21.7	16	−7.3	42.4	−3.7	−24.2, 16.8	4.5	−25.1, 34.2	0.757
					15	14.1	34.9	11.0	−3.5, 25.6	16	−6.5	31.2	−2.2	−16.5, 12.1	13.2	−7.5, 34.0	0.202
Participants whose score of sleepiness on rising in OSA‐MA below the median (14.15 point) at Scr in FAS1	Question 2_I am concentrated—I am not concentrated	—	Measured value	Scr	16	12.1	3.7	—	—	16	11.8	4.1	—	—	0.3	−2.6, 3.1	0.858
				4w	16	15.4	5.7	15.4	13.3, 17.4	16	14.6	4.0	14.7	12.6, 16.8	0.6	−2.3, 3.6	0.665
				8w	16	15.9	5.0	15.9	13.7, 18.1	16	14.2	4.2	14.2	12.0, 16.5	1.6	−1.5, 4.8	0.297
				12w	15	16.9	6.7	16.9	14.2, 19.6	15	14.3	4.8	14.3	11.5, 17.0	2.6	−1.2, 6.5	0.174
			Amount of change	4w	16	3.4	5.0	3.4	1.3, 5.5	16	2.8	3.2	2.8	0.7, 4.9	0.6	−2.3, 3.6	0.665
				8w	16	3.9	5.4	3.9	1.7, 6.2	16	2.4	4.0	2.3	0.1, 4.5	1.6	−1.5, 4.8	0.297
				12w	15	5.3	6.0	5.0	2.2, 7.7	15	2.0	4.4	2.3	−0.4, 5.1	2.6	−1.2, 6.5	0.174
		%	Rate of change	4w	16	36.9	60.1	37.9	14.1, 61.7	16	34.6	51.5	33.5	9.7, 57.4	4.4	−29.3, 38.1	0.793
				8w	16	49.5	94.8	50.9	18.0, 83.9	16	31.6	59.3	30.0	−3.0, 63.0	20.9	−25.7, 67.6	0.366
				12w	15	55.8	81.4	53.8	20.8, 86.7	15	20.9	45.9	31.8	−1.1, 64.8	21.9	−24.7, 68.6	0.344
	Question 4_I am relaxed—I am stressed	—	Measured value	Scr	16	9.5	3.2	—	—	16	11.5	4.1	—	—	−2.0	−4.7, 0.7	0.140
				4w	16	13.3	5.1	13.6	10.7, 16.5	16	14.1	5.9	13.8	11.0, 16.7	−0.2	−4.3, 3.9	0.918
				8w	16	14.4	3.9	14.4	11.8, 17.0	16	12.5	5.7	12.5	9.9, 15.1	1.9	−1.9, 5.6	0.313
				12w	15	15.0	5.8	15.2	12.3, 18.1	15	12.3	4.7	11.7	8.7, 14.6	3.6	−0.7, 7.8	0.097
			Amount of change	4w	16	3.8	4.9	2.9	0.1, 5.8	16	2.6	6.9	3.2	0.3, 6.0	−0.2	−4.3, 3.9	0.918
				8w	16	4.9	5.9	3.7	1.1, 6.3	16	1.0	6.4	1.9	−0.7, 4.4	1.9	−1.9, 5.6	0.313
				12w	15	5.3	5.9	4.6	1.7, 7.5	15	0.0	6.7	1.0	−1.9, 3.9	3.6	−0.7, 7.8	0.097
		%	Rate of change	4w	15	39.7	60.1	24.6	−2.5, 51.7	15	29.3	57.6	43.1	16.2, 70.0	−18.5	−58.3, 21.3	0.348
				8w	15	49.4	66.2	29.9	6.7, 53.2	15	17.5	53.5	35.2	12.1, 58.3	−5.3	−39.5, 28.9	0.753
				12w	14	51.6	65.5	41.2	14.2, 68.1	15	8.2	47.6	21.3	−5.3, 47.8	19.9	−19.4, 59.3	0.308
	Question 8_I feel clearheaded—I feel foggy headed	—	Measured value	Scr	16	9.1	3.6	—	—	16	8.1	3.4	—	—	1.0	−1.6, 3.6	0.432
				4w	16	12.8	5.1	12.4	10.5, 14.3	16	12.6	4.5	13.2	11.3, 15.1	−0.8	−3.4, 1.9	0.556
				8w	16	13.6	4.3	13.3	11.0, 15.6	16	9.4	5.7	9.8	7.5, 12.1	3.5	0.2, 6.8	0.038*
				12w	15	12.9	5.6	12.5	10.2, 14.8	15	10.7	3.9	10.6	8.3, 13.0	1.9	−1.4, 5.2	0.255
			Amount of change	4w	16	3.6	4.4	3.7	1.8, 5.5	16	4.5	2.5	4.4	2.6, 6.3	−0.8	−3.4, 1.9	0.556
				8w	16	4.4	4.4	4.6	2.3, 6.8	16	1.3	4.6	1.1	−1.2, 3.4	3.5	0.2, 6.8	0.038*
				12w	15	3.7	5.4	3.8	1.4, 6.1	15	2.1	3.6	1.9	−0.5, 4.2	1.9	−1.4, 5.2	0.255
		%	Rate of change	4w	15	45.2	65.4	52.4	26.8, 78.0	15	65.5	57.4	57.5	31.9, 83.2	−5.1	−41.8, 31.5	0.775
				8w	15	42.3	39.2	46.0	13.6, 78.4	15	21.9	80.0	17.8	−14.7, 50.2	28.3	−18.0, 74.6	0.221
				12w	14	34.5	56.8	39.5	8.5, 70.5	15	35.2	67.4	29.9	−0.6, 60.4	9.6	−34.3, 53.5	0.658
	Question 14_I can answer a survey quickly and easily right now—It's troublesome to answer	—	Measured value	Scr	16	10.5	3.2	—	—	16	10.6	4.2	—	—	−0.1	−2.8, 2.6	0.963
				4w	16	13.0	6.1	12.9	10.5, 15.3	16	11.4	4.5	11.3	8.9, 13.7	1.6	−1.8, 5.0	0.338
				8w	16	13.7	4.8	13.6	11.4, 15.8	16	10.6	5.3	10.4	8.2, 12.7	3.2	0.0, 6.3	0.048*
				12w	15	14.7	7.5	15.2	12.1, 18.2	15	12.5	5.2	12.2	9.1, 15.3	3.0	−1.4, 7.3	0.169
			Amount of change	4w	16	2.5	4.9	2.5	0.1, 4.9	16	0.9	4.5	0.9	−1.5, 3.3	1.6	−1.8, 5.0	0.338
				8w	16	3.2	4.4	3.2	1.0, 5.4	16	0.0	4.4	0.0	−2.2, 2.3	3.2	0.0, 6.3	0.048*
				12w	15	4.5	6.9	4.8	1.7, 7.8	15	2.4	4.0	1.8	−1.3, 4.9	3.0	−1.4, 7.3	0.169
		%	Rate of change	4w	16	30.7	69.0	31.5	4.9, 58.1	16	22.0	48.8	23.3	−3.3, 49.9	8.2	−29.4, 45.8	0.659
				8w	16	46.8	100.1	47.3	7.2, 87.5	16	−3.2	52.0	−2.4	−42.6, 37.8	49.7	−7.1, 106.6	0.084
					15	55.8	101.6	57.5	18.6, 96.4	15	34.2	58.3	30.5	−8.5, 69.4	27.1	−27.9, 82.0	0.323

Abbreviations: 12w, 12 weeks after consumption; 4w, 4 weeks after consumption; 8w, 8 weeks after consumption; 95% CI, 95% confidence interval; Amount of change, amount of change from Scr; EMM, estimated marginal mean; FAS1, full analysis set1; OSA‐MA, OSA sleep inventory MA version; Rate of change, rate of change from Scr; Scr, screening (before consumption); SD, standard deviation; Δ, Difference between groups (BE group–placebo group).

* *p* < 0.05.

In FAS1 participants and those aged ≥ 40 years, the measured value and amount of change of sleepiness on rising at 12w were significantly higher in the BE group than in the placebo group (Figure 3c). For the sleepiness on rising subitems, the BE group had significantly higher measured value and amount of change at 12w for “Question 8_I feel clearheaded–I feel foggy headed” and at 4w and 12w for “Question 14_I can answer a survey quickly and easily right now–It's troublesome to answer” than the Placebo group (Table 5). In FAS1 participants and those aged < 40 years, there were no significant differences between groups in the scores of sleepiness on rising (Figure 3d); however, in the subitems, the BE group recorded significantly higher measured value and amount of change at 12w for “Question 4_I am relaxed–I am stressed” than the Placebo group (Table 5).

In FAS1 participants and those whose sleepiness on rising score was above or equal to the median (14.15 points) at Scr, the BE group had significantly higher measured value and amount of change in sleepiness on rising at 12w than the Placebo group (Figure 3e). For the sleepiness on rising subitems, the BE group recorded a significantly higher rate of change at 12w for “Question 2_I am concentrated–I am not concentrated” and the measured value and amount of change at 12w for “Question 4_I am relaxed–I am stressed” than the Placebo group (Table 5). In FAS1 participants and those whose sleepiness on rising was below the median (14.15 points) at Scr, the BE group recorded significantly higher measured value and amount of change in sleepiness on rising at 8w than the Placebo group (Figure 3f). For the sleepiness on rising subitems, the BE group had significantly higher measured value and amount of change at 8w for “Question 8_I feel clearheaded–I feel foggy headed” and at 8w for “Question 14_I can answer a survey quickly and easily right now–It's troublesome to answer” than the Placebo group (Table 5).

### 3.3 VAS of Subjective Symptoms

Compared with the Placebo group, the BE group had significantly higher measured values and amount of change at 4w, 8w, and 12w for stiff shoulders and the measured value and amount of change at 8w for eye fatigue. The BE group had significantly lower measured values and the amount and rate of change at 12w for dry mouth than the Placebo group (Appendix S4). No significant inter‐group differences were observed for other items or time points (data not shown).

The results for each subgroup were confirmed for dry mouth, which showed significant improvement after the intervention in FAS1.

In FAS1 participants and those aged ≥ 40 years, the rate of change for dry mouth at 12w was significantly lower in the BE group than in the Placebo group (Appendix S5). In contrast, no significant inter‐group difference was found in FAS1 participants and those aged < 40 years (Appendix S5).

In both subgroups, FAS1 participants and those whose scores on sleepiness on rising were above or equal to the median (14.15 points) at Scr and FAS1 participants and those whose scores were below the median (14.15 points) at Scr, the rate of change at 12w was significantly lower in the BE group than in the Placebo group (Appendix S5).

In the sex subgroup, no significant inter‐group differences were found in any of the items (data not shown).

### 3.4 PSQI‐J

For each score, only daytime dysfunction (C7) showed a significantly higher rate of change at 12w in the BE group than in the Placebo group, and no significant inter‐group differences were found for the other items (data not shown). Regarding the subitem of daytime dysfunction (C7), “During the past month, how much of a problem has it been for you to keep up enough enthusiasm to get things done?”, the rate of change at 12w was significantly higher in the BE group than in the Placebo group (data not shown). Because lower scores and values for the PSQI‐J subitems indicated better outcomes, the effect of the test food on sleep quality was not determined by the PSQI‐J.

### 3.5 POMS2

There were no significant inter‐group differences (data not shown).

### 3.6 Blood Flow Test (Blood Flow)

There were no significant inter‐group differences (data not shown).

### 3.7 Skin Surface Temperature Test (Palmar Surface Temperature)

**FIGURE 4:**
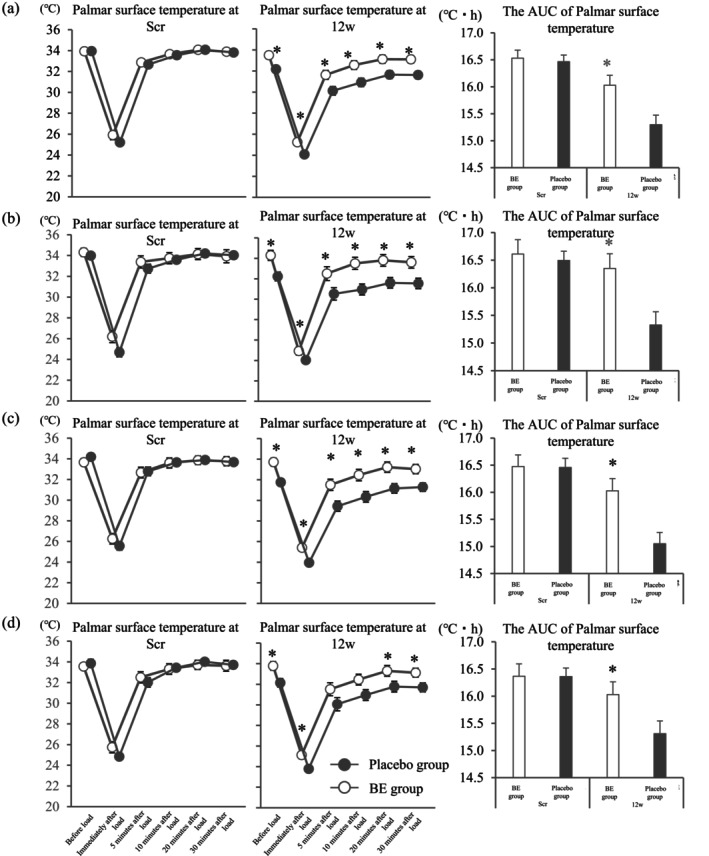
Measured value of the palmar surface temperature and its AUC of in FAS3 and each subgroup. Data of the palmar surface temperature in FAS3 (a) and each subgroup (b, males of FAS3; c, participants aged ≥ 40 years in FAS3; d, participants whose score of sleepiness on rising in OSA‐MA was below the median (14.15 point) at Scr in FAS3 are shown as mean and standard error (SE) at Scr and as estimated marginal mean and SE at 12w. (a) BE group (Scr, 4w, 8w, and 12w: *n* = 29) and placebo group (Scr, 4w, 8w, and 12w: *n* = 31), (b) BE group (Scr, 4w, 8w and 12w: *n* = 13) and placebo group (Scr, 4w, 8w and 12w: *n* = 16), (c) BE group (Scr, 4w, 8w, and 12w: *n* = 17) and placebo group (Scr, 4w, 8w, and 12w: *n* = 20), (d) BE group and placebo group (Scr, 4w, 8w, and 12w: *n* = 15). Scr, screening (baseline); 12w, 12 weeks after consumption. *p < 0.05.

In the overall analysis (FAS3), the BE group had significantly higher AUC of the palmar surface temperature and the palmar surface temperature before load, immediately after load, and 5, 10, 20, and 30 min after the cold‐water load at 12w than the Placebo group (Figure 4a). Significant differences were also observed in the amount and rate of change for the same items and time points (Appendix S6) but were not noted in other items (data not shown).

The effect of sex on the skin surface temperature was also evaluated. For females of FAS3, the BE group had significantly higher the palmar surface temperature immediately the cold‐water load at 12w than the Placebo group (Appendix S7). In contrast, for males of FAS3, the BE group had significantly higher AUC of the palmar surface temperature and the palmar surface temperature before load, immediately after load, and 5, 10, 20, and 30 min after the cold‐water load at 12w than the Placebo group (Figure 4b).

Regarding age, the BE group had significantly higher AUC of the palmar surface temperature at 12w and the palmar surface temperature before load, immediately after load, and 5, 10, 20, and 30 min after the cold‐water load at 12w than the Placebo group only in FAS3 participants and participants aged ≥ 40 years (Figure 4c).

Regarding the effect of sleepiness on rising, the BE group had significantly higher AUC of the palmar surface temperature at 12w and the palmar surface temperature before load, immediately after load, and 20 and 30 min after the cold‐water load at 12w than the Placebo group in FAS3 participants and those whose score on sleepiness on rising was below the median (14.15 points) at Scr (Figure 4d). However, there were no significant differences in FAS3 participants and those whose score on sleepiness on rising was above or equal to the median (14.15 points) at Scr (data not shown).

### 3.8 Sleep Test

In the overall analysis (FAS4), the BE group had a significantly lower rate of change in sleep‐onset latency at 12w than the Placebo group (Table 6). No significant inter‐group differences were observed for other parameters or time points (data not shown).

**TABLE 6:** Comparison of sleep onset latency in sleep test (FAS4).

Analysis set	Item	Unit	Classification	Time point	BE group					Placebo group					Group comparison		
					*n*	Mean	SD	EMM	95% CI	*n*	Mean	SD	EMM	95% CI	Δ	95% CI	*p*
FAS4	Sleep onset latency	second	Measured value	Scr	29	893.3	754.3	—	—	31	706.0	806.9	—	—	187.3	−216.1, 590.8	0.357
				12w	29	797.1	906.6	755.9	437.4, 1074.3	31	782.4	911.1	821.0	513.0, 1128.9	−65.1	−509.7, 379.5	0.770
			Amount of change	12w	29	−96.2	798.1	−40.6	−359.1, 277.8	31	76.5	1085.4	24.5	−283.5, 332.4	−65.1	−509.7, 379.5	0.770
		%	Rate of change	12w	29	24.8	112.7	34.2	−22.8, 91.1	31	125.3	208.3	116.6	61.5, 171.6	−82.4	−161.9, −2.9	0.042*

Abbreviations: 12w, 12 weeks after consumption; 95% CI, 95% confidence interval; Amount of change, amount of change from Scr; EMM, estimated marginal mean; FAS4, full analysis set4; Rate of change, rate of change from Scr; Scr, screening (before consumption); SD, standard deviation; Δ, Difference between groups (BE group–placebo group).

* *p* < 0.05.

Sex, age, or sleepiness on rising in OSA‐MA did not influence the sleep‐onset latency. In the analysis of sex subgroups, the measured value and amount of change of the TST at 12w in the female participants of FAS4 were significantly higher in the BE group than in the Placebo group; however, no significant inter‐group differences in TST were observed in male participants (Table 7). In the subgroup focusing on sleepiness on rising, although no items were significantly different between the groups in FAS4 participants and sleepiness on rising above or equal to the median (14.15 points) at Scr, FAS4 participants and those whose sleepiness on rising was below the median (14.15 points) at Scr showed significantly higher measured values and amounts of changes at 12w for the N3 total time in the BE group than in the Placebo group (Table 8). In the age subgroup, no significant inter‐group differences were noted for any of the items (data not shown).

**TABLE 7:** Comparison of total sleep time in sleep tests by sex difference.

Analysis set	Items	Unit	Classification	Time point	BE group					Placebo group					Group comparison		
					*n*	Mean	SD	EMM	95% CI	*n*	Mean	SD	EMM	95% CI	Δ	95% CI	*p*
Males of FAS4	Total sleep time (TST)	second	Measured value	Scr	13	18899.7	4210.5	—	—	16	19119.6	3409.1	—	—	−219.9	−3210.7, 2771.0	0.880
				12w	13	18837.7	5736.5	18916.2	15780.0, 22052.4	16	18541.6	6075.8	18477.8	15651.0, 21304.6	438.5	−3784.6, 4661.5	0.833
			Amount of change	12w	13	−62.0	5285.6	−104.8	−3241.0, 3031.4	16	−578.0	5769.6	−543.2	−3370.0, 2283.6	438.5	−3784.6, 4661.5	0.833
		%	Rate of change	12w	13	2.1	30.3	1.8	−15.2, 18.9	16	−2.1	30.9	−1.9	−17.3, 13.5	3.7	−19.3, 26.7	0.742
Females of FAS1	Total sleep time (TST)	second %	Measured value	Scr	16	19471.5	3951.0	—	—	15	20551.1	4842.3	—	—	−1079.6	−4348.4, 2189.3	0.504
				12w	16	21496.1	4153.6	21837.8	20068.2, 23607.5	15	19393.4	4734.1	19028.8	17200.7, 20857.0	2809.0	254.5, 5363.5	0.032*
			Amount of change	12w	16	2024.6	4029.7	1844.0	74.3, 3613.6	15	−1157.7	3332.1	−965.0	−2793.2, 863.1	2809.0	254.5, 5363.5	0.032*
			Rate of change	12w	16	13.2	24.2	11.9	2.0, 21.9	15	−3.9	19.6	−2.4	−12.7, 7.9	14.4	0.0, 28.7	0.050

Abbreviations: 12w, 12 weeks after consumption; 95% CI, 95% confidence interval; Amount of change, amount of change from Scr; EMM, estimated marginal mean; FAS4, full analysis set4; Rate of change, rate of change from Scr; Scr, screening (before consumption); SD, standard deviation; Δ, Difference between groups (BE group–placebo group).

* *p* < 0.05.

**TABLE 8:** Comparison of N3 total time in sleep test for different sleep quality.

Analysis set	Items	Unit	Classification	Time point	BE group					Placebo group					Group comparison		
					*n*	Mean	SD	EMM	95% CI	*n*	Mean	SD	EMM	95% CI	Δ	95% CI	*p*
Participants whose score of Sleepiness on rising in the OSA‐MA above the median (14.15 point) at Scr in FAS4	N3 total time	second	Measured value	Scr	15	3732.0	1635.0	—	—	16	2475.0	2001.9	—	—	1257.0	−83.2, 2597.2	0.065
				12w	15	3124.0	1574.2	2663.7	1905.1, 3422.4	16	2191.9	2137.2	2623.4	1890.2, 3356.6	40.3	−1045.3, 1126.0	0.940
			Amount of change	12w	15	−608.0	1326.8	−419.5	−1178.2, 339.2	16	−283.1	1585.4	−459.8	−1193.0, 273.3	40.3	−1045.3, 1126.0	0.940
		%	Rate of change	12w	14	−10.9	36.7	6.6	−37.4, 50.7	15	28.7	114.4	12.4	−30.1, 54.9	−5.7	−69.4, 57.9	0.854
Participants whose score of Sleepiness on rising in OSA‐MA below the median (14.15 point) at Scr in FAS4	N3 total time	second	Measured value	Scr	14	2171.8	1641.5	—	—	15	2092.0	1378.3	—	—	79.8	−1082.5, 1242.1	0.889
				12w	14	3053.6	1953.0	3020.6	2452.0, 3589.3	15	2123.0	1118.3	2153.7	1604.4, 2703.1	866.9	76.1, 1657.7	0.033*
			Amount of change	12w	14	881.8	1045.8	890.1	321.5, 1458.8	15	31.0	1073.8	23.2	−526.2, 572.6	866.9	76.1, 1657.7	0.033*
		%	Rate of change	12w	11	42.9	69.4	48.1	9.4, 86.8	14	7.9	59.7	3.8	−30.4, 38.0	44.3	−7.9, 96.5	0.092

Abbreviations: 12w, 12 weeks after consumption; 95% CI, 95% confidence interval; Amount of change, amount of change from Scr; EMM, estimated marginal mean; FAS4, full analysis set4; OSA‐MA, OSA sleep inventory MA version; Rate of change, rate of change from Scr; Scr, screening (before consumption); SD, standard deviation; Δ, Difference between groups (BE group–placebo group).

* *p* < 0.05.

### 3.9 Blood Test

In the overall analysis (FAS5), the BE group demonstrated a significantly lower rate of change in TBARS at 12w than the Placebo group (Table 9). No significant inter‐group differences were found for other items or time points (data not shown).

**TABLE 9:** Comparison of 2‐thiobarbituric acid reactive substances (TBARS).

Analysis set	Unit	Classification	Time point	BE group					Placebo group					Group comparison		
				*n*	Mean	SD	EMM	95% CI	*n*	Mean	SD	EMM	95% CI	Δ	95% CI	*p*
FAS5	nmol/mL	Measured value	Scr	30	0.042	0.020	—	—	30	0.039	0.016	—	—	0.003	−0.006, 0.012	0.533
			12w	30	0.043	0.024	0.042	0.035, 0.048	30	0.047	0.018	0.048	0.042, 0.055	−0.006	−0.015, 0.003	0.183
		Amount of change	12w	30	0.001	0.019	0.001	−0.005, 0.008	30	0.008	0.019	0.007	0.001, 0.014	−0.006	−0.015, 0.003	0.183
	%	Rate of change	12w	30	6.8	39.5	8.9	−9.1, 26.9	30	37.1	67.0	35.0	17.0, 53.0	−26.1	−51.6, −0.6	0.045*
Males of FAS5	nmol/mL	Measured value	Scr	14	0.046	0.014	—	—	16	0.043	0.015	—	—	0.003	−0.008, 0.014	0.590
			12w	14	0.051	0.030	0.050	0.038, 0.062	16	0.052	0.020	0.053	0.042, 0.064	−0.003	−0.019, 0.013	0.714
		Amount of change	12w	14	0.005	0.022	0.005	−0.006, 0.017	16	0.009	0.021	0.008	−0.003, 0.020	−0.003	−0.019, 0.013	0.714
	%	Rate of change	12w	14	10.5	32.5	11.9	−15.6, 39.5	16	28.9	62.9	27.6	1.9, 53.4	−15.7	−53.5, 22.1	0.402
Females of FAS5	nmol/mL	Measured value	Scr	16	0.039	0.024	—	—	14	0.035	0.017	—	—	0.004	−0.011, 0.019	0.598
			12w	16	0.036	0.015	0.035	0.029, 0.040	14	0.042	0.014	0.043	0.037, 0.049	−0.008	−0.017, 0.000	0.051
		Amount of change	12w	16	−0.003	0.015	−0.002	−0.008, 0.003	14	0.007	0.017	0.006	0.000, 0.012	−0.008	−0.017, 0.000	0.051
	%	Rate of change	12w	16	3.6	45.6	6.7	−18.6, 32.0	14	46.4	72.6	42.8	15.8, 69.9	−36.1	−73.3, 1.0	0.056
Participants aged ≥ 40 years in FAS5	nmol/mL	Measured value	Scr	18	0.039	0.012	—	—	19	0.039	0.017	—	—	−0.001	−0.010, 0.009	0.914
			12w	18	0.037	0.011	0.037	0.030, 0.043	19	0.045	0.016	0.045	0.038, 0.051	−0.008	−0.017, 0.001	0.087
		Amount of change	12w	18	−0.002	0.012	−0.002	−0.008, 0.004	19	0.006	0.021	0.006	−0.001, 0.012	−0.008	−0.017, 0.001	0.087
	%	Rate of change	12w	18	−1.4	27.6	−2.2	−23.4, 19.1	19	38.5	78.3	39.2	18.5, 59.9	−41.4	−71.1, −11.7	0.008*
Participants aged < 40 years in FAS5	nmol/mL	Measured value	Scr	12	0.048	0.028	—	—	11	0.040	0.015	—	—	0.008	−0.012, 0.028	0.397
			12w	12	0.052	0.034	0.049	0.036, 0.062	11	0.052	0.022	0.055	0.042, 0.069	−0.006	−0.025, 0.012	0.484
		Amount of change	12w	12	0.004	0.026	0.005	−0.008, 0.018	11	0.012	0.014	0.011	−0.002, 0.025	−0.006	−0.025, 0.012	0.484
	%	Rate of change	12w	12	19.1	51.6	21.8	−6.5, 50.2	11	34.5	44.3	31.6	2.0, 61.2	−9.7	−51.1, 31.6	0.629
Participants whose score of Sleepiness on rising in the OSA‐MA above the median (14.15 point) at Scr in FAS5	nmol/mL	Measured value	Scr	15	0.046	0.021	—	—	16	0.042	0.018	—	—	0.004	−0.010, 0.018	0.564
			12w	15	0.044	0.015	0.043	0.035, 0.052	16	0.044	0.018	0.045	0.037, 0.053	−0.002	−0.013, 0.010	0.782
		Amount of change	12w	15	−0.002	0.017	−0.001	−0.009, 0.008	16	0.002	0.021	0.001	−0.007, 0.009	−0.002	−0.013, 0.010	0.782
	%	Rate of change	12w	15	0.6	29.1	3.7	−24.0, 31.3	16	23.3	76.0	20.4	−6.4, 47.2	−16.8	−55.4, 21.9	0.381
Participants whose score of Sleepiness on rising in OSA‐MA below the median (14.15 point) at Scr in FAS5	nmol/mL	Measured value	Scr	15	0.038	0.019	—	—	14	0.036	0.014	—	—	0.002	−0.010, 0.015	0.725
			12w	15	0.042	0.031	0.041	0.031, 0.050	14	0.051	0.018	0.052	0.042, 0.062	−0.011	−0.025, 0.002	0.098
		Amount of change	12w	15	0.003	0.021	0.003	−0.006, 0.013	14	0.014	0.013	0.015	0.005, 0.024	−0.011	−0.025, 0.002	0.098
	%	Rate of change	12w	15	12.9	48.0	14.2	−10.8, 39.3	14	52.8	53.3	51.4	25.5, 77.3	−37.2	−73.3, −1.1	0.044*

Abbreviations: 12w, 12 weeks after consumption; 95% CI, 95% confidence interval; Amount of change, amount of change from Scr; EMM, estimated marginal mean; FAS5, full analysis set5; OSA‐MA, OSA sleep inventory MA version; Rate of change, rate of change from Scr; Scr, screening (before consumption); SD, standard deviation; Δ, Difference between groups (BE group–placebo group).

* *p* < 0.05.

For TBARS, which demonstrated a significant difference in the overall analysis, the measured value and the amount and rate of change at 12w tended to be lower in the BE group than in the Placebo group only in the female participants of FAS5 regarding the effect of sex (*p* = 0.051, *p* = 0.051, *p* = 0.056, respectively; Table 9). As for the age analysis, the measured value and amount of change at 12w tended to be lower in the BE group than in the placebo group only in FAS5 participants and those aged ≥ 40 years (both *p* = 0.087), and the rate of change at 12w was significantly lower in the BE group than in the Placebo group (Table 9). Regarding sleepiness on rising in the OAS‐MA, the BE group recorded significantly lower measured values, and amounts of change at 12w tended to be lower in the BE group than in the Placebo group in FAS5 participants and those whose sleepiness on rising was below the median (14.15 points) at Scr (both *p* = 0.098), and the rate of change at 12w was lower than that of the Placebo group (Table 9).

### 3.10 Safety Evaluation

During the study period, three adverse events (10.0%) were observed in the BE group and two (6.5%) in the Placebo group (group difference: 3.5%; 95% CI: −10.2% to 17.3%). The adverse events reported by the three participants in the BE group were athlete's foot, headache, common cold, and sore throat, and the two participants in the Placebo group reported urticaria and acne. All symptoms were deemed by the study physician to be not causally related to the food intervention.

Moreover, urinalysis and blood test data were not significantly different between the groups (Appendix S8). Moreover, the study physician reviewed the safety assessment items (physical examination, urinalysis, and blood tests) by group (Appendices S9–S11) and by individuals (data not shown), and no medically relevant changes associated with the consumption of the test food were found.

## 4 Discussion

Proanthocyanidins and epicatechins are major constituents of polyphenols present in BE (Ito et al. 2013). Proanthocyanidins exert various effects, such as antioxidant activity (Ariga 2004). Epicatechins are a major component of catechins in green tea, and antioxidant effects are one of the physiological effects of catechins (Grzesik et al. 2018). In a randomized crossover study of 24 healthy Japanese volunteers who received BE (100 mg/day) or placebo for 4 weeks, the participants reported reduced fatigue as well as eye fatigue, sleepiness, and modulation of autonomic nervous system function upon consumption of BE contained food (Akagi et al. 2021). This study examined the effects of the consumption of the food containing BE on sleep quality in healthy individuals who were dissatisfied with their sleep quality.

This study enrolled healthy Japanese who complained of fatigue upon waking and poor sleep quality (short sleep time, difficulty falling asleep, difficulty sleeping soundly, having dreams, inability to get over fatigue, etc.) in daily life and had relatively low score on sleepiness on rising in the OSA‐MA at Scr (BE group, 14.2 ± 4.6; Placebo group, 14.5 ± 4.9, Table 3). Given that the score in sleepiness on rising in the OSA‐MA was 17.4 ± 7.0 on 284 healthy Japanese men and women aged 26–59 years (Yamamoto et al. 1999), the mean score of sleepiness on rising in healthy individuals is considered 17.4 points. The EMM and 95% CI of the score of sleepiness on rising at 12w were 18.6 points (95% CI, 17.0 to −20.1) for the BE group and 15.2 points (95% CI, 13.7–16.7) for the placebo group (Figure 2a), indicating that only the BE group had a mean score of sleepiness on rising higher than 17.4 points. In addition, the measured value and amount of change at 12w for “Question 2_I am concentrated–I am not concentrated,” at 8w and 12w for “Question 4_I am relaxed–I am stressed,” and at 12w for “Question 14_I can answer a survey quickly and easily right now–It's troublesome to answer,” which are related to sleepiness on rising of OSA‐MA, were significantly higher in the BE group than in the Placebo group (Table 4). Furthermore, the sleep test revealed that the change in the sleep‐onset latency was significantly lower in the BE group than in the Placebo group (Table 6). Previous studies of foods exhibiting antioxidant properties similar to BE confirmed that chlorogenic acid intake significantly improves fatigue upon awakening and sleep quality (Ochiai et al. 2018), and the combination of *Poria Cocos*, *Ziziphus spinose*, and gamma‐aminobutyric acid (GABA) intake significantly improves TST and sleep quality (Hao et al. 2024). Ochiai et al. (2018) showed that chlorogenic acid suppresses the decrease in parasympathetic activity during sleep by acting on the autonomic nervous system, indicating that it contributes to improving sleep quality. Because BE has also been confirmed to modulate autonomic nervous functions (Akagi et al. 2021), BE could exhibit a similar effect on improving sleep quality similar to chlorogenic acid. Our results supported these previous studies, indicating that BE intake decreased the latency to fall asleep and significantly improved sleepiness on rising.

In addition, the BE group had significantly lower levels of TBARS than the Placebo group in the rate of change from Scr (Table 9). TBARS increases in response to oxidative stress (De Aguilar Diaz Leon and Borges 2020). In a study of 24 healthy Japanese volunteers who received either BE (100 mg/day) or placebo for 2 weeks, TBARS significantly decreased with BE consumption (Akagi et al. 2024), and similar results were obtained in the present study. The brain produces endogenous bioactive substances with sleep‐inducing effects in response to oxidative stress reduction (Ikeda et al. 2005). Given that the major constituents of BE demonstrated antioxidant activities, the production of physiologically active substances with sleep‐inducing effects via the reduction of oxidative stress was thought to be responsible for the improvement in sleep quality.

Furthermore, the BE contained food was reported to increase the NO production capacity of vascular endothelial cells (Akagi et al. 2024). NO is a vasodilator that activates soluble guanylate cyclase (s‐GC) by binding to the heme moiety of s‐GC in vascular smooth muscles and induces an increase in the intracellular concentration of guanosine 3',5'‐cyclic monophosphate and a subsequent decrease in Ca^2+^ concentration (Carvajal et al. 2000). In a study in which healthy Japanese volunteers consumed BE (100 mg/day) or placebo for 2 weeks, urinary NO metabolites were significantly increased, and urinary levels of 8‐OhdG and TBARS (oxidative stress biomarkers) were significantly decreased (Akagi et al. 2024). The study also found that BE consumption significantly reduced vascular age and systolic blood pressure, and results from accelerated pulse wave measurements indicated improved vascular elasticity and intravascular pressure (Akagi et al. 2024). From these reports, BE might promote vasodilation and improve blood flow through an increase in NO production capacity; however, no significant differences in NO metabolites were identified in the present study (data not shown). In the present study, intragroup comparisons of the changes from Scr to 12w in the BE and Placebo groups, using a paired *t*‐test, did not reveal significant differences in the Placebo group; however, the BE group showed significantly higher values at 12w than at Scr (*p* = 0.043; data not shown).

Coldness of the hands and feet is a symptom of reduced terminal blood flow caused by the constriction of blood vessels in those areas (Hur et al. 2012; Traynor and MacDermid 2008; Yoshino et al. 2013). The AUC and the measured values of each measurement point, amount of change, and rate of change were significantly higher in the BE group than in the Placebo group (Figure 4, Appendix S6). The skin surface temperature of the fingertips fluctuates seasonally, and Gardner‐Medwin et al. (2001) revealed a 1°C–2°C difference in the skin surface temperature of the fingertips in a room during winter compared with summer (outdoor temperature; summer, approximately 19°C; winter, approximately 8°C; fingertip temperature; summer, approximately 33°C; winter, approximately 32°C). In the present study, the intervention period started August 28 and ended September 24, 2023, and the 12w examinations were conducted between November 20 and December 17, 2023. Monthly average temperatures of 2023 in Tokyo were 29.2°C in August and 26.2°C in September, compared with 14.4°C in November and 9.4°C in December, indicating a difference of 12°C–20°C between the two periods (Japan Meteorological Agency, n.d.). Therefore, the skin surface temperature was possibly affected by seasonal variations. When we examined the amount of change from Scr in the palmar surface temperature before the cold‐water load, a decrease was found in both groups: −0.44°C in the BE group and −1.77°C in the Placebo group (Appendix S6). However, only the BE group demonstrated less than the aforementioned seasonal variation (1°C–2°C), and significant inter‐group differences were observed, suggesting that the intake of BE contained food increased the palmar surface temperature with clinical significance.

The human body temperature and sleep are related; dorsal hand and foot skin temperatures rise relative to the trunk skin temperature before sleep, and the magnitude of this rise is positively correlated with sleepiness (Kräuchi et al. 1999). In fact, bathing with transient increases in skin and deep body temperatures stimulates the temperature regulatory center and subsequently increases the blood flow in peripheral blood vessels of the skin, resulting in increased heat dissipation and promoting sleep (Maeda et al. 2023). Therefore, the BE may have promoted vasodilation and increased the skin temperature through an increase in NO production capacity, thereby decreasing the sleep‐onset latency and significantly improving sleepiness on rising.

“Health Japan 21 (the third term)” aims to increase the percentage of people who are well rested from sleep, and its indicator is regarded as their sense of restfulness from sleep (Ministry of Health Labour and Welfare 2023). This study also evaluated sleep introspection upon waking, and improvement in sleepiness on rising was confirmed, indicating that the intake of BE may have increased the sense of sleep restfulness as defined by Health Japan 21 (the third term). Therefore, BE‐containing foods were considered to contribute to “increasing the percentage of people who are well rested from sleep,” one of the goals of Health Japan 21 (the third term).

In this study, no significant difference in NO metabolite production was found between the BE and placebo groups. In a study investigating the relationship between smoking habits and NO production, the production rate of NOX, an NO metabolite, was significantly lower in smokers than in nonsmokers (Grassi et al. 2010). In addition, a study of 30 men in their 30s–40s who consumed green tea containing 0, 80, or 580 mg of catechins for 2 weeks revealed a significant increase in NOX levels in the group consuming 580 mg catechins compared with before consumption (Oyama et al. 2010). In a study estimating the daily intake of major catechins in 146 participants who most frequently drink green tea at home, catechin intake varied from a mean of 481.0 mg to a maximum of 2722.9 mg and a minimum of 0 mg (Takahashi et al. 2004). In the present study, an exclusion criterion was the prohibition of excessive alcohol consumption and intake of foods and beverages containing ingredients thought to affect sleep. However, smoking habits and intake of catechins, which may affect NO metabolism related to the mechanism of action of BE, were not considered as factors. In the future study, analysis of these factors will allow us to confirm the effects of food intake on the amount of NO metabolites and more comprehensively examine the effects of BE on sleep quality.

“Stiff shoulders” and “eye fatigue” in the original questionnaire were significantly improved in the Placebo group compared with that in the BE group at several evaluation points after the intervention (Appendix S4). However, this study enrolled healthy individuals who complained of fatigue upon waking and poor sleep quality (short sleep time, difficulty falling asleep, difficulty sleeping soundly, having dreams, inability to get over fatigue, etc.) and did not consider the severity of stiff shoulders and eye fatigue at Scr. Therefore, differences in the degree of symptoms of stiff shoulders and eye fatigue at Scr may have affected the study results.

Subgroup analyses were conducted to examine more comprehensively the effect of BE intake on sleep quality. First, given the sex differences in sleep quality (Mander et al. 2017), male and female subgroups were constructed to ascertain the effect of sex on sleep quality. In the male subgroup, measured values and changes in the score on sleepiness on rising in OSA‐MA were significantly higher in the BE group than in the Placebo group (Figure 3a). In addition, “Question 2_I am concentrated–I am not concentrated,” “Question 4_I am relaxed–I am stressed,” “Question 8_I feel clearheaded–I feel foggy headed,” and “Question 14_I can answer a survey quickly and easily right now–It's troublesome to answer” (Japan Organization of Better Sleep, n.d.) comprise sleepiness on rising. Significant differences were observed in questions 2, 8, and 14 for the male subgroup (Table 5), with the BE group having higher values than the Placebo group. The BE contained food was expected to improve the sleepiness on rising by increasing skin temperature through increased NO production; indeed, a significant increase in the palmar surface temperature before the cold‐water load was observed in the BE group (Figure 4b), indicating that BE may improve sleepiness through the expected mechanism in men.

In contrast, no significant differences were observed in sleepiness on rising among the female participants (Figure 3b), although the score on question 4 of OSA‐MA was significantly higher (Table 5). However, an increasing trend was observed in sleep scores in the sleep test (*p* = 0.071, data not shown), and TST was significantly higher in the BE group than in the Placebo group (Table 7). A goal of “Health Japan 21 (the third term)” related to rest and sleep is to increase the number of people who get enough sleep (6–9 h of sleep) (Ministry of Health Labour and Welfare 2023). Women tend to sleep shorter hours than men, and in this study, the TST for female participants showed a mean of 19,471.5 s (approximately 5.4 h) at Scr in the BE group and 20,551.1 s (approximately 5.7 h) in the Placebo group. After the intervention, in female participants, the EMM at 12w was 21,837.8 s (approximately 6.1 h) in the BE group and 19,028.8 s (approximately 5.3 h) in the Placebo group (Table 7), indicating that the 6‐h (21,600 s) goal of Health Japan 21 (the third term) was achieved only in the BE group (Table 7). According to the results of a survey of 5701 Japanese (2668 men and 3033 women) aged ≥ 20 years on average daily sleep time (Ministry of Health Labor and Welfare 2019), 37.5% of men and 40.6% of women reported sleeping < 6 h, indicating that a significant increase in the sleep time was considered a meaningful change for women. Thus, the BE contained food was effective in decreasing the sleepiness on rising for men and increasing the sleep duration for women.

In participants aged ≥ 40 years, who were organized to examine the effect of aging on sleep quality, the BE group had a significantly higher score on questions 8 and 14 of OSA‐MA, which are the components of sleepiness on rising, than the Placebo group (Table 5). In the palmar surface temperature measurement, the AUC and palmar surface temperatures at all measurement time points from before and immediately after the cold‐water load to 30 min after the cold‐water load were significantly higher in the BE group than in the placebo group (Figure 4c). These results reflect that BE may have improved sleep quality via increased skin surface temperature. In addition, the rate of change in TBARS, an oxidative stress marker, was significantly lower in the BE group than in the Placebo group (Table 9), and this result supported previous data (Ariga 2004; Namba 2018; Wang et al. 1997; Yoshioka et al. 2017). An age‐related decrease in saliva production in middle‐aged and older adults (Smith et al. 2013; Xu et al. 2019) increases the number of individuals complaining of dry mouth (xerostomia) (Ohara et al. 2016). Dry mouth symptoms include poor sleep quality associated with nocturnal dry mouth sensations (Burgess 2014). Owing to the decline in sleep quality in the PSQI in patients with dry mouth (Lopez‐Jornet et al. 2016), alleviating dry mouth may be important for improving sleep quality. In this study, the original questionnaire showed a significantly lower rate of change in dry mouth in the BE group than in the Placebo group (Appendix S5). Because the water component of saliva is supplied from the plasma in blood vessels, saliva secretion requires increased blood flow in the salivary glands (Izumi and Karita 1994; Mizuta et al. 2000). In the original questionnaire, “dry mouth” was thought to have decreased because of the enhanced saliva secretion via blood flow, which promoted the effect of BE contained in the test food.

Menopausal symptoms include insomnia (Terauchi et al. 2012) and dry mouth (Meurman et al. 2009). Premenopausal women have significantly more volume of saliva in non‐stimulation situations than postmenopausal women (Streckfus et al. 1998). A study evaluating factors that affect sleep quality in women aged 35–49 years found significant associations with anxiety, depression, and caffeine intake in the overall population. This study reported that lower estradiol levels were significantly associated with lower sleep quality in women aged ≥ 45 years (Hollander et al. 2001). In this study, no significant difference in sleep quality and dry mouth was noted in the subgroup of women of all ages, whereas a significant difference in sleep quality and dry mouth was observed in the subgroup of those aged ≥ 40 years. The decrease in female hormones associated with menopause was assumed to affect sleep quality and dry mouth; indeed, women aged < 40 years did not exhibit significant change in those parameters (data not shown). Thus, BE may be useful for menopausal women. However, given the small number of participants, this hypothesis needs to be verified in the future.

Furthermore, in a subgroup of participants whose scores in sleepiness on rising in OSA‐MA were below the median (14.15 points) at Scr, sleepiness on rising in OSA‐MA was significantly higher at 8w (*p* = 0.038) and showed an increasing trend at 12w (*p* = 0.060) in the BE group than in the placebo group (Figure 3f). For the palmar surface temperature measurement, the AUC and the actual values and changes at 12w in the palmar surface temperature before cold‐water load in the BE group were significantly higher compared with the placebo group (Figure 4d). In the sleep test, N3 total time at 12w was significantly higher in the BE group than in the Placebo group (Table 8). In humans, sleep consists of a cycle of alternating REM and non‐REM sleep lasting approximately 80–100 min each (Carskadon and Dement 2011). Stage N3, deep sleep, often appears in the first half of sleep. However, N3 decreases with age, accompanied by an increase in mid‐wake and shallow sleep (Carskadon and Dement 2011). Furthermore, N3 is more significantly reduced with 0% in serious cases of sleep apnea, whereas the ratio of N3 to sleep time was 20% in the 20s and 5% in the 60s (Geyer and Carney 2018). Given these reports, N3 (N3 total time) likely decreased in this subgroup analysis, as it included participants who were presumed to have poor sleep quality. The mean of N3 total time at Scr of all the participants of FAS4 was 2622.8 s (data not shown). In the subgroup with sleepiness on rising below the median, it was 2171.8 s in the BE group and 2092.0 s in the Placebo group (Table 8), indicating that the subgroup with sleepiness on rising below the median had less N3 total time. These results indicate that the N3 total time was more sensitive to sleep quality improvement associated with the intake of BE contained food by those with poor sleep quality. In addition, the rate of change in “dry mouth” at 12w was significantly lower in the BE group than in the Placebo group (Appendix S5). Exacerbated thirst causes awakening for water intake (Lavigne et al. 1999; Thie et al. 2002) and decreases sleep quality. BE increased heat dissipation in the skin (Maeda et al. 2023) and saliva secretion (Izumi and Karita 1994; Mizuta et al. 2000) by increasing the blood flow in peripheral skin blood vessels and improving the sleep quality of those with poor sleep quality with increasing sleepiness on rising and N3 total time, a deep sleep indicator.

In this study, two reasons were considered for the lack of significant differences in the blood flow despite significant differences in palm skin surface temperature: (1) differences in the measurement site and (2) the detection limit of the measurement device. Regarding the first reason, the palmar surface temperature was evaluated at the palm area, whereas the blood flow rate was measured on the entire hand (from the wrist to the fingertips), and one site was selected from the back of the hand for analysis. The skin surface temperature is different among regions of the body. In a study by Nishihara and Hasebe (2003), eight healthy female students (aged 22.1 ± 0.6 years) were exposed to cold air on the chest, thorax, mid‐back, upper wrist, anterior wrist, thigh, and lower leg, and the skin surface temperatures in the chest, thorax, mid‐back, upper wrist, anterior wrist, thigh, lower leg, forehead, belly, back of hands, fingers first, back of feet, and toes were investigated. The maximum and minimum skin surface temperature changes after 40 min of cold exposure were −0.52°C and −0.87°C, respectively, for the back of the hands (Nishihara and Hasebe 2003). Although the measurement site in the study of Nishihara and Hasebe (2003) was the hand dorsum, which was different from the measurement site in the present study, the difference in the measurement body site may have affected the skin surface temperature and blood flow in this study. Regarding the second reason, that is, the detection limit of the measurement device, a laser speckle blood flowmeter was used in this study. When a phase‐aligned light such as laser light is irradiated onto a rough surface, a speckle pattern (grainy pattern) is observed (Briers 2007). When a laser beam is irradiated onto a non‐moving object, the speckle pattern does not change, whereas when a laser beam is irradiated onto a moving object, the speckle pattern changes dependently on the object's moving speed (OMEGAWAVE Inc., n.d.). A laser speckle blood flowmeter calculates blood flow based on the speckle pattern generated by a laser beam irradiating the skin and reflecting off red blood cells (OMEGAWAVE Inc., n.d.) and can measure blood flow in the skin to a depth of ≤ 1 mm (Kashima 2003). In contrast, a laser Doppler blood flowmeter calculates blood flow from the product of red blood cell count and blood flow velocity using the frequency change (Doppler effect) when a laser beam strikes an object (mainly red blood cells) moving in a capillary and can measure blood flow in capillaries at a depth of approximately 0.5 mm from the skin surface (Clough et al. 2009). Although the principles of laser speckle and laser Doppler blood flowmetries slightly vary, the measurement results are reported to be generally comparable (Briers 1996). The average of multiple measurements must be used for quantification by laser Doppler blood flowmetry (Bray et al. 2003). As mentioned above, in this study, blood flow measurements were performed on the entire hand from the wrist to the fingertips, and the analysis was performed using the average value within a circle set up to include most of the hand dorsum. Although no publications have recommended multiple measurement points for laser speckle blood flowmetry, analyses of only one point did not reflect blood flow changes caused by the intake of the BE contained food. Furthermore, because the measurement depth of the laser speckle blood flowmeter is limited to 1 mm (Kashima 2003), even the analyzed points may not reflect blood flow changes in the deeper regions. For these two reasons, blood flow changes associated with the intake of BE contained food were not observed in this study.

One of the limitations of our study is that we cannot completely rule out that body weight and body composition may affect BE absorption. The major polyphenols in BE are epicatechin, procyanidin B2, procyanidin C1, and cinnamtannin A2 (Table 1). In a study examining differences in the physiokinetics of catechins between normal and obese rats, obese rats showed significantly higher concentrations of epicatechin in small intestinal contents and feces than normal rats, indicating that epicatechin entering the body is reduced in obese rats (Liu et al. 2024). Thus, BE absorption may be different in humans depending on the degree of obesity. However, no clinical trials were conducted regarding the effect of obesity on BE absorption. Therefore, investigating whether the degree of obesity affects the absorption of BE in humans should be addressed in future studies.

## 5 Conclusions

This study examined the effect of the consumption of BE on sleep quality in healthy Japanese adults who were dissatisfied with their sleep quality. The primary outcome, sleepiness on rising in OSA‐MA at 12w, was significantly higher in the BE group than in the Placebo group, indicating that the intake of BE contained food significantly improved sleep quality. Compared with the Placebo group, the BE group had significantly shorter sleep‐onset latency, relieved dry mouth sensations, and increased palmar surface temperature. The subgroup analyses showed that BE effectively decreased sleepiness on rising for men and increased the sleep time for women. Moreover, participants aged ≥ 40 years had improved age‐related decline in sleep quality and dry mouth, and participants with poor sleep quality had reduced sleepiness on rising and increased deep sleep. The consumption of BE was safe under the conditions of this study.

## Author Contributions

Conceptualization, R.A., T.M., and K.M.; Methodology, R.A. and T.M.; Formal Analysis, R.A. and T.M.; Investigation, T.T.; Resources, R.A. and T.M.; Data Curation, R.A. and T.M.; Writing – Original Draft Preparation, R.A. and T.M.; Writing – Review and Editing, R.A., T.M., T.T., and K.M.; Visualization, R.A. and T.M.; Supervision, T.T.; Project Administration, R.A. and T.M.

## Ethics Statement

This study was approved by the ethics committee of the Takara Clinic, Medical Corporation Seishinkai on May 17, 2023 (Approval ID: 2305‐00060‐0055‐16‐TC). The study was completed following the guidelines stipulated in the latest Declaration of Helsinki and Ethical Guidelines for Medical and Biological Research Involving Human Subjects in Japan.

## Consent

Written informed consent was obtained from all study participants.

## Conflicts of Interest

R.A., T.M., and K.M. are employees of Fujicco Co. Ltd. T.T. has no competing interests to declare that are relevant to the content of this article.

## Supporting information


Appendix S1–S11.

## Data Availability

The authors have nothing to report.
