# Supplementary material for: Effects of Consumption of Black Soybean Seed Coat Extract on Sleep Quality in Healthy Japanese: A Randomized, Placebo‐Controlled, Double‐Blind, Parallel‐Group Comparison Study
Source: Food Sci Nutr. 2025 Jun 11;13(6):e70156. doi: 10.1002/fsn3.70156 (PMC12153013; doi:10.1002/fsn3.70156)
Supplement: Supplementary file 1 — Appendix S1–S11. [file FSN3-13-e70156-s001.docx]

|  |  |  |  |
| --- | --- | --- | --- |
|  | Appendix 1. Procedure of cold-water load^a^ | |  |
|  | Steps | Details |  |
|  | 1 | Wear vinyl gloves (rubber gloves) to keep both hands dry, with the backs of the hands up and fingers slightly open (relaxed). |  |
|  | 2 | Place the hands as described in (1) up to the wrists in a water bath filled with cold water at a temperature of 15°C not to get wet. When the hands are slightly floating above the bottom of the bath, cool them for 1 min while moving them slowly back and forth (about 60 round trips/min). |  |
|  | 3 | Immediately after drawing the hands out of the water, remove the vinyl gloves and place the hands on a platform in a sitting position with the elbows bent at about 90° and the shoulders relaxed, with the backs of the hands facing up. |  |
|  | 4 | The post-load measurement is conducted 5 times, with the first measurement under the condition of (3) set at 0 min, followed by 5, 10, 20, and 30 min later. |  |
|  | ^a^The loading was conducted after the participants were at rest for at least 30 min in a room with a constant temperature and humidity. | |  |
|  |  |  |  |

|  |  |  |  |  |  |  |
| --- | --- | --- | --- | --- | --- | --- |
|  | Appendix 2. List of participants with missing data | | | |  |  |
|  | ID | Date of untraceability | Time point | Absence of missing data | Missing variables | Reasons for lack of measurement |
|  | Participant 1 | 2023-9-27 | Scr | - | - | - |
|  |  |  | 4w | - | - | - |
|  |  |  | 8w | missing | All variables defined in the protocol | Rejection to receiving the examination for personal reasons |
|  |  |  | 12w | missing | All variables defined in the protocol | Rejection to receiving the examination for personal reasons |
|  | Participant 2 | 2023-11-7 | Scr | - | - | - |
|  |  |  | 4w | - | - | - |
|  |  |  | 8w | - | - | - |
|  |  |  | 12w | missing | All variables defined in the protocol | Rejection to receiving the examination for personal reasons |
|  | Participant 3 | 2024-11-12 | Scr | - | - | - |
|  |  |  | 4w | - | - | - |
|  |  |  | 8w | - | - | - |
|  |  |  | 12w | missing | All variables defined in the protocol | Rejection to receiving the examination for personal reasons |
|  | Participant 4 | - | Scr | - | - | - |
|  |  |  | 4w | - | - | - |
|  |  |  | 8w | - | - | - |
|  |  |  | 12w | missing | All variables obtained from the sleep test (using InSomnograf®) | Mistakes in the operation of the instruments by the participant himself/herself |
|  | Participant 5 | - | Scr | - | - | - |
|  |  |  | 4w | - | - | - |
|  |  |  | 8w | - | - | - |
|  |  |  | 12w | missing | All variables obtained from skin surface temperature tests (using thermography) | Malfunction of inspection equipment |
|  | Participant 6 | - | Scr | - | - | - |
|  |  |  | 4w | - | - | - |
|  |  |  | 8w | - | - | - |
|  |  |  | 12w | missing | All variables obtained from blood tests (performed at Fujicco Co. Ltd.) | Refusal of blood collection |
|  | Scr, screening (baseline); 4w, 4 weeks after consumption; 8w, 8 weeks after consumption; 12w, 12 weeks after consumption | | | | | |
|  |  |  |  |  |  |  |

|  | Appendix 3. Llist of excluded participants for each analysis dataset | | | |  |
| --- | --- | --- | --- | --- | --- |
|  | Analysis dataset | Analysis variables | Groups | Participants | Reasons for exclusion |
|  | ITT | - | - | - | - |
|  | FAS1 | OSA-MA, VAS | - | - | - |
|  | FAS2 | PSQI-J, POMS2, Blood flow test | Placebo group | Participant 3 | No data at all after allocation |
|  |  |  | BE group | Participant 1 | No data at all after allocation |
|  |  |  | BE group | Participant 2 | No data at all after allocation |
|  | FAS3 | Skin surface temperature test | Placebo group | Participant 3 | No data at all after allocation |
|  |  |  | BE group | Participant 1 | No data at all after allocation |
|  |  |  | BE group | Participant 2 | No data at all after allocation |
|  |  |  | BE group | Participant 5 | No data at all after allocation |
|  | FAS4 | Sleep test | Placebo group | Participant 3 | No data at all after allocation |
|  |  |  | BE group | Participant 1 | No data at all after allocation |
|  |  |  | BE group | Participant 2 | No data at all after allocation |
|  |  |  | BE group | Participant 4 | No data at all after allocation |
|  | FAS5 | Blood test (performed at Fujicco Co. Ltd.) | Placebo group | Participant 3 | No data at all after allocation |
|  |  |  | Placebo group | Participant 6 | No data at all after allocation |
|  |  |  | BE group | Participant 1 | No data at all after allocation |
|  |  |  | BE group | Participant 2 | No data at all after allocation |
|  | SAF | Safety evaluation | Placebo group | Participant 3 | No data at all after allocation |
|  |  |  | BE group | Participant 1 | No data at all after allocation |
|  |  |  | BE group | Participant 2 | No data at all after allocation |
|  |  |  |  |  |  |

Appendix 4. Comparison of subjective symptoms in visual analogue scale of subjective symptoms (FAS1)

| Items | Unit | Classification | Time point | BE group | | | |  | Placebo group | | | |  | Group comparison | |
| --- | --- | --- | --- | --- | --- | --- | --- | --- | --- | --- | --- | --- | --- | --- | --- |
|  |  |  |  | n | Mean | SD | EMM (95%CI) |  | n | Mean | SD | EMM (95%CI) |  | Δ (95%CI) | P value |
| Stiff shoulders | mm | Measured value | Scr | 32 | 45.3 | 27.1 | - |  | 32 | 46.9 | 23.8 | - |  | –1.6 (–14.3, 11.2) | 0.807 |
|  |  |  | 4w | 32 | 53.7 | 21.0 | 54.4 (47.2, 61.5) |  | 32 | 44.0 | 25.2 | 44.0 (36.8, 51.2) |  | 10.4 (0.2, 20.5) | 0.046* |
|  |  |  | 8w | 31 | 47.5 | 20.8 | 47.4 (40.2, 54.7) |  | 32 | 33.2 | 23.7 | 33.1 (26.0, 40.3) |  | 14.3 (4.1, 24.5) | 0.007* |
|  |  |  | 12w | 30 | 47.4 | 24.2 | 46.6 (40.2, 53.1) |  | 31 | 32.4 | 19.0 | 33.0 (26.6, 39.4) |  | 13.6 (4.6, 22.7) | 0.004* |
|  |  | Amount of change | 4w | 32 | 8.4 | 21.5 | 7.6 (0.4, 14.8) |  | 32 | –2.8 | 27.1 | –2.8 (–10.0, 4.4) |  | 10.4 (0.2, 20.5) | 0.046* |
|  |  |  | 8w | 31 | 0.9 | 22.0 | 0.6 (–6.6, 7.9) |  | 32 | –13.7 | 28.6 | –13.6 (–20.8, –6.5) |  | 14.3 (4.1, 24.5) | 0.007* |
|  |  |  | 12w | 30 | –0.4 | 21.8 | –0.2 (–6.6, 6.3) |  | 31 | –14.9 | 20.7 | –13.8 (–20.1, –7.4) |  | 13.6 (4.6, 22.7) | 0.004* |
|  |  | Rate of change | 4w | 32 | 135.1 | 387.5 | 126.4 (36.4, 216.4) |  | 32 | 41.6 | 153.6 | 42.2 (–47.7, 132.1) |  | 84.2 (–43.0, 211.5) | 0.191 |
|  |  |  | 8w | 31 | 115.6 | 474.9 | 111.2 (–0.1, 222.5) |  | 32 | 1.2 | 105.0 | 1.7 (–109.3, 112.6) |  | 109.5 (–47.6, 266.7) | 0.168 |
|  |  |  | 12w | 30 | 167.7 | 858.3 | 143.7 (–62.1, 349.4) |  | 31 | –10.0 | 87.9 | –3.8 (–208.4, 200.8) |  | 147.4 (–142.7, 437.6) | 0.314 |
| Eye fatigue | mm | Measured value | Scr | 32 | 44.2 | 21.1 | - |  | 32 | 44.0 | 19.5 | - |  | 0.2 (–10.0, 10.4) | 0.971 |
|  |  |  | 4w | 32 | 46.3 | 22.8 | 46.4 (39.0, 53.7) |  | 32 | 37.5 | 19.9 | 37.6 (30.3, 45.0) |  | 8.7 (–1.6, 19.1) | 0.098 |
|  |  |  | 8w | 31 | 44.8 | 14.2 | 44.4 (38.4, 50.5) |  | 32 | 33.8 | 19.7 | 33.9 (27.9, 39.8) |  | 10.6 (2.1, 19.1) | 0.016* |
|  |  |  | 12w | 30 | 36.9 | 20.6 | 36.7 (30.1, 43.3) |  | 31 | 33.2 | 17.9 | 33.9 (27.4, 40.4) |  | 2.8 (–6.5, 12.1) | 0.554 |
|  |  | Amount of change | 4w | 32 | 2.1 | 23.8 | 2.0 (–5.3, 9.3) |  | 32 | –6.5 | 26.5 | –6.7 (–14.1, 0.6) |  | 8.7 (–1.6, 19.1) | 0.098 |
|  |  |  | 8w | 31 | –0.6 | 20.8 | 0.1 (–6.0, 6.1) |  | 32 | –10.2 | 25.6 | –10.5 (–16.5, –4.5) |  | 10.6 (2.1, 19.1) | 0.016* |
|  |  |  | 12w | 30 | –7.6 | 21.0 | –7.7 (–14.3, –1.1) |  | 31 | –10.9 | 23.2 | –10.5 (–17.0, –3.9) |  | 2.8 (–6.5, 12.1) | 0.554 |
|  |  | Rate of change | 4w | 32 | 89.0 | 323.7 | 87.5 (9.2, 165.8) |  | 32 | 53.1 | 222.5 | 50.0 (–28.3, 128.3) |  | 37.5 (–73.2, 148.3) | 0.501 |
|  |  |  | 8w | 31 | 120.6 | 529.9 | 125.5 (5.4, 245.6) |  | 32 | 26.7 | 172.1 | 23.0 (–96.6, 142.6) |  | 102.5 (–67.0, 271.9) | 0.231 |
|  |  |  | 12w | 30 | 151.4 | 898.5 | 150.5 (–59.3, 360.3) |  | 31 | 29.6 | 197.6 | 27.9 (–180.6, 236.4) |  | 122.6 (–173.2, 418.4) | 0.410 |
| Dry mouth | mm | Measured value | Scr | 32 | 42.1 | 23.0 | - |  | 32 | 39.8 | 23.2 | - |  | 2.3 (–9.3, 13.8) | 0.698 |
|  |  |  | 4w | 32 | 34.5 | 21.1 | 34.3 (27.5, 41.1) |  | 32 | 33.9 | 20.3 | 34.5 (27.7, 41.3) |  | –0.2 (–9.8, 9.4) | 0.968 |
|  |  |  | 8w | 31 | 35.3 | 19.0 | 34.6 (28.2, 41.0) |  | 32 | 31.3 | 18.6 | 31.7 (25.4, 38.0) |  | 2.9 (–6.1, 11.9) | 0.523 |
|  |  |  | 12w | 30 | 31.3 | 21.3 | 30.0 (22.8, 37.3) |  | 31 | 39.4 | 21.8 | 40.5 (33.3, 47.6) |  | –10.4 (–20.6, –0.2) | 0.045* |
|  |  | Amount of change | 4w | 32 | –7.5 | 21.8 | –7.1 (–13.8, –0.3) |  | 32 | -5.9 | 26.5 | –6.9 (–13.7, –0.1) |  | –0.2 (–9.8, 9.4) | 0.968 |
|  |  |  | 8w | 31 | –8.0 | 22.9 | –6.7 (–13.1, –0.3) |  | 32 | -8.5 | 25.8 | –9.6 (–16.0, –3.3) |  | 2.9 (–6.1, 11.9) | 0.523 |
|  |  |  | 12w | 30 | –11.2 | 16.4 | –11.3 (–18.6, –4.0) |  | 31 | -1.2 | 29.9 | –0.9 (–8.0, 6.3) |  | –10.4 (–20.6, –0.2) | 0.045* |
|  |  | Rate of change | 4w | 32 | 28.1 | 177.7 | 31.9 (–34.9, 98.7) |  | 32 | 70.3 | 258.7 | 62.5 (–4.4, 129.4) |  | –30.6 (–125.2, 64.0) | 0.520 |
|  |  |  | 8w | 31 | 38.8 | 216.1 | 44.7 (–6.4, 95.7) |  | 32 | 26.6 | 117.0 | 20.1 (–30.4, 70.7) |  | 24.5 (–47.4, 96.5) | 0.498 |
|  |  |  | 12w | 30 | –20.0 | 69.1 | –16.6 (–76.1, 42.9) |  | 31 | 87.7 | 252.6 | 79.2 (20.3, 138.1) |  | –95.8 (–179.6, –12.0) | 0.026* |

FAS1, full analysis set1; SD, standard deviation; EMM, estimated marginal mean; Δ, Difference between groups (BE group - placebo group); 95%CI, 95% confidence interval; Scr, screening (before consumption); 4w, 4 weeks after consumption; 8w, 8 weeks after consumption; 12w, 12 weeks after consumption; Amount of change, amount of change from Scr; Rate of change, rate of change from Scr. **P* < 0.05

Appendix 5. Effect of age and sleepiness on rising on subjective dry mouth

| Analysis set | Items | Unit | Classification | Time point | BE group | | | |  | Placebo group | | | |  | Group comparison | |
| --- | --- | --- | --- | --- | --- | --- | --- | --- | --- | --- | --- | --- | --- | --- | --- | --- |
|  |  |  |  |  | n | Mean | SD | EMM (95%CI) |  | n | Mean | SD | EMM (95%CI) |  | Δ (95%CI) | P value |
| Participants aged ≥40 years in FAS1 | Dry mouth | mm | Measured value | Scr | 20 | 42.7 | 23.9 | - |  | 20 | 45.5 | 22.5 | - |  | –2.8 (–17.7, 12.1) | 0.705 |
|  |  |  |  | 4w | 20 | 30.9 | 22.8 | 31.5 (21.8, 41.2) |  | 20 | 37.0 | 22.1 | 36.6 (27.0, 46.3) |  | –5.2 (–18.9, 8.6) | 0.451 |
|  |  |  |  | 8w | 19 | 33.4 | 22.3 | 33.1 (24.5, 41.8) |  | 20 | 29.3 | 16.0 | 29.1 (20.6, 37.5) |  | 4.1 (–8.0, 16.1) | 0.502 |
|  |  |  |  | 12w | 18 | 28.9 | 22.7 | 28.3 (18.6, 38.0) |  | 20 | 39.5 | 20.1 | 39.2 (29.8, 48.6) |  | –11.0 (–24.5, 2.5) | 0.109 |
|  |  |  | Amount of change | 4w | 20 | –11.8 | 22.0 | –13.1 (–22.8, –3.3) |  | 20 | –8.5 | 29.8 | –7.9 (–17.6, 1.8) |  | –5.2 (–18.9, 8.6) | 0.451 |
|  |  |  |  | 8w | 19 | –11.3 | 24.4 | –11.4 (–20.1, –2.8) |  | 20 | –16.2 | 25.8 | –15.5 (–23.9, –7.0) |  | 4.1 (–8.0, 16.1) | 0.502 |
|  |  |  |  | 12w | 18 | –14.6 | 17.4 | –16.3 (–26.0, –6.6) |  | 20 | –6.0 | 33.0 | –5.3 (–14.7, 4.1) |  | –11.0 (–24.5, 2.5) | 0.109 |
|  |  | % | Rate of change | 4w | 20 | –19.7 | 53.3 | –26.4 (–91.8, 39.1) |  | 20 | 53.4 | 226.1 | 56.6 (–8.8, 122.0) |  | –83.0 (–175.6, 9.6) | 0.078 |
|  |  |  |  | 8w | 19 | –15.5 | 66.9 | –16.5 (–51.5, 18.6) |  | 20 | 5.7 | 113.3 | 7.9 (–26.9, 42.6) |  | –24.3 (–73.7, 25.1) | 0.325 |
|  |  |  |  | 12w | 18 | –32.4 | 47.4 | –37.0 (–119.7, 45.8) |  | 20 | 82.5 | 286.7 | 86.7 (4.9, 168.5) |  | –123.6 (–240.1, –7.2) | 0.038* |
| Participants aged <40 years in FAS1 | Dry mouth | mm | Measured value | Scr | 12 | 41.1 | 22.4 | - |  | 12 | 30.4 | 22.2 | - |  | 10.7 (–8.2, 29.6) | 0.254 |
|  |  |  |  | 4w | 12 | 40.7 | 16.8 | 38.8 (29.7, 48.0) |  | 12 | 28.9 | 16.5 | 30.9 (21.8, 40.1) |  | 7.9 (–5.2, 21.0) | 0.223 |
|  |  |  |  | 8w | 12 | 38.3 | 12.5 | 36.3 (26.4, 46.1) |  | 12 | 34.6 | 22.6 | 36.9 (27.0, 46.8) |  | –0.6 (–14.8, 13.5) | 0.929 |
|  |  |  |  | 12w | 12 | 34.8 | 19.4 | 31.7 (20.5, 42.8) |  | 11 | 39.4 | 25.6 | 44.8 (33.5, 56.0) |  | –13.1 (–29.2, 3.0) | 0.105 |
|  |  |  | Amount of change | 4w | 12 | –0.4 | 20.3 | 2.8 (–6.3, 11.9) |  | 12 | –1.5 | 20.5 | –5.1 (–14.2, 4.0) |  | 7.9 (–5.2, 21.0) | 0.223 |
|  |  |  |  | 8w | 12 | –2.8 | 20.1 | 0.2 (–9.6, 10.1) |  | 12 | 4.2 | 21.1 | 0.9 (–9.0, 10.7) |  | –0.6 (–14.8, 13.5) | 0.929 |
|  |  |  |  | 12w | 12 | –6.3 | 13.9 | –4.4 (–15.5, 6.8) |  | 11 | 7.6 | 22.2 | 8.7 (–2.6, 20.0) |  | –13.1 (–29.2, 3.0) | 0.105 |
|  |  | % | Rate of change | 4w | 12 | 107.8 | 270.2 | 149.9 (8.8, 291.1) |  | 12 | 98.4 | 314.5 | 51.6 (–90.0, 193.2) |  | 98.4 (–104.5, 301.3) | 0.325 |
|  |  |  |  | 8w | 12 | 124.9 | 327.0 | 162.9 (50.6, 275.2) |  | 12 | 61.3 | 119.5 | 19.2 (–93.5, 131.8) |  | 143.7 (–17.7, 305.2) | 0.078 |
|  |  |  |  | 12w | 12 | –1.4 | 92.2 | 12.9 (–68.7, 94.6) |  | 11 | 97.0 | 187.5 | 74.2 (–9.4, 157.7) |  | –61.2 (–179.6, 57.1) | 0.293 |
| Participants whose score of Sleepiness on rising in the OSA-MA above the median (14.15 point) at Scr in FAS1 | Dry mouth | mm | Measured value | Scr | 16 | 29.5 | 17.1 | - |  | 16 | 32.2 | 21.0 | - |  | –2.7 (–16.5, 11.2) | 0.694 |
|  |  |  |  | 4w | 16 | 34.3 | 20.1 | 34.8 (24.7, 44.8) |  | 16 | 35.3 | 19.7 | 35.1 (25.1, 45.1) |  | –0.3 (–14.5, 13.9) | 0.963 |
|  |  |  |  | 8w | 15 | 34.7 | 15.7 | 34.3 (26.9, 41.8) |  | 16 | 24.8 | 12.9 | 24.6 (17.4, 31.9) |  | 9.7 (–0.7, 20.1) | 0.065 |
|  |  |  |  | 12w | 15 | 25.9 | 13.5 | 25.3 (16.4, 34.2) |  | 16 | 34.9 | 19.2 | 34.9 (26.2, 43.6) |  | –9.6 (–22.1, 2.9) | 0.126 |
|  |  |  | Amount of change | 4w | 16 | 4.8 | 18.4 | 3.4 (–6.7, 13.5) |  | 16 | 3.1 | 28.6 | 3.7 (–6.3, 13.8) |  | –0.3 (–14.5, 13.9) | 0.963 |
|  |  |  |  | 8w | 15 | 3.6 | 20.8 | 3.0 (–4.5, 10.4) |  | 16 | –7.4 | 20.3 | –6.8 (–14.0, 0.5) |  | 9.7 (–0.7, 20.1) | 0.065 |
|  |  |  |  | 12w | 15 | –5.3 | 16.0 | –6.1 (–15.0, 2.8) |  | 16 | 2.8 | 30.6 | 3.5 (–5.2, 12.2) |  | –9.6 (–22.1, 2.9) | 0.126 |
|  |  | % | Rate of change | 4w | 16 | 95.8 | 233.1 | 76.6 (–39.1, 192.2) |  | 16 | 153.3 | 345.5 | 161.4 (46.0, 276.8) |  | –84.8 (–248.3, 78.7) | 0.298 |
|  |  |  |  | 8w | 15 | 118.4 | 291.5 | 107.7 (17.0, 198.4) |  | 16 | 32.0 | 124.9 | 37.8 (–51.1, 126.6) |  | 69.9 (–57.1, 197.0) | 0.269 |
|  |  |  |  | 12w | 15 | –0.7 | 89.1 | –11.5 (–117.4, 94.4) |  | 16 | 146.0 | 328.1 | 151.8 (48.3, 255.3) |  | –163.3 (–311.4, –15.1) | 0.032* |
| Participants whose score of Sleepiness on rising in OSA-MA below the median (14.15 point) at Scr in FAS1 | Dry mouth | mm | Measured value | Scr | 16 | 54.6 | 21.6 | - |  | 16 | 47.4 | 23.5 | - |  | 7.2 (–9.1, 23.5) | 0.374 |
|  |  |  |  | 4w | 16 | 34.8 | 22.6 | 32.7 (23.6, 41.9) |  | 16 | 32.6 | 21.5 | 34.8 (25.6, 44.0) |  | –2.1 (–15.2, 11.0) | 0.747 |
|  |  |  |  | 8w | 16 | 35.8 | 22.1 | 34.8 (24.0, 45.6) |  | 16 | 37.8 | 21.3 | 38.9 (28.1, 49.7) |  | –4.1 (–19.5, 11.2) | 0.587 |
|  |  |  |  | 12w | 15 | 36.7 | 26.3 | 34.2 (22.5, 45.8) |  | 15 | 44.2 | 23.9 | 46.7 (35.0, 58.3) |  | –12.5 (–29.1, 4.0) | 0.132 |
|  |  |  | Amount of change | 4w | 16 | –19.9 | 17.8 | –18.5 (–27.7, –9.3) |  | 16 | –14.9 | 21.6 | –16.4 (–25.6, –7.2) |  | –2.1 (–15.2, 11.0) | 0.747 |
|  |  |  |  | 8w | 16 | –18.8 | 19.6 | –16.5 (–27.3, –5.7) |  | 16 | –9.7 | 31.0 | –12.3 (–23.1, –1.5) |  | –4.1 (–19.5, 11.2) | 0.587 |
|  |  |  |  | 12w | 15 | –17.2 | 14.9 | –17.1 (–28.7, –5.5) |  | 15 | –5.3 | 29.7 | –4.6 (–16.2, 7.1) |  | –12.5 (–29.1, 4.0) | 0.132 |
|  |  | % | Rate of change | 4w | 16 | –39.5 | 34.4 | –37.0 (–62.8, –11.3) |  | 16 | –12.8 | 64.9 | –15.6 (–41.4, 10.1) |  | –21.4 (–58.1, 15.2) | 0.242 |
|  |  |  |  | 8w | 16 | –35.7 | 43.7 | –29.0 (–66.8, 8.9) |  | 16 | 21.1 | 112.3 | 13.5 (–24.4, 51.4) |  | –42.4 (–96.4, 11.5) | 0.119 |
|  |  |  |  | 12w | 15 | –39.3 | 34.1 | –34.5 (–76.4, 7.4) |  | 15 | 25.4 | 115.6 | 30.0 (–12.0, 71.9) |  | –64.5 (–124.1, –4.8) | 0.035* |

OSA-MA, OSA sleep inventory MA version; FAS1, full analysis set1; SD, standard deviation; EMM, estimated marginal mean; Δ, Difference between groups (BE group - placebo group); 95%CI, 95% confidence interval; Scr, screening (before consumption); 4w, 4 weeks after consumption; 8w, 8 weeks after consumption; 12w, 12 weeks after consumption; Amount of change, amount of change from Scr; Rate of change, rate of change from Scr. **P* < 0.05

Appendix 6. Comparison of palmar surface temperature (overall analysis; FAS3)

| Items | Unit | Classification | Time point | BE group | | | |  | Placebo group | | | |  | Group comparison | |
| --- | --- | --- | --- | --- | --- | --- | --- | --- | --- | --- | --- | --- | --- | --- | --- |
|  |  |  |  | n | Mean | SD | EMM (95%CI) |  | n | Mean | SD | EMM (95%CI) |  | Δ (95%CI) | P value |
| AUC | °C・h | Measured value | Scr | 29 | 16.53 | 0.80 | - |  | 31 | 16.47 | 0.68 | - |  | 0.07 (–0.32, 0.45) | 0.737 |
|  |  | Amount of change | 12w | 29 | –0.48 | 0.99 | –0.47 (–0.84, –0.10) |  | 31 | –1.19 | 1.06 | –1.20 (–1.56, –0.84) |  | 0.73 (0.22, 1.24) | 0.006* |
|  | % | Rate of change | 12w | 29 | –2.8 | 5.9 | –2.8 (–5.0, –0.5) |  | 31 | –7.1 | 6.5 | –7.2 (–9.4, –5.0) |  | 4.4 (1.3, 7.6) | 0.006* |
| Before load | °C | Measured value | Scr | 29 | 33.93 | 1.49 | - |  | 31 | 33.95 | 1.37 | - |  | –0.02 (–0.76, 0.72) | 0.955 |
|  |  | Amount of change | 12w | 29 | –0.43 | 1.98 | –0.44 (–1.09, 0.20) |  | 31 | –1.78 | 2.34 | –1.77 (–2.40, –1.15) |  | 1.33 (0.43, 2.23) | 0.004* |
|  | % | Rate of change | 12w | 29 | –1.1 | 5.7 | –1.1 (–3.1, 0.8) |  | 31 | –5.1 | 7.0 | –5.1 (–6.9, –3.2) |  | 3.9 (1.3, 6.6) | 0.005* |
| Immediately after load | °C | Measured value | Scr | 29 | 25.91 | 2.46 | - |  | 31 | 25.21 | 1.76 | - |  | 0.70 (–0.41, 1.82) | 0.211 |
|  |  | Amount of change | 12w | 29 | –0.59 | 2.48 | –0.31 (–0.81, 0.19) |  | 31 | –1.21 | 1.63 | –1.47 (–1.95, –0.99) |  | 1.16 (0.46, 1.86) | 0.002* |
|  | % | Rate of change | 12w | 29 | –1.6 | 9.0 | –0.7 (–2.6, 1.3) |  | 31 | –4.5 | 6.2 | –5.4 (–7.3, –3.6) |  | 4.8 (2.1, 7.5) | 0.001* |
| 5 min after load | °C | Measured value | Scr | 29 | 32.87 | 2.05 | - |  | 31 | 32.67 | 1.86 | - |  | 0.20 (–0.82, 1.21) | 0.697 |
|  |  | Amount of change | 12w | 29 | –1.20 | 2.56 | –1.14 (–2.02, –0.26) |  | 31 | –2.59 | 2.73 | –2.65 (–3.50, –1.80) |  | 1.51 (0.29, 2.74) | 0.017* |
|  | % | Rate of change | 12w | 29 | –3.5 | 7.7 | –3.3 (–6.0, –0.6) |  | 31 | –7.7 | 8.3 | –7.9 (–10.5, –5.3) |  | 4.6 (0.8, 8.4) | 0.017* |
| 10 min after load | °C | Measured value | Scr | 29 | 33.66 | 1.78 | - |  | 31 | 33.56 | 1.45 | - |  | 0.10 (–0.75, 0.94) | 0.821 |
|  |  | Amount of change | 12w | 29 | –1.07 | 2.35 | –1.04 (–1.90, –0.19) |  | 31 | –2.66 | 2.50 | –2.69 (–3.52, –1.86) |  | 1.64 (0.45, 2.84) | 0.008* |
|  | % | Rate of change | 12w | 29 | –3.1 | 6.9 | –3.0 (–5.6, –0.4) |  | 31 | –7.8 | 7.4 | –7.9 (–10.4, –5.4) |  | 4.9 (1.4, 8.5) | 0.008* |
| 20 min after load | °C | Measured value | Scr | 29 | 34.08 | 1.54 | - |  | 31 | 34.07 | 1.41 | - |  | 0.00 (–0.76, 0.77) | 0.992 |
|  |  | Amount of change | 12w | 29 | –0.95 | 2.10 | –0.95 (–1.76, –0.14) |  | 31 | –2.42 | 2.33 | –2.42 (–3.20, –1.64) |  | 1.47 (0.34, 2.59) | 0.012* |
|  | % | Rate of change | 12w | 29 | –2.7 | 6.1 | –2.7 (–5.1, –0.3) |  | 31 | –7.0 | 6.9 | –7.0 (–9.4, –4.7) |  | 4.3 (1.0, 7.6) | 0.012* |
| 30 min after load | °C | Measured value | Scr | 29 | 33.89 | 1.76 | - |  | 31 | 33.81 | 1.48 | - |  | 0.08 (–0.77, 0.92) | 0.855 |
|  |  | Amount of change | 12w | 29 | –0.77 | 2.17 | –0.75 (–1.51, 0.02) |  | 31 | –2.21 | 2.21 | –2.23 (–2.97, –1.49) |  | 1.49 (0.42, 2.55) | 0.007* |
|  | % | Rate of change | 12w | 29 | –2.2 | 6.4 | –2.1 (–4.4, 0.2) |  | 31 | –6.4 | 6.5 | –6.5 (–8.7, –4.3) |  | 4.4 (1.2, 7.5) | 0.008* |

SD, standard deviation; EMM, estimated marginal mean; Δ, Difference between groups (BE group - placebo group); 95%CI, 95% confidence interval

Scr, screening (before consumption); 12w, 12 weeks after consumption; Amount of change, amount of change from Scr; Rate of change, rate of change from Scr. **P* < 0.05

Appendix 7. Comparison of palmar surface temperature (females of FAS3)

| Items | Unit | Classification | Time point | BE group | | | |  | Placebo group | | | |  | Group comparison | |
| --- | --- | --- | --- | --- | --- | --- | --- | --- | --- | --- | --- | --- | --- | --- | --- |
|  |  |  |  | n | Mean | SD | EMM (95%CI) |  | n | Mean | SD | EMM (95%CI) |  | Δ (95%CI) | P value |
| AUC | °C・h | Measured value | Scr | 16 | 16.47 | 0.70 | - |  | 15 | 16.43 | 0.72 | - |  | 0.03 (–0.49, 0.55) | 0.907 |
|  |  |  | 12w | 16 | 15.76 | 1.10 | 15.76 (15.24, 16.28) |  | 15 | 15.27 | 0.97 | 15.27 (14.74, 15.81) |  | 0.48 (–0.27, 1.23) | 0.196 |
|  |  | Amount of change | 12w | 16 | –0.70 | 1.17 | –0.69 (–1.21, –0.17) |  | 15 | –1.17 | 1.00 | –1.18 (–1.71, –0.64) |  | 0.48 (–0.27, 1.23) | 0.196 |
|  | % | Rate of change | 12w | 16 | –4.2 | 7.0 | –4.1 (–7.3, –0.9) |  | 15 | –7.0 | 6.0 | –7.1 (–10.3, –3.8) |  | 3.0 (–1.6, 7.5) | 0.193 |
| Before load | °C | Measured value | Scr | 16 | 33.62 | 1.47 | - |  | 15 | 33.91 | 1.46 | - |  | –0.29 (–1.37, 0.79) | 0.588 |
|  |  |  | 12w | 16 | 32.84 | 1.58 | 32.84 (32.00, 33.68) |  | 15 | 32.11 | 1.64 | 32.12 (31.25, 32.98) |  | 0.72 (–0.49, 1.93) | 0.232 |
|  |  | Amount of change | 12w | 16 | –0.78 | 2.42 | –0.92 (–1.76, –0.08) |  | 15 | –1.80 | 1.95 | –1.64 (–2.51, –0.77) |  | 0.72 (–0.49, 1.93) | 0.232 |
|  | % | Rate of change | 12w | 16 | –2.1 | 6.8 | –2.5 (–4.9, –0.0) |  | 15 | –5.2 | 5.7 | –4.7 (–7.3, –2.2) |  | 2.3 (–1.3, 5.8) | 0.202 |
| Immediately after load | °C | Measured value | Scr | 16 | 25.68 | 2.77 | - |  | 15 | 25.77 | 1.58 | - |  | –0.09 (–1.75, 1.57) | 0.912 |
|  |  |  | 12w | 16 | 25.43 | 1.61 | 25.44 (24.78, 26.10) |  | 15 | 24.19 | 0.96 | 24.19 (23.50, 24.87) |  | 1.26 (0.31, 2.21) | 0.011* |
|  |  | Amount of change | 12w | 16 | –0.25 | 2.76 | –0.28 (–0.94, 0.38) |  | 15 | –1.58 | 1.51 | –1.54 (–2.22, –0.86) |  | 1.26 (0.31, 2.21) | 0.011* |
|  | % | Rate of change | 12w | 16 | –0.1 | 10.1 | –0.2 (–2.7, 2.2) |  | 15 | –5.9 | 5.7 | –5.7 (–8.3, –3.2) |  | 5.5 (1.9, 9.1) | 0.004* |
| 5 min after load | °C | Measured value | Scr | 16 | 32.45 | 1.96 | - |  | 15 | 32.60 | 2.01 | - |  | –0.15 (–1.61, 1.31) | 0.833 |
|  |  |  | 12w | 16 | 30.83 | 2.21 | 30.84 (29.69, 32.00) |  | 15 | 29.77 | 2.26 | 29.76 (28.57, 30.95) |  | 1.09 (–0.57, 2.75) | 0.191 |
|  |  | Amount of change | 12w | 16 | –1.61 | 3.11 | –1.68 (–2.83, –0.52) |  | 15 | –2.83 | 2.37 | –2.76 (–3.95, –1.57) |  | 1.09 (–0.57, 2.75) | 0.191 |
|  | % | Rate of change | 12w | 16 | –4.6 | 9.3 | –4.8 (–8.3, –1.3) |  | 15 | –8.5 | 7.1 | –8.3 (–12.0, –4.7) |  | 3.5 (–1.6, 8.6) | 0.167 |
| 10 min after load | °C | Measured value | Scr | 16 | 33.58 | 1.68 | - |  | 15 | 33.52 | 1.44 | - |  | 0.06 (–1.09, 1.21) | 0.919 |
|  |  |  | 12w | 16 | 31.80 | 2.45 | 31.79 (30.58, 33.00) |  | 15 | 30.89 | 2.28 | 30.90 (29.65, 32.15) |  | 0.89 (–0.86, 2.63) | 0.305 |
|  |  | Amount of change | 12w | 16 | –1.78 | 2.74 | –1.76 (–2.98, –0.55) |  | 15 | –2.63 | 2.42 | –2.65 (–3.91, –1.40) |  | 0.89 (–0.86, 2.63) | 0.305 |
|  | % | Rate of change | 12w | 16 | –5.1 | 8.0 | –5.1 (–8.7, –1.5) |  | 15 | –7.7 | 7.1 | –7.8 (–11.6, –4.1) |  | 2.7 (–2.5, 7.9) | 0.296 |
| 20 min after load | °C | Measured value | Scr | 16 | 34.03 | 1.19 | - |  | 15 | 33.94 | 1.51 | - |  | 0.09 (–0.91, 1.09) | 0.857 |
|  |  |  | 12w | 16 | 32.59 | 2.53 | 32.57 (31.39, 33.75) |  | 15 | 31.69 | 2.19 | 31.72 (30.50, 32.94) |  | 0.85 (–0.85, 2.54) | 0.313 |
|  |  | Amount of change | 12w | 16 | –1.44 | 2.50 | –1.41 (–2.59, –0.24) |  | 15 | –2.24 | 2.17 | –2.26 (–3.48, –1.05) |  | 0.85 (–0.85, 2.54) | 0.313 |
|  | % | Rate of change | 12w | 16 | –4.2 | 7.3 | –4.1 (–7.6, –0.6) |  | 15 | –6.5 | 6.3 | –6.6 (–10.2, –3.0) |  | 2.5 (–2.5, 7.5) | 0.320 |
| 30 min after load | °C | Measured value | Scr | 16 | 33.86 | 1.31 | - |  | 15 | 33.58 | 1.51 | - |  | 0.28 (–0.77, 1.33) | 0.588 |
|  |  |  | 12w | 16 | 32.76 | 2.38 | 32.70 (31.62, 33.79) |  | 15 | 31.63 | 1.87 | 31.69 (30.57, 32.81) |  | 1.01 (–0.55, 2.57) | 0.194 |
|  |  | Amount of change | 12w | 16 | –1.10 | 2.46 | –1.02 (–2.10, 0.06) |  | 15 | –1.95 | 1.99 | –2.03 (–3.15, –0.91) |  | 1.01 (–0.55, 2.57) | 0.194 |
|  | % | Rate of change | 12w | 16 | –3.2 | 7.2 | –2.9 (–6.1, 0.2) |  | 15 | –5.7 | 5.8 | –5.9 (–9.2, –2.6) |  | 3.0 (–1.6, 7.6) | 0.195 |

SD, standard deviation; EMM, estimated marginal mean; Δ, Difference between groups (BE group - placebo group); 95%CI, 95% confidence interval; Scr, screening (before consumption); 4w, 4 weeks after consumption; 8w, 8 weeks after consumption; 12w, 12 weeks after consumption; Amount of change, amount of change from Scr; Rate of change, rate of change from Scr. **P* < 0.05

Appendix 8. Percentage of cases in which urinalysis and peripheral blood tests measurements were within the reference values at the screening and their values deviated outside of the reference values after the consumption

| Items | BE group | | |  | Placebo group | | |  | Group comparison | | |
| --- | --- | --- | --- | --- | --- | --- | --- | --- | --- | --- | --- |
|  | n | Number of cases | Percentage of cases (%) |  | n | Number of cases | Percentage of cases (%) |  | Δ (95%CI) | χ^2^ value | P value |
| Urinary protein | 30 | 0 | 0.0 |  | 31 | 1 | 3.2 |  | –3.2 (–9.6, 3.1) | 0.984 | 0.321 |
| Urinary glucose | 30 | 1 | 3.3 |  | 31 | 0 | 0.0 |  | 3.3 (–3.0, 9.7) | 1.051 | 0.305 |
| Urinary pH | 30 | 0 | 0.0 |  | 31 | 0 | 0.0 |  | NA | NA | NA |
| Urinary occult blood | 30 | 1 | 3.3 |  | 31 | 1 | 3.2 |  | 0.1 (–8.8, 9.0) | 0.001 | 0.981 |
| Leukocyte count | 30 | 3 | 10.0 |  | 31 | 3 | 9.7 |  | 0.3 (–14.6, 15.3) | 0.002 | 0.966 |
| Erythrocyte count | 30 | 2 | 6.7 |  | 31 | 1 | 3.2 |  | 3.4 (–7.4, 14.3) | 0.386 | 0.534 |
| Hemoglobin | 30 | 3 | 10.0 |  | 31 | 2 | 6.5 |  | 3.5 (–10.2, 17.3) | 0.255 | 0.614 |
| Hematocrit | 30 | 1 | 3.3 |  | 31 | 1 | 3.2 |  | 0.1 (–8.8, 9.0) | 0.001 | 0.981 |
| Platelet count | 30 | 0 | 0.0 |  | 31 | 2 | 6.5 |  | –6.5 (–15.4, 2.5) | 2.001 | 0.157 |
| Aspartate aminotransferase | 30 | 0 | 0.0 |  | 31 | 0 | 0.0 |  | NA | NA | NA |
| Alanine aminotransferase | 30 | 2 | 6.7 |  | 31 | 0 | 0.0 |  | 6.7 (–2.3, 15.6) | 2.137 | 0.144 |
| γ-glutamyl transpeptidase | 30 | 2 | 6.7 |  | 31 | 0 | 0.0 |  | 6.7 (–2.3, 15.6) | 2.137 | 0.144 |
| Total bilirubin | 30 | 1 | 3.3 |  | 31 | 2 | 6.5 |  | –3.1 (–14.0, 7.7) | 0.317 | 0.573 |
| Total protein | 30 | 2 | 6.7 |  | 31 | 2 | 6.5 |  | 0.2 (–12.2, 12.6) | 0.001 | 0.973 |
| Urea nitrogen | 30 | 3 | 10.0 |  | 31 | 5 | 16.1 |  | –6.1 (–23.1, 10.8) | 0.503 | 0.478 |
| Creatinine | 30 | 0 | 0.0 |  | 31 | 0 | 0.0 |  | NA | NA | NA |
| Uric acid | 30 | 0 | 0.0 |  | 31 | 0 | 0.0 |  | NA | NA | NA |
| Sodium | 30 | 0 | 0.0 |  | 31 | 0 | 0.0 |  | NA | NA | NA |
| Potassium | 30 | 4 | 13.3 |  | 31 | 4 | 12.9 |  | 0.4 (–16.5, 17.4) | 0.002 | 0.960 |
| Chlorine | 30 | 0 | 0.0 |  | 31 | 1 | 3.2 |  | –3.2 (–9.6, 3.1) | 0.984 | 0.321 |
| Serum amylase | 30 | 1 | 3.3 |  | 31 | 0 | 0.0 |  | 3.3 (–3.0, 9.7) | 1.051 | 0.305 |
| Total cholesterol | 30 | 2 | 6.7 |  | 31 | 5 | 16.1 |  | –9.5 (–25.5, 6.5) | 1.344 | 0.246 |
| High-density lipoprotein cholesterol | 30 | 3 | 10.0 |  | 31 | 2 | 6.5 |  | 3.5 (–10.2, 17.3) | 0.255 | 0.614 |
| Low-density lipoprotein cholesterol | 30 | 2 | 6.7 |  | 31 | 3 | 9.7 |  | –3.0 (–16.8, 10.8) | 0.184 | 0.668 |
| Triglycerides | 30 | 5 | 16.7 |  | 31 | 1 | 3.2 |  | 13.4 (–1.5, 28.4) | 3.106 | 0.078 |
| Glucose | 30 | 2 | 6.7 |  | 31 | 1 | 3.2 |  | 3.4 (–7.4, 14.3) | 0.386 | 0.534 |
| Hemoglobin A1c (NGSP) | 30 | 1 | 3.3 |  | 31 | 0 | 0.0 |  | 3.3 (–3.0, 9.7) | 1.051 | 0.305 |

Δ, Difference between groups (BE group - placebo group); 95%CI, 95% confidence interval; NA: Not Available

Appendix 9. Summary of anthropometric and physical examinations

| Items | Unit | Time point | BE group | | | | |  | Placebo group | | | | |
| --- | --- | --- | --- | --- | --- | --- | --- | --- | --- | --- | --- | --- | --- |
|  |  |  | n | Mean | SD | Med, Min, Max | 95%CI |  | n | Mean | SD | Med, Min, Max | 95%CI |
| Height | cm | Scr | 30 | 167.3 | 8.2 | 164.7, 155.2, 183.4 | (164.3, 170.4) |  | 31 | 166.7 | 8.3 | 164.3, 150.4, 180.6 | (163.6, 169.7) |
|  |  | 12w | 0 | - | - | - | - |  | 0 | - | - | - | - |
| Body weight | kg | Scr | 30 | 62.6 | 9.1 | 60.3, 51.1, 89.2 | (59.2, 66.0) |  | 31 | 63.0 | 13.5 | 62.2, 45.4, 96.5 | (58.1, 68.0) |
|  |  | 12w | 30 | 63.0 | 9.4 | 61.1, 49.9, 88.0 | (59.5, 66.5) |  | 31 | 63.1 | 14.2 | 60.6, 45.4, 96.5 | (57.9, 68.3) |
| Body mass index | kg/m^2^ | Scr | 30 | 22.3 | 2.4 | 22.2, 18.5, 30.0 | (21.4, 23.2) |  | 31 | 22.5 | 3.2 | 21.7, 17.9, 31.0 | (21.3, 23.7) |
|  |  | 12w | 30 | 22.5 | 2.4 | 22.5, 18.3, 29.6 | (21.6, 23.3) |  | 31 | 22.5 | 3.5 | 21.5, 17.2, 31.0 | (21.2, 23.8) |
| Systolic blood pressure | mmHg | Scr | 30 | 113.5 | 12.6 | 115.5, 91.0, 133.0 | (108.8, 118.2) |  | 31 | 108.4 | 12.7 | 105.0, 91.0, 136.0 | (103.7, 113.1) |
|  |  | 12w | 30 | 111.2 | 13.1 | 111.0, 91.0, 143.0 | (106.3, 116.1) |  | 31 | 110.5 | 12.2 | 111.0, 89.0, 136.0 | (106.1, 115.0) |
| Diastolic blood pressure | mmHg | Scr | 30 | 72.6 | 10.0 | 73.0, 52.0, 92.0 | (68.9, 76.4) |  | 31 | 70.1 | 9.7 | 68.0, 56.0, 93.0 | (66.6, 73.7) |
|  |  | 12w | 30 | 73.0 | 11.1 | 71.0, 59.0, 106.0 | (68.8, 77.1) |  | 31 | 71.7 | 10.8 | 68.0, 56.0, 96.0 | (67.8, 75.7) |

SD, Standard deviation; Med, Median; Min, Minimum; Max, Maximum; 95%CI, 95% confidence interval; Scr, screening (before consumption); 12w, 12 weeks after consumption

Appendix 10. Summary of urinalysis

| Items | Time point | Result | BE group | |  | Placebo group | |
| --- | --- | --- | --- | --- | --- | --- | --- |
|  |  |  | Number of cases | Percentage of cases (%) |  | Number of cases | Percentage of cases (%) |
| Urinary protein | Scr | (-) | 29 | 96.7 |  | 30 | 96.8 |
|  |  | (±) | 1 | 3.3 |  | 1 | 3.2 |
|  |  | (+) | 0 | 0.0 |  | 0 | 0.0 |
|  |  | (2+) | 0 | 0.0 |  | 0 | 0.0 |
|  |  | (3+) | 0 | 0.0 |  | 0 | 0.0 |
|  | 12w | (-) | 30 | 100.0 |  | 30 | 96.8 |
|  |  | (±) | 0 | 0.0 |  | 1 | 3.2 |
|  |  | (+) | 0 | 0.0 |  | 0 | 0.0 |
|  |  | (2+) | 0 | 0.0 |  | 0 | 0.0 |
|  |  | (3+) | 0 | 0.0 |  | 0 | 0.0 |
| Urinary glucose | Scr | (-) | 30 | 100.0 |  | 31 | 100.0 |
|  |  | (±) | 0 | 0.0 |  | 0 | 0.0 |
|  |  | (+) | 0 | 0.0 |  | 0 | 0.0 |
|  |  | (2+) | 0 | 0.0 |  | 0 | 0.0 |
|  |  | (3+) | 0 | 0.0 |  | 0 | 0.0 |
|  | 12w | (-) | 29 | 96.7 |  | 31 | 100.0 |
|  |  | (±) | 1 | 3.3 |  | 0 | 0.0 |
|  |  | (+) | 0 | 0.0 |  | 0 | 0.0 |
|  |  | (2+) | 0 | 0.0 |  | 0 | 0.0 |
|  |  | (3+) | 0 | 0.0 |  | 0 | 0.0 |
| Urinary pH | Scr | < 5.0 | 0 | 0.0 |  | 0 | 0.0 |
|  |  | 5.0 ~ 7.5 | 30 | 100.0 |  | 31 | 100.0 |
|  |  | > 7.5 | 0 | 0.0 |  | 0 | 0.0 |
|  | 12w | < 5.0 | 0 | 0.0 |  | 0 | 0.0 |
|  |  | 5.0 ~ 7.5 | 30 | 100.0 |  | 31 | 100.0 |
|  |  | > 7.5 | 0 | 0.0 |  | 0 | 0.0 |
| Urinary occult blood | Scr | (-) | 30 | 100.0 |  | 27 | 87.1 |
|  |  | (±) | 0 | 0.0 |  | 1 | 3.2 |
|  |  | (+) | 0 | 0.0 |  | 2 | 6.5 |
|  |  | (2+) | 0 | 0.0 |  | 0 | 0.0 |
|  |  | (3+) | 0 | 0.0 |  | 1 | 3.2 |
|  | 12w | (-) | 29 | 96.7 |  | 30 | 96.8 |
|  |  | (±) | 0 | 0.0 |  | 1 | 3.2 |
|  |  | (+) | 0 | 0.0 |  | 0 | 0.0 |
|  |  | (2+) | 0 | 0.0 |  | 0 | 0.0 |
|  |  | (3+) | 1 | 3.3 |  | 0 | 0.0 |

Scr, screening (before consumption); 12w, 12 weeks after consumption

Appendix 11. Blood test findings

| Item | Unit | Time point | Category | Lower limit of reference value | Upper limit of reference value | BE group | | | | |  | Placebo group | | | | |
| --- | --- | --- | --- | --- | --- | --- | --- | --- | --- | --- | --- | --- | --- | --- | --- | --- |
|  |  |  |  |  |  | n | Mean | SD | Med, Min, Max | 95%CI |  | n | Mean | SD | Med, Min, Max | 95%CI |
| White blood cell count | /μL | Scr | MIX | 3300 | 9000 | 30 | 5260 | 1798 | 4,550, 3,100, 9,000 | (4,589, 5,931) |  | 31 | 5303 | 1675 | 5,200, 2,600, 9,700 | (4,689, 5,918) |
|  |  | 12w | MIX | 3300 | 9000 | 30 | 5333 | 2007 | 5,200, 2,900, 12,800 | (4,584, 6,083) |  | 31 | 5248 | 2069 | 5,000, 2,700, 14,100 | (4,490, 6,007) |
| Red blood cell count | ×10^4^/μL | Scr | MIX | - | - | 30 | 449 | 47 | 437, 370, 560 | (432, 467) |  | 31 | 442 | 52 | 439, 312, 545 | (423, 461) |
|  |  |  | M | 430 | 570 | 14 | 475 | 52 | 491, 374, 560 | (445, 506) |  | 16 | 471 | 43 | 484, 381, 545 | (448, 494) |
|  |  |  | F | 380 | 500 | 16 | 427 | 25 | 423, 370, 476 | (413, 440) |  | 15 | 411 | 41 | 420, 312, 473 | (388, 433) |
|  |  | 12w | MIX | - | - | 30 | 450 | 46 | 451, 358, 534 | (433, 467) |  | 31 | 453 | 47 | 453, 369, 572 | (435, 470) |
|  |  |  | M | 430 | 570 | 14 | 473 | 46 | 487, 376, 534 | (446, 499) |  | 16 | 478 | 45 | 470, 369, 572 | (454, 502) |
|  |  |  | F | 380 | 500 | 16 | 430 | 37 | 429, 358, 488 | (411, 450) |  | 15 | 425 | 33 | 419, 372, 494 | (407, 444) |
| Hemoglobin | g/dL | Scr | MIX | - | - | 30 | 13.6 | 1.3 | 13.4, 11.4, 16.4 | (13.1, 14.0) |  | 31 | 13.4 | 1.8 | 13.5, 7.3, 16.7 | (12.8, 14.1) |
|  |  |  | M | 13.5 | 17.5 | 14 | 14.4 | 1.2 | 14.6, 12.3, 16.4 | (13.7, 15.1) |  | 16 | 14.6 | 1.1 | 14.4, 12.4, 16.7 | (14.0, 15.2) |
|  |  |  | F | 11.5 | 15.0 | 16 | 12.8 | 0.8 | 13.1, 11.4, 14.4 | (12.4, 13.3) |  | 15 | 12.2 | 1.5 | 12.3, 7.3, 13.6 | (11.4, 13.1) |
|  |  | 12w | MIX | - | - | 30 | 13.5 | 1.3 | 13.7, 10.2, 16.1 | (13.1, 14.0) |  | 31 | 13.7 | 1.4 | 13.8, 11.1, 17.9 | (13.2, 14.3) |
|  |  |  | M | 13.5 | 17.5 | 14 | 14.2 | 1.1 | 14.1, 12.3, 16.1 | (13.6, 14.9) |  | 16 | 14.7 | 1.2 | 14.8, 12.5, 17.9 | (14.0, 15.3) |
|  |  |  | F | 11.5 | 15.0 | 16 | 12.9 | 1.1 | 13.1, 10.2, 14.2 | (12.3, 13.5) |  | 15 | 12.7 | 0.8 | 12.5, 11.1, 14.0 | (12.3, 13.2) |
| Hematocrit | % | Scr | MIX | - | - | 30 | 42.2 | 3.6 | 41.7, 35.8, 48.8 | (40.8, 43.5) |  | 31 | 42.1 | 5.2 | 42.0, 25.3, 51.8 | (40.2, 44.0) |
|  |  |  | M | 39.7 | 52.4 | 14 | 44.5 | 3.4 | 45.0, 37.3, 48.8 | (42.5, 46.4) |  | 16 | 45.3 | 3.7 | 45.4, 37.2, 51.8 | (43.3, 47.3) |
|  |  |  | F | 34.8 | 45.0 | 16 | 40.1 | 2.3 | 40.6, 35.8, 44.7 | (38.9, 41.4) |  | 15 | 38.7 | 4.2 | 38.7, 25.3, 43.0 | (36.3, 41.0) |
|  |  | 12w | MIX | - | - | 30 | 42.0 | 3.7 | 42.4, 32.4, 50.1 | (40.7, 43.4) |  | 31 | 43.0 | 4.0 | 43.4, 36.2, 54.3 | (41.5, 44.4) |
|  |  |  | M | 39.7 | 52.4 | 14 | 44.0 | 3.2 | 43.2, 39.0, 50.1 | (42.1, 45.9) |  | 16 | 45.5 | 3.5 | 45.7, 36.6, 54.3 | (43.6, 47.4) |
|  |  |  | F | 34.8 | 45.0 | 16 | 40.3 | 3.3 | 41.1, 32.4, 44.7 | (38.6, 42.1) |  | 15 | 40.2 | 2.2 | 40.5, 36.2, 44.0 | (39.0, 41.4) |
| Platelet count | ×10^4^/μL | Scr | MIX | 14.0 | 34.0 | 30 | 26.6 | 5.8 | 25.8, 18.3, 43.6 | (24.4, 28.8) |  | 31 | 25.7 | 5.2 | 25.6, 13.6, 38.3 | (23.8, 27.6) |
|  |  | 12w | MIX | 14.0 | 34.0 | 30 | 27.8 | 5.3 | 27.9, 18.6, 41.1 | (25.8, 29.8) |  | 31 | 26.5 | 5.4 | 25.6, 15.6, 38.4 | (24.6, 28.5) |
| Aspartate aminotransferase | U/L | Scr | MIX | 10 | 40 | 30 | 18 | 4 | 18, 13, 26 | (17, 20) |  | 31 | 21 | 8 | 18, 10, 44 | (18, 23) |
|  |  | 12w | MIX | 10 | 40 | 30 | 19 | 5 | 18, 11, 37 | (17, 21) |  | 31 | 21 | 6 | 19, 12, 37 | (18, 23) |
| Alanine aminotransferase | U/L | Scr | MIX | 5 | 45 | 30 | 17 | 8 | 16, 6, 42 | (14, 20) |  | 31 | 17 | 9 | 14, 4, 46 | (14, 20) |
|  |  | 12w | MIX | 5 | 45 | 30 | 18 | 10 | 15, 4, 49 | (14, 21) |  | 31 | 18 | 8 | 16, 7, 40 | (15, 21) |
| γ-glutamyl transpeptidase | U/L | Scr | MIX | - | - | 30 | 25 | 26 | 19, 9, 155 | (15, 35) |  | 31 | 30 | 32 | 20, 8, 142 | (18, 41) |
|  |  |  | M | 0 | 80 | 14 | 22 | 7 | 20, 12, 34 | (18, 26) |  | 16 | 41 | 41 | 23, 16, 142 | (19, 63) |
|  |  |  | F | 0 | 30 | 16 | 27 | 35 | 19, 9, 155 | (9, 46) |  | 15 | 17 | 10 | 14, 8, 46 | (12, 23) |
|  |  | 12w | MIX | - | - | 30 | 29 | 31 | 18, 9, 178 | (17, 41) |  | 31 | 27 | 24 | 20, 9, 111 | (18, 36) |
|  |  |  | M | 0 | 80 | 14 | 25 | 13 | 23, 13, 62 | (18, 33) |  | 16 | 36 | 30 | 23, 16, 111 | (20, 52) |
|  |  |  | F | 0 | 30 | 16 | 32 | 41 | 18, 9, 178 | (10, 54) |  | 15 | 18 | 11 | 14, 9, 51 | (12, 24) |
| Total bilirubin | mg/dL | Scr | MIX | 0.2 | 1.2 | 30 | 0.8 | 0.2 | 0.8, 0.4, 1.3 | (0.7, 0.9) |  | 31 | 0.8 | 0.3 | 0.7, 0.3, 1.6 | (0.7, 0.9) |
|  |  | 12w | MIX | 0.2 | 1.2 | 30 | 0.8 | 0.3 | 0.8, 0.4, 1.9 | (0.7, 0.9) |  | 31 | 0.8 | 0.2 | 0.7, 0.4, 1.3 | (0.7, 0.9) |
| Total protein | g/dL | Scr | MIX | 6.7 | 8.3 | 30 | 7.1 | 0.4 | 7.0, 6.2, 7.8 | (6.9, 7.2) |  | 31 | 6.9 | 0.4 | 7.0, 5.9, 7.6 | (6.8, 7.1) |
|  |  | 12w | MIX | 6.7 | 8.3 | 30 | 7.1 | 0.4 | 7.1, 6.2, 7.9 | (6.9, 7.2) |  | 31 | 7.1 | 0.4 | 7.1, 6.3, 7.8 | (6.9, 7.2) |
| Urea nitrogen | mg/dL | Scr | MIX | 8.0 | 20.0 | 30 | 14.0 | 4.0 | 13.9, 6.6, 21.2 | (12.5, 15.5) |  | 31 | 13.4 | 4.4 | 12.7, 7.0, 27.5 | (11.8, 15.0) |
|  |  | 12w | MIX | 8.0 | 20.0 | 30 | 13.2 | 4.3 | 12.7, 5.2, 21.6 | (11.6, 14.8) |  | 31 | 12.9 | 4.6 | 11.3, 6.7, 25.2 | (11.2, 14.6) |
| Creatinine | mg/dL | Scr | MIX | - | - | 30 | 0.73 | 0.13 | 0.70, 0.55, 1.06 | (0.68, 0.78) |  | 31 | 0.74 | 0.17 | 0.73, 0.45, 1.09 | (0.67, 0.80) |
|  |  |  | M | 0.61 | 1.04 | 14 | 0.85 | 0.10 | 0.82, 0.70, 1.06 | (0.79, 0.91) |  | 16 | 0.86 | 0.12 | 0.86, 0.68, 1.09 | (0.80, 0.93) |
|  |  |  | F | 0.47 | 0.79 | 16 | 0.63 | 0.05 | 0.63, 0.55, 0.74 | (0.61, 0.66) |  | 15 | 0.60 | 0.10 | 0.59, 0.45, 0.82 | (0.55, 0.65) |
|  |  | 12w | MIX | - | - | 30 | 0.71 | 0.13 | 0.70, 0.50, 1.04 | (0.65, 0.76) |  | 31 | 0.72 | 0.15 | 0.70, 0.43, 1.03 | (0.66, 0.77) |
|  |  |  | M | 0.61 | 1.04 | 14 | 0.80 | 0.11 | 0.79, 0.65, 1.04 | (0.74, 0.87) |  | 16 | 0.81 | 0.13 | 0.79, 0.63, 1.03 | (0.75, 0.88) |
|  |  |  | F | 0.47 | 0.79 | 16 | 0.62 | 0.09 | 0.62, 0.50, 0.75 | (0.57, 0.67) |  | 15 | 0.62 | 0.11 | 0.61, 0.43, 0.84 | (0.56, 0.68) |
| Uric acid | mg/dL | Scr | MIX | - | - | 30 | 5.0 | 1.1 | 5.0, 3.1, 6.9 | (4.6, 5.4) |  | 31 | 5.2 | 1.4 | 5.3, 1.6, 9.0 | (4.6, 5.7) |
|  |  |  | M | 3.8 | 7.0 | 14 | 5.6 | 0.9 | 5.6, 4.0, 6.9 | (5.1, 6.1) |  | 16 | 6.1 | 1.0 | 5.9, 4.8, 9.0 | (5.6, 6.6) |
|  |  |  | F | 2.5 | 7.0 | 16 | 4.5 | 1.1 | 4.6, 3.1, 6.7 | (3.9, 5.1) |  | 15 | 4.2 | 1.2 | 4.1, 1.6, 6.5 | (3.5, 4.9) |
|  |  | 12w | MIX | - | - | 30 | 4.8 | 1.0 | 4.9, 3.1, 7.0 | (4.4, 5.2) |  | 31 | 5.2 | 1.5 | 5.2, 2.2, 10.3 | (4.7, 5.8) |
|  |  |  | M | 3.8 | 7.0 | 14 | 5.3 | 0.7 | 5.2, 4.3, 6.8 | (4.9, 5.7) |  | 16 | 6.1 | 1.3 | 6.0, 4.6, 10.3 | (5.4, 6.7) |
|  |  |  | F | 2.5 | 7.0 | 16 | 4.4 | 1.0 | 4.1, 3.1, 7.0 | (3.8, 5.0) |  | 15 | 4.3 | 1.1 | 4.2, 2.2, 6.3 | (3.7, 5.0) |
| Sodium | mEq/L | Scr | MIX | 137 | 147 | 30 | 141 | 2 | 141, 135, 145 | (140, 141) |  | 31 | 141 | 2 | 141, 132, 144 | (140, 142) |
|  |  | 12w | MIX | 137 | 147 | 30 | 141 | 2 | 141, 138, 145 | (141, 142) |  | 31 | 141 | 2 | 141, 135, 145 | (140, 142) |
| Potassium | mEq/L | Scr | MIX | 3.5 | 5.0 | 30 | 4.1 | 0.3 | 4.1, 3.5, 4.8 | (4.0, 4.2) |  | 31 | 4.1 | 0.3 | 4.1, 3.4, 4.8 | (3.9, 4.2) |
|  |  | 12w | MIX | 3.5 | 5.0 | 30 | 4.5 | 0.6 | 4.3, 3.8, 5.8 | (4.2, 4.7) |  | 31 | 4.3 | 0.5 | 4.2, 3.6, 6.1 | (4.1, 4.5) |
| Chloride | mEq/L | Scr | MIX | 98 | 108 | 30 | 103 | 2 | 103, 98, 107 | (102, 104) |  | 31 | 103 | 2 | 103, 98, 107 | (102, 104) |
|  |  | 12w | MIX | 98 | 108 | 30 | 103 | 2 | 103, 100, 107 | (103, 104) |  | 31 | 102 | 3 | 103, 97, 107 | (101, 103) |
| Serum amylase | U/L | Scr | MIX | 40 | 122 | 30 | 73 | 26 | 71, 44, 180 | (63, 82) |  | 31 | 73 | 19 | 70, 49, 127 | (66, 80) |
|  |  | 12w | MIX | 40 | 122 | 30 | 77 | 27 | 72, 42, 160 | (67, 87) |  | 31 | 74 | 21 | 68, 47, 143 | (66, 81) |
| Total cholesterol | mg/dL | Scr | MIX | 120 | 219 | 30 | 211 | 29 | 210, 154, 285 | (200, 222) |  | 31 | 199 | 37 | 204, 125, 279 | (186, 213) |
|  |  | 12w | MIX | 120 | 219 | 30 | 210 | 28 | 204, 151, 271 | (199, 220) |  | 31 | 201 | 39 | 193, 113, 265 | (186, 215) |
| High density lipoprotein-cholesterol | mg/dL | Scr | MIX | - | - | 30 | 73 | 22 | 72, 33, 120 | (65, 81) |  | 31 | 70 | 17 | 71, 38, 126 | (64, 76) |
|  |  |  | M | 40 | 85 | 14 | 70 | 20 | 72, 40, 110 | (58, 82) |  | 16 | 61 | 12 | 61, 38, 86 | (54, 68) |
|  |  |  | F | 40 | 95 | 16 | 76 | 23 | 79, 33, 120 | (63, 88) |  | 15 | 79 | 16 | 80, 51, 126 | (70, 88) |
|  |  | 12w | MIX | - | - | 30 | 73 | 18 | 75, 38, 107 | (67, 80) |  | 31 | 73 | 19 | 74, 37, 123 | (66, 80) |
|  |  |  | M | 40 | 85 | 14 | 71 | 18 | 74, 40, 99 | (61, 81) |  | 16 | 62 | 14 | 62, 37, 92 | (55, 70) |
|  |  |  | F | 40 | 95 | 16 | 75 | 19 | 77, 38, 107 | (65, 85) |  | 15 | 85 | 17 | 87, 45, 123 | (76, 94) |
| Low density lipoprotein-cholesterol | mg/dL | Scr | MIX | 65 | 139 | 30 | 119 | 27 | 116, 77, 191 | (109, 128) |  | 31 | 111 | 33 | 111, 52, 176 | (99, 123) |
|  |  | 12w | MIX | 65 | 139 | 30 | 119 | 25 | 113, 76, 164 | (110, 128) |  | 31 | 113 | 33 | 115, 56, 169 | (101, 125) |
| Triglyceride | mg/dL | Scr | MIX | 30 | 149 | 30 | 84 | 57 | 65, 26, 276 | (63, 105) |  | 31 | 87 | 58 | 66, 32, 259 | (66, 108) |
|  |  | 12w | MIX | 30 | 149 | 30 | 95 | 73 | 76, 22, 304 | (67, 122) |  | 31 | 81 | 49 | 69, 30, 203 | (63, 99) |
| Glucose | mg/dL | Scr | MIX | 70 | 109 | 30 | 87 | 8 | 86, 78, 110 | (84, 90) |  | 31 | 88 | 13 | 87, 73, 149 | (83, 93) |
|  |  | 12w | MIX | 70 | 109 | 30 | 87 | 11 | 85, 71, 124 | (83, 91) |  | 31 | 88 | 13 | 86, 73, 147 | (84, 93) |
| Hemoglobin A1c (NGSP) | % | Scr | MIX | 4.6 | 6.2 | 30 | 5.4 | 0.3 | 5.4, 4.9, 6.2 | (5.3, 5.5) |  | 31 | 5.2 | 0.3 | 5.2, 4.7, 5.8 | (5.1, 5.3) |
|  |  | 12w | MIX | 4.6 | 6.2 | 30 | 5.4 | 0.4 | 5.3, 4.9, 7.1 | (5.3, 5.5) |  | 31 | 5.3 | 0.3 | 5.3, 4.7, 5.9 | (5.1, 5.4) |

SD, standard deviation; Med, median; Min, minimum; Max, maximum; 95% CI, 95% confidence interval

MIX, both male and female; M, male; F, female; Scr, screening (before consumption); 12w, 12 weeks after consumption
